# Tailor-Made Synthesis of Hydrosilanols, Hydrosiloxanes, and Silanediols Catalyzed by di-Silyl Rhodium(III) and Iridium(III) Complexes

**DOI:** 10.1021/acs.inorgchem.2c03953

**Published:** 2023-02-09

**Authors:** Unai Prieto-Pascual, Antonio Rodríguez-Diéguez, Zoraida Freixa, Miguel A. Huertos

**Affiliations:** † Facultad de Química, Universidad del País Vasco (UPV/EHU), 20018 San Sebastián, Spain; ‡ Departamento de Química Inorgánica, Universidad de Granada, 18071 Granada, Spain; § IKERBASQUE, Basque Foundation for Science, 48011 Bilbao, Spain

## Abstract

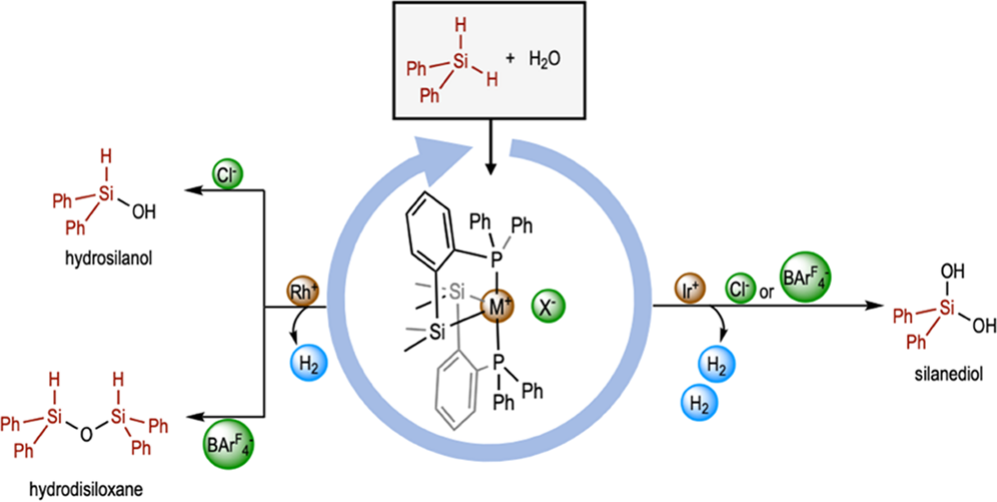

Siloxanes and silanols containing Si–H units are important building blocks for the synthesis of functionalized siloxane materials, and their synthesis is a current challenge. Herein, we report the selective synthesis of hydrosilanols, hydrosiloxanes, and silanodiols depending on the nature of the catalysts and the silane used. Two neutral ({MCl[SiMe_2_(*o*-C_6_H_4_PPh_2_)]_2_}; M = Rh, Ir) and two cationic ({M[SiMe_2_(*o*-C_6_H_4_PPh_2_)]_2_(NCMe)}[BAr^F^_4_]; M = Rh, Ir) have been synthesized and their catalytic behavior toward hydrolysis of secondary silanes has been described. Using the iridium complexes as precatalysts and diphenylsilane as a substrate, the product obtained is diphenylsilanediol. When rhodium complexes are used as precatalysts, it is possible to selectively obtain silanediol, hydrosilanol, and hydrosiloxane depending on the catalysts (neutral or cationic) and the silane substituents.

## Abstract 2

Bidentate silicon-based ligands have been used to synthesize two neutral ({MCl[SiMe_2_(*o*-C_6_H_4_PPh_2_)]_2_}; M = Rh, Ir) and two cationic ({M[SiMe_2_(*o*-C_6_H_4_PPh_2_)]_2_(NCMe)}[BAr_^F^4_]; M = Rh, Ir) complexes. The four new complexes proved to be catalytically active in the hydrolysis of dihydrosilanes, rendering different products, silanediols, hydrosilanols, or hydrosiloxanes, depending on the catalyst/substrate combination.

## Introduction

Silicones (oligo- and polysiloxane materials) are an important class of inorganic materials that find many industrial applications due to their outstanding physicochemical properties (thermal and light stability and resistance to water and oxidation).^1,2^ There are well-known methods for the synthesis of siloxane materials. The most used to date are the ring-opening polymerization of cyclic siloxanes and sol–gel processes (hydrolytic condensation of chlorosilanes (Cl–Si) or alkoxysilanes (RO–Si)).^3^

Nowadays, one of the main challenges in the synthesis of siloxane materials is to find methods to construct highly functionalized siloxanes with a well-defined structure. This could be achieved by using siloxanes, alkoxysilanes, and silanols, containing Si–H moieties as building blocks. Si–H bond is one of the most useful functional groups in silicon chemistry, and there are a large number of well-known reactions to transform this functional group (e.g., *via* hydrosilylation reactions).^4,5^ Moreover, siloxanes with Si–H moieties are also interesting molecules to functionalize organic and inorganic compounds.^6^ Silanols, besides being used in the production of silicon-based polymers,^7^ are important intermediates in organic synthesis (e.g., an efficient organic donor in metal-catalyzed cross-coupling).^8^

In 2010, Kuroda et al. reported a pioneering study on the selective synthesis of hydrosiloxanes through a cross-coupling type reaction catalyzed by BiCl_3_. Unfortunately, the product could not be effectively separated from the catalyst.^9^ Recently, more effective synthetic catalytic methods for the formation of hydrosiloxanes have been reported by reacting dihydrosilanes with silanols catalyzed by gold,^10^ cobalt,^11^ Fe,^12^ or main-group species.^13^ Alternatively, they can be obtained by dealcoholization reaction between silanols and alkoxyhydrosilanes without the use of catalyst or additives.^14^

In the case of the synthesis of silanols,^15^ there are well-established methods based on the hydrolysis of chlorosilanes,^16^ oxidation of hydrosilanes using strong oxidants,^17^ H_2_O_2_^18^ or O_2_^19^ and the metal catalytic hydrolytic oxidation of hydrosilanes with water (also known as hydrolysis of silanes).^20^ Reusable heterogeneous catalysts have also been used in the hydrolysis of silanes.^21^ The importance of the hydrolysis of silanes lies in the atom efficiency of this process. Recent studies have shown that silanols can also be obtained by electrochemical hydrolysis^22^ and enzymatic oxidation^23^ of hydrosilanes.

**Scheme 1:**
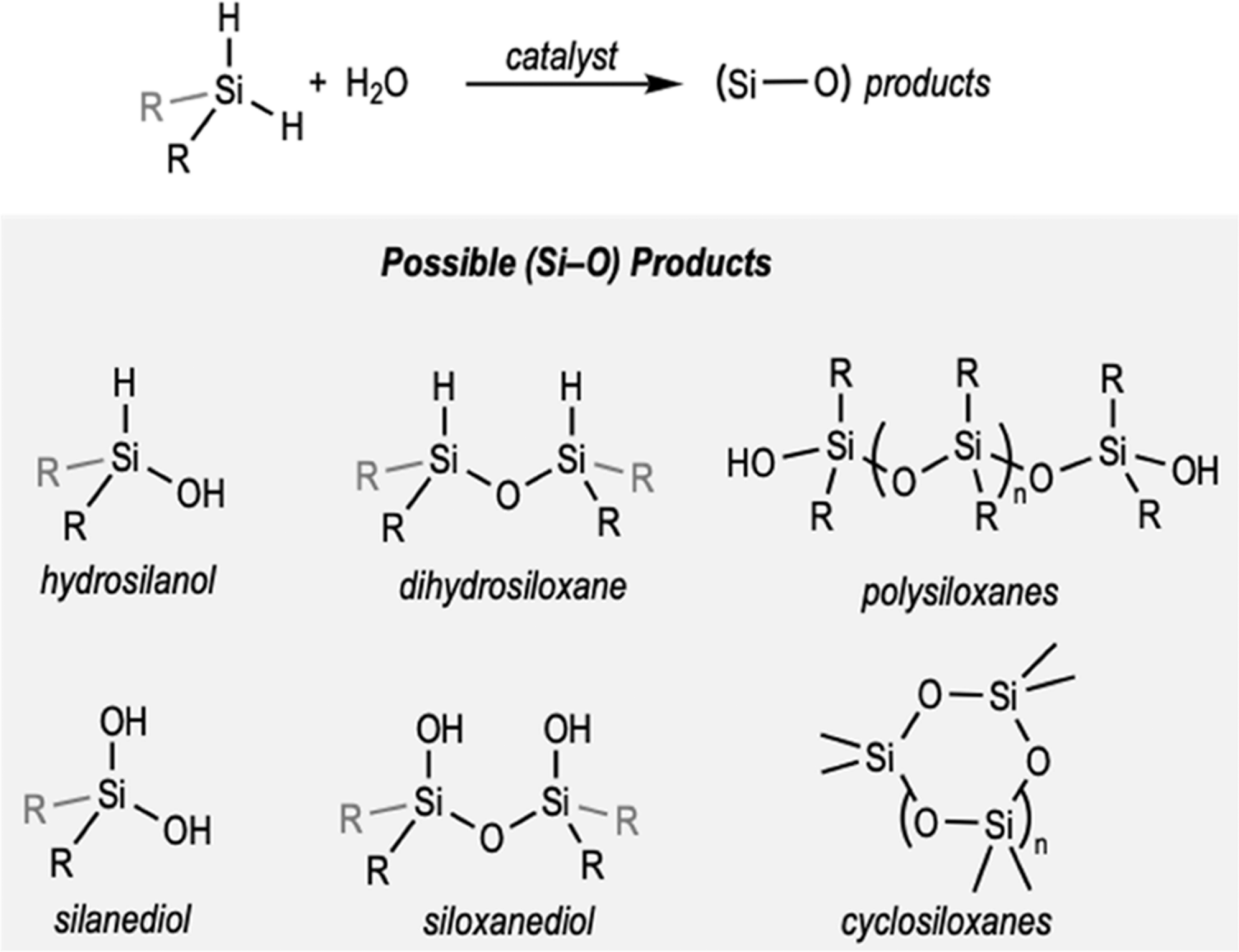
Hydrolysis of Dihydrosilanes and Possible Reaction Products

Focusing on the synthesis of hydrosilanols through metal-catalyzed oxidation of dihydrosilanes using H_2_O, few selective methods have been reported to date.^24^ The problem is the presence of two reactive Si–H and the competitive condensation reactions.^20f,20g,20m,20n^ Thus, the hydrolysis of dihydrosilanes might lead to the formation of several products, as shown in Scheme 1, or mixtures thereof.

Herein, we report the catalytic activity of two neutral ({MCl[SiMe_2_(*o*-C_6_H_4_PPh_2_)]_2_}; M = Rh, Ir) and two cationic ({M[SiMe_2_(*o*-C_6_H_4_PPh_2_)]_2_(NCMe)}[BAr^F^_4_]; M = Rh, Ir) complexes in the hydrolysis of dihydrosilanes. The difference in the structure of each complex, together with the nature of the dihydrosilane used, allowed us to selectively synthesize different types of related Si–O products (Scheme 1).

## Results and Discussion

### Synthesis and Characterization of Neutral and Cationic P,Si-Complexes

**Scheme 2:**
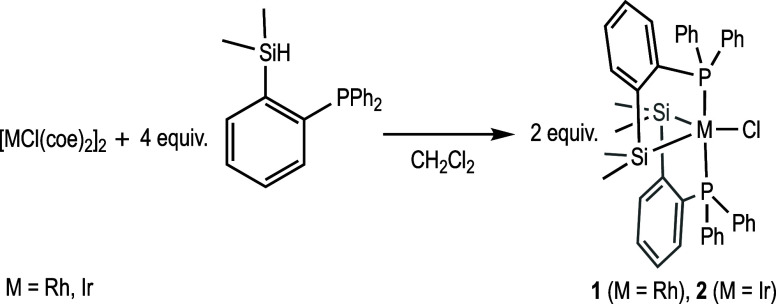
Synthesis of Complexes **1** and **2**

Reaction of [RhCl(coe)_2_]_2_ with 4 equiv of the proligand [Si(H)Me_2_(*o*-C_6_H_4_PPh_2_)] in CH_2_Cl_2_ at room temperature afforded, after 1 h, the air-stable compound {RhCl[SiMe_2_(*o*-C_6_H_4_PPh_2_)]_2_} (**1** in Scheme 2) in good yield (92%), which was characterized in solution by NMR spectroscopy. In the ^1^H NMR spectrum, two singlets are observed for the Si–CH_3_ groups (δ −0.07 and δ −0.43) due to the nonequivalence of the methyl groups on each silicon atom upon coordination to rhodium, as previously observed in a related compound.^25^ The ^31^P{^1^H} NMR spectrum shows a unique signal as a doublet at δ 55.6 (*J*_Rh-P_ = 120 Hz), which indicates that both ligands are equivalent in solution. An isoelectronic iridium complex, {IrCl[SiMe_2_(*o*-C_6_H_4_PPh_2_)]_2_} (**2** in Scheme 2), was synthesized by reaction of [IrCl(coe)_2_]_2_ with 4 equiv of [Si(H)Me_2_(*o*-C_6_H_4_PPh_2_)] under the same reaction conditions (78% yield). The ^1^H NMR spectroscopic pattern of complex **2** in solution is similar to that observed for complex **1** (see Experimental Section and Supporting Information, SI for more details). The ^31^P{^1^H} NMR shows a singlet at δ 54.4 that indicates equivalent triarylphosphine groups.

**Figure 1:**
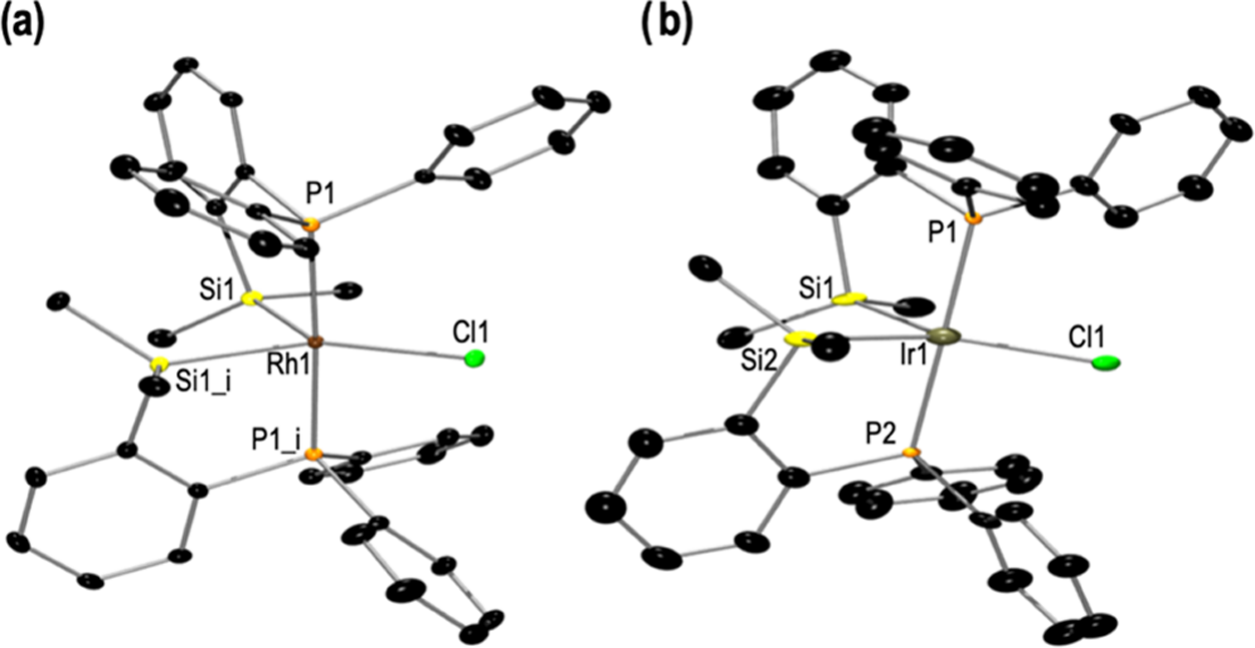
Displacement ellipsoids are drawn at a 50% probability level. The hydrogen atoms and solvent molecules are omitted for clarity. (a) Molecular structure of **1**. Selected bond lengths (Å) and angles (°): Rh1–Cl1 2.4357(8), Rh1–Si1 2.3099(6), Rh1–P1 2.3234(6), Si1–Rh1–Si1_i 83.37(3), P1–Rh1–P1_i 177.43(3), P1–Rh1–Si1 84.11(2), P1–Rh1–Cl1 91.28(2), and Si1–Rh1–Cl1 138.32(2). (b) Molecular structure of **2**. Selected bond lengths (Å) and angles (°): Ir1–Cl1 2.399(4), Ir1–P1 2.318(4), Ir1–P2 2.318(4), Ir1–Si1 2.319(5), Ir1–Si2 2.325(5), P1–Ir1–P2 178.25(15), Si1–Ir1–Si2 83.43(18), Si1–Ir1–P1 83.89(15), Si1–Ir1–P2 95.88(15), Si2–Ir1–P2 84.53(15), Si2–Ir1–P1 94.42(15), Si1–Ir1–Cl1 134.87(18), Si2–Ir1–Cl1 141.70(19), and P1–Ir1–Cl1 90.71(14), P2–Ir1–Cl1 90.17(14).

Compounds **1** and **2** were also characterized by single-crystal X-ray structural determination (Figure 1). The resulting molecular structures are in good agreement with the structures deduced from the spectroscopic data in solution. In the solid state, the geometry about the Rh(III) center in complex **1** can be described as a distorted trigonal bipyramid with the apical positions occupied by the phosphorous atoms (P1 and P1_i) and the equatorial positions occupied by the silicon (Si1 and Si1_i) and the chlorine atoms. The rhodium atom is included in the plane Si1Cl1Si1_i. The sum of the three angles (Cl1–Rh1–Si1, Si1–Rh1–Si1_i, and Si1_i–Rh1–Cl1) being 360° and the P1–Rh1–P1_i angle of 177.43(3)° suggest this geometry. Molecular structure of complex **2**, in the solid state, is isostructural to that described for compound **1** (Figure 1b).

**Scheme 3:**
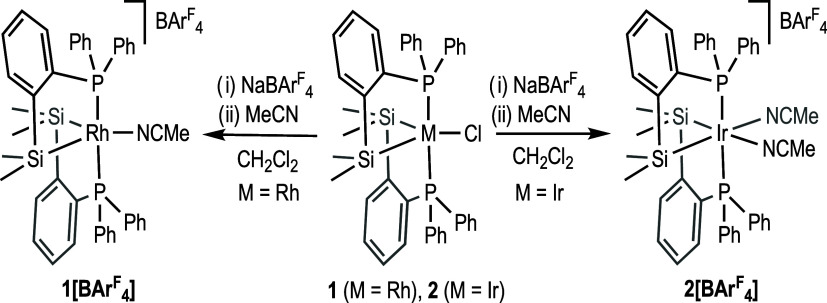
Synthesis of Complexes **1[BAr^F^_4_]** and **2[BAr^F^_4_]**

Treatment of complexes **1** and **2** with an equimolar amount of NaBAr^F^_4_ in CH_2_Cl_2_ and in the presence of a small amount of MeCN yielded the Rh(III) cationic complex {Rh[SiMe_2_(*o*-C_6_H_4_PPh_2_)]_2_(NCMe)}[BAr^F^_4_] (**1[BAr**^F^_4_**]**) and a similar cationic complex with two acetonitrile molecules coordinated to the metal center, {Ir[SiMe_2_(*o*-C_6_H_4_PPh_2_)]_2_(NCMe)_2_}[BAr_4_^F^] (**2[BAr**^F^_4_**]**), respectively (Scheme 3).

Compounds **1[BAr**^F^_4_**]** and **2[BAr**^F^_4_**]** were characterized in solution by NMR spectroscopy (see Experimental Section and SI for more details). Both complexes showed in the ^1^H NMR spectrum two different signals for the Si–CH_3_ groups (δ 0.04 and δ −0.32 for **1[BAr**^F^_4_**]**; δ −0.10 and δ −0.26 for **2[BAr**^F^_4_**]**). The signals assigned to coordinated MeCN were observed at 1.78 ppm (integrating by 3H) in the case of **1[BAr**^F^_4_**]** and at 1.48 (integrating by 6H) in the case of **2[BAr**^F^_4_**]**. The equivalence of both phosphines in solution is confirmed by the presence of only one signal in the ^31^P{^1^H} NMR spectra. They appear as a doublet at 55.5 ppm {*J*_Rh-P_ = 120 Hz} ppm for **1[BAr**^F^_4_**]** and a singlet at 38.1 ppm for **2[BAr**^F^_4_**]**. The spectroscopic data were consistent with a pentacoordinated Rh(III) complex in **1[BAr**^F^_4_**]** and a pseudo-octahedral Ir(III) structure in the case of **2[BAr**^F^_4_**]**, as proposed in Scheme 3.

**Figure 2:**
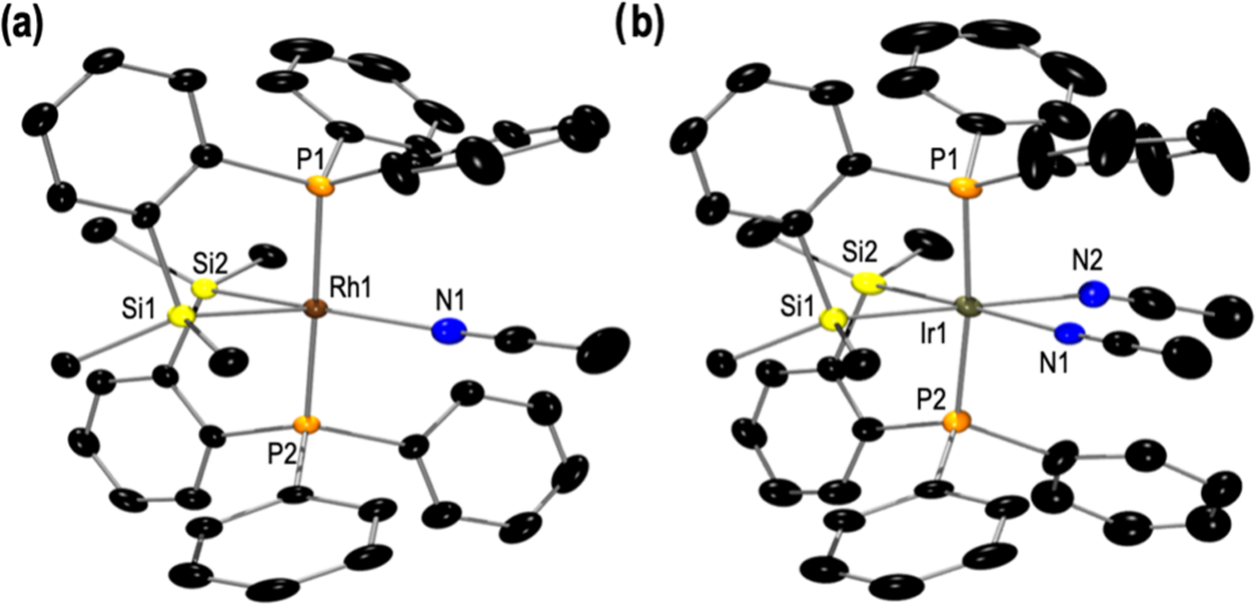
Displacement ellipsoids are drawn at a 50% probability level. The hydrogen atoms and the counteranion [BAr_4_^F^] are omitted for clarity. (a) Molecular structure of **1[BAr**^F^_4_**]**. Selected bond lengths (Å) and angles (°): Rh1–N1 2.163(3), Rh1–Si1 2.341(1), Rh1–Si2 2.311(1), Rh1–P1 2.317(1), Rh1–P2 2.317(1), Si1–Rh1–Si2 86.44(4), Si1–Rh1–N1 107.45(9), Si2–Rh1–N1 165.76(9). (b) Molecular structure of **2[BAr**^F^_4_**]**. Selected bond lengths (Å) and angles (°): Ir1–N1 2.279(4), Ir1–N2 2.148(3), Ir1–Si1 2.356(1), Ir1–Si2 2.366(1), Ir1–P1 2.331(1), Ir1–P2 2.326(1), Si1–Ir1–Si2 89.57(4), Si1–Ir1–N1 90.99(11), Si2–Ir1–N2 92.93(9), N1–Ir1–N2 86.52(14).

These structures were confirmed by X-ray diffraction (Figure 2). Figure 2a shows the solid-state structure of **1[BAr**^F^_4_**]**. The formally Rh(III) center adopts a square pyramid geometry with one of the silicon atoms (Si1) located on the apical position. The plane defined by the other silicon atom (Si2), the two phosphorous atoms, and the acetonitrile ligand (Si2P1P2N1), where is included the rhodium atom, forms the base of the pyramid. The sum of the four angles (Si2–Rh1–P1, P1–Rh1–N1, N1–Rh1–P2, and P2–Rh1–Si2) being 360° suggests this geometry. It is important to note that in solution, both SiP chelate ligands are equivalent. This could be due to a rapid interchange of MeCN located trans to Si2 to be placed trans to Si1.

The solid-state structure of **2[BAr**_**4**_^**F**^**]** is showed in Figure 2b. The resulting molecular structure agrees with the structure deduced from the spectroscopic data in solution. The coordination geometry of the Ir(III) atom is pseudo-octahedral. One of the chelate SiP ligands and one of the acetonitrile (Si1, P1, and N1) are located on one of the faces of the octahedron. The three remaining coordination sites are occupied by the other SiP ligand and MeCN (Si2, P2, and N2). The *trans*-labilizing nature of the silyl group is reflected in long Ir—N bond lengths [N1—Ir1 = 2.536(2) Å and N2—Ir1 = 2.536(2) Å], when compared with other iridium-acetonitrile distances in similar structures.^26^

### Catalytic Hydrolysis of Diphenylsilane

**Figure 3:**
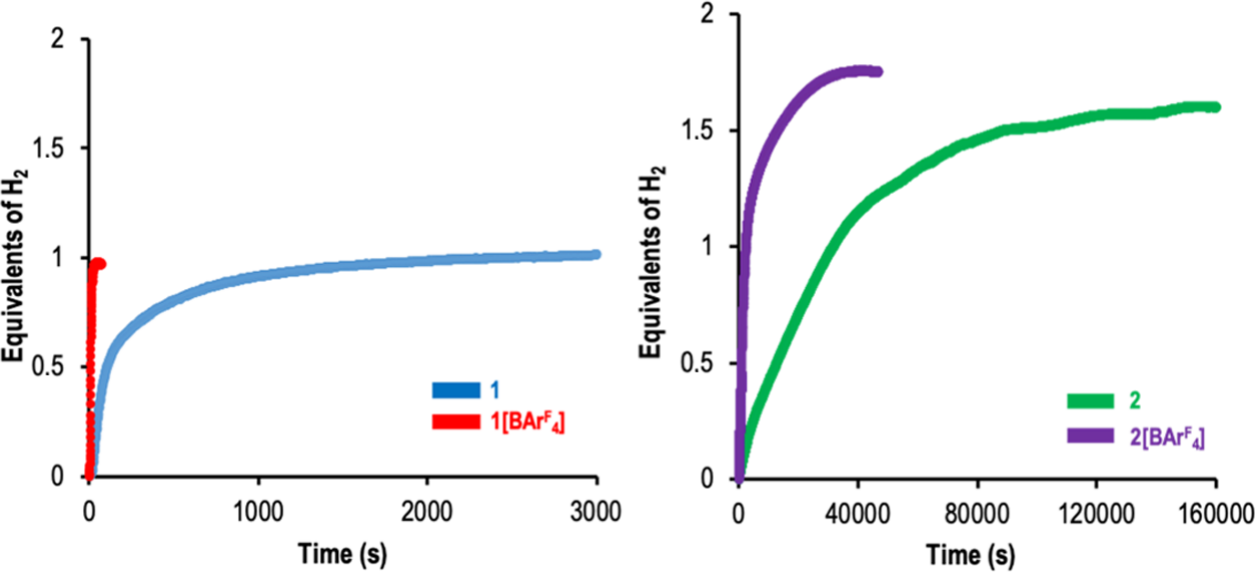
Reaction profiles (equiv of H_2_ generated vs time) for hydrolysis of diphenylsilane using **1** and a **1[BAr**^F^_4_**]** (left), **2** and **2[BAr**^F^_4_**]** (right) as precatalysts. Reaction conditions: Silane (0.22 mmol), H_2_O (2.2 mmol), 0.2 mol % of catalyst in 1 mL of THF at 25 °C under N_2_. The hydrogen production was calculated by continuous monitoring of the pressure evolution using a pressure transducer (Man on the Moon X102 kit).

Compounds, **1**, **2**, **1[BAr**^F^_4_**]**, and **2[BAr**^F^_4_**]** were tested as precatalysts for the hydrolysis of Ph_2_SiH_2_ in tetrahydrofuran (THF) under standard reaction conditions (0.2 mol % catalyst, [Ph_2_SiH_2_] = 0.22 M, 10 equiv H_2_O; see SI for more information). The H_2_ evolution was monitored through the Man on the Moon X102 kit. The reaction profiles obtained (equiv of H_2_ vs time) are shown in Figure 3.

It is worth highlighting the different reaction profiles obtained when comparing the cationic with the neutral compounds, which points to the integrity of the M–Cl bond through the catalytic process.

When Ir(III)-based catalysts **2** and **2[BAr**^F^_4_**]** were employed, more than 1.6 equiv of H_2_ were liberated in the process, being the product of the double hydrolysis, diphenylsilanediol (**3c** in Table 1, entries 2 and 4 and Figures S3 and S4 in SI), the only silane-containing product detected by ^1^H NMR at the end of the reaction. Moreover, the absence of Ph_2_SiH_2_ indicates that the reaction is complete. In this process, the cationic **2[BAr^F^_4_]** is more active than the neutral compound **2**. The reaction profiles observed, based on hydrogen evolution (Figure 3), did not show an evident two-stepped process, pointing to similar reaction rates for the first and second hydrolysis of the dihydrosilane.

**Table 1:**
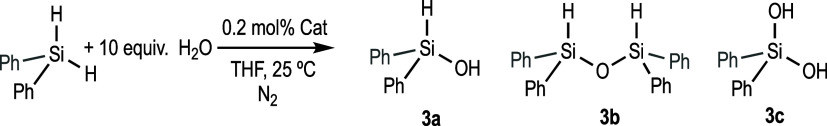
Catalytic Hydrolysis of Diphenylsilane^a^

entry	cat.	H_2_^b^ equiv	time (s)	**3a**^c^	**3b**^c^	**3c**^c^
1	**1**	1	2500	93	7	
2	**2**	1.6	155,500			>99
3	**1[BAr**^F^_4_**]**	1	50	1	99	
4	**2[BAr**^F^_4_**]**	1.75	38,460			>99

a Reaction conditions: Silane (0.22 mmol), H_2_O (2.2 mmol), 0.2 mol % of catalyst in 1 mL of THF at 25 °C.

b Hydrogen equivalents evolved.

c Molar ratio of products calculated by ^1^H NMR.

**Figure 4:**
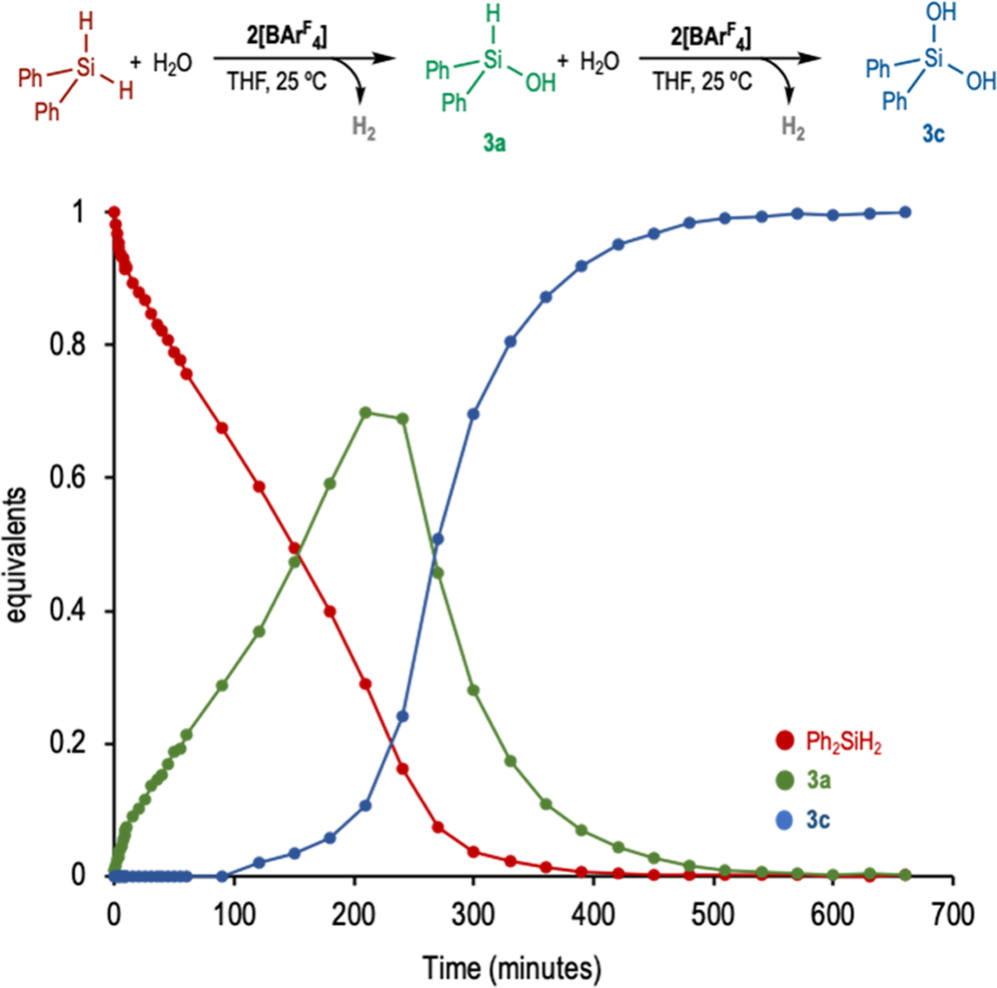
Time-correlated speciation diagram. Hydrolysis of Ph_2_SiH_2_ catalyzed by **2[BAr**^F^_4_**]**. Reaction conditions: Ph_2_SiH_2_ (0.11 mmol), H_2_O (1.1 mmol), **2[BAr**^F^_4_**]** 0.2 mol %, 0.5 mL THF-d_8_, 25 °C.

To confirm the sequential formation of **3c**, the hydrolysis of diphenylsilane catalyzed by **2[BAr**^F^_4_**]** was monitored by in situ ^1^H NMR spectroscopy (Figure S5 in SI). The time-correlated speciation diagram obtained (Figure 4) shows the sigmoidal formation of diphenylsilanediol (**3c**), typical of consecutive reactions, being diphenylhydrosilanol (**3a**) the reaction intermediate. It is worth mentioning that the resemblance of the first and second hydrolysis rate constants hampers the isolation of diphenylhydrosilanol **3a** with Ir(III)-based catalyst **2** and **2[BAr**_**4**_^**F**^**]**.

In contrast, when the reaction was catalyzed by rhodium(III)-based complexes **1** or **1[BAr**^F^_4_**]**, the reaction profile reached a plateau after liberating only 1 equiv of H_2_ (Figure 3). The cationic complex **1[BAr**^F^_4_**]** was the most efficient precatalyst, and the process was completed in only 50 seconds (Figure 3 and entry 3 in Table 1). When the neutral complex **1** was used as a precatalyst, 2500 s were required to reach the same conversion (Figure 3 and entry 1 in Table 1). The difference in reaction rate observed depending on the catalyst used (neutral or cationic) may be due to two reasons: (a) the easier accessibility of the substrate to the metal center in the case of the cationic complex; (b) the stronger electrophilicity of the cationic complexes with respect to the neutral ones.

**Scheme 4:**
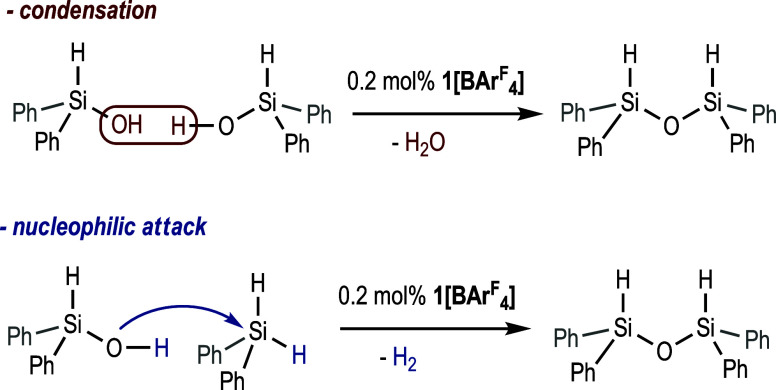
Possible Reaction Pathways for the Formation of Diphenyldihydrosiloxane Catalyzed by **1[BAr^F^_4_]**

Surprisingly, ^1^H NMR inspection of the reaction mixtures at the end of the reaction showed that the product obtained was mainly diphenylhydrosilanol (**3a**) when **1** was used as a catalyst (Table 1, entry 1 and Figure S1 in SI), but it proceeded toward tetraphenyldihydrosiloxane (**3b**) with the cationic catalyst **1[BAr**^F^_4_**]** (Table 1, entry 3 and Figure S2 in SI). Two reaction pathways can be envisaged for the formation of dihydrosiloxane **3b** under the reaction conditions: the condensation of two molecules of diphenylhydrosilanol (**3a**) (liberating one molecule of H_2_O) or the nucleophilic attack of a diphenylhydrosilanol (**3a**) to a diphenyldihydrosilane (evolving 1 equiv of H_2_) (Scheme 4).

**Scheme 5:**
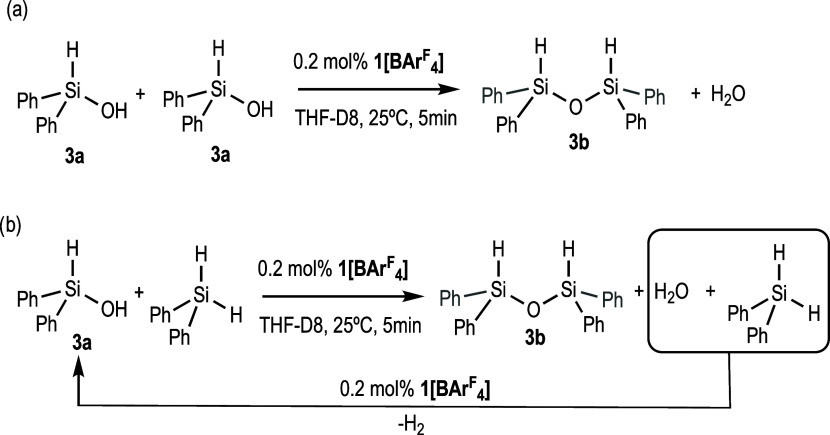
Control Experiments for Diphenyldihydrosiloxane Formation

To shed some light on the origin of the observed dihydrosiloxane when using **1[BAr**^F^_4_**]**, two control experiments were performed by means of ^1^H NMR. First, diphenylhydrosilanol **3a** (0.11 mmol) was treated with 0.2 mol % of **1[BAr**^F^_4_**]** in THF-d_8_ (0.5 mL) at 25 °C. The ^1^H NMR spectrum acquired after 5 min of reaction shows nearly complete consumption of **3a** and the clear formation of the dihydrosiloxane **3b** (Scheme 5a, Figure S6a in SI). When an equimolecular mixture of diphenylhydrosilanol **3a** (0.11 mmol) and Ph_2_SiH_2_ (0.11 mmol) was reacted under identical conditions, the ^1^H NMR spectra acquired after 5 min showed, as in the previous experiment, the formation of the dihydrosiloxane **3b** and the total consumption of **3a**. Noticeably, Ph_2_SiH_2_ was only partially (≈50%) consumed, which supports the hypothesis that **3b** is formed by a fast condensation of the formed diphenylhydrosilanol **3a**. The partial consumption of Ph_2_SiH_2_ must be attributed to its **1[BAr**^F^_4_**]**-catalyzed hydrolysis due to the H_2_O formed during the condensation reaction. Accordingly, a small signal assigned to the H_2_ generated in this process was observed in the ^1^H NMR spectra (Scheme 5b and Figure S6b in SI).

The results obtained show that whereas both neutral and cationic rhodium complexes **1** and **1[BAr**^F^_4_**]** are very effective catalysts for the first hydrolysis of Ph_2_SiH_2_, in the case of **1[BAr**^F^_4_**]**, the product of the reaction **3a** is immediately involved in a metal-mediated condensation process. Noticeably, this condensation reaction is not observed in the case of **1** at short reaction times. These results suggest the need of two coordination vacancies in the metal center, or a highly electrophilic metal center, for the condensation to proceed at competitive rates.

**Scheme 6:**
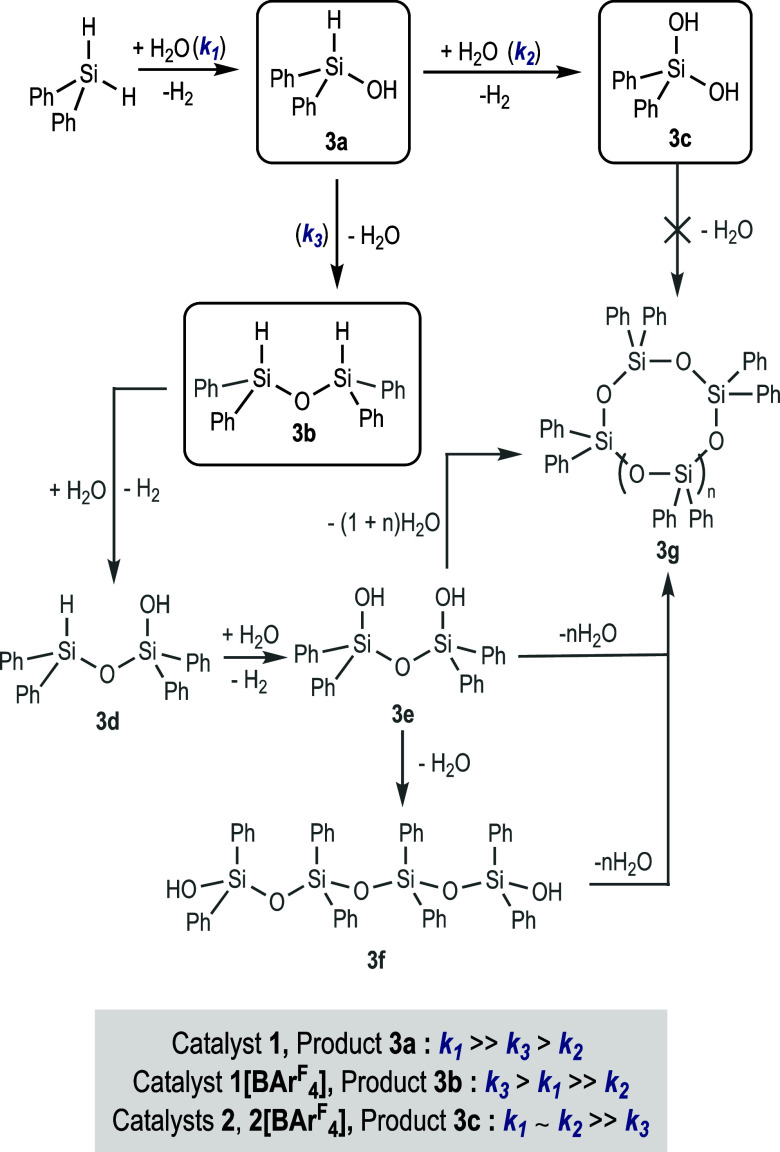
Hydrolysis of Diphenylsilane: Network of Potential Coexisting Reactions

A global analysis of the results obtained with the four catalytic systems studied reveals the network of coexisting reactions in the hydrolysis of diphenylsilane (Scheme 6).^27^ The different reaction rates for each individual step observed when compared to the different catalysts studied allowed us to selectively obtain the product of monohydrolysis (**3a**), its condensation product (**3b**), or the doubly hydrolyzed silane (**3c**). No other silane-containing products were detected by ^1^H NMR in the reaction mixtures when iridium-based catalysts were used in the process, which discards the formation of polysiloxanes with these systems.

**Scheme 7:**
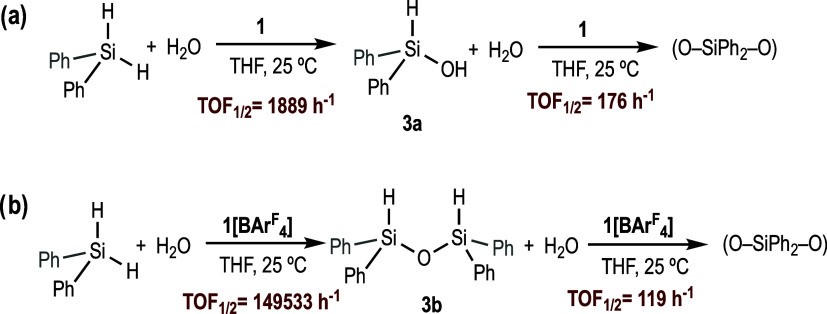
Hydrolysis of Diphenylsilane Catalyzed by **1** (a) and **1[BAr^F^_4_]** (b) TOFs_1/2_ Calculated in Red

To confirm whether compound **1** at extended reaction times would catalyze the second hydrolysis or the condensation reaction, a catalytic reaction was conducted with this compound for 24 h. The reaction profile showed that after the initial formation of **3a**, a second equivalent of hydrogen evolved but at a much lower rate (Scheme 7 and Figure S7 in SI). ^1^H NMR analysis of the final reaction mixture shows that the reaction product is not the diphenylsilanediol (**3c**), but a complex mixture of (O–SiPh_2_–O) species (**3e** is proposed as one of these species, Figure S9 in SI). To obtain more information, the same reaction was monitored by in situ ^1^H NMR spectroscopy (Figure S11 in SI). This experiment shows that after Ph_2_SiH_2_ is consumed, **3a** begins to transform into dihydrosiloxane **3b**, which subsequently transforms into another product containing at least one Si–H unit (probably **3d**). At longer reaction times, these signals evolve towards (O–SiPh_2_–O) species. Similar results were obtained when the hydrolysis of Ph_2_SiH_2_ was conducted for 24 h with catalysts **1[BAr**^F^_4_**]** (Figures S8 and S10 in SI). Taken together, these results suggest that most probably, **1** follows a condensation route (*via***3b**) rather than the double hydrolysis of the dihydrosilane (*via***3c**) (*k*_3_ > *k*_2_).

### Hydrolysis of Other Secondary Silanes

The fine balance between rate constants observed when catalysts **1**, **1[BAr**^F^_4_**]**, **2**, and **2[BAr**^F^_4_**]** were studied in the hydrolysis of Ph_2_SiH_2_ and the special relevance of hydrosilanols and hydrosiloxanes as valuable synthons for the chemical industry prompted us to extend the study of the rhodium-based catalysts to other commercial secondary silanes (entries 1–8 in Table 2).

**Table 2:**
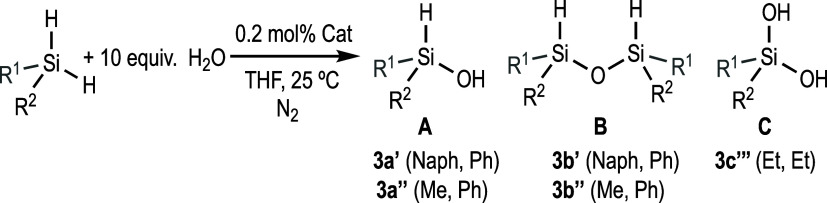
Catalytic Hydrolysis of Dihydrosilanes^a^

entry	cat.	R^1^, R^2^	H_2_^b^ equiv	TOF_1/2_ (h^–1^)	**A**^c^	**B**^c^	**C**^c^
1	**1**	Ph, Ph	1	1889	93	7	
2	**1**	Ph, Naph	1	78	90	10	
3	**1**	Ph, Me	1	2991	10	90	
4	**1**	Et, Et	0.93	6452	unidentified		
5	**1[BAr**^F^_4_**]**	Ph, Ph	1	149,533	1	99	
6	**1[BAr^F^_4_]**	Ph, Naph	1	13,962	97	3	
7	**1[BAr**^F^_4_**]**	Ph, Me	1	472,861	2	98	
8	**1[BAr**^F^_4_**]**	Et, Et	1.8	390,087^d^			99

a Reaction conditions: Silane (0.22 mmol), H_2_O (2.2 mmol), 0.2 mol % of catalyst in 1 mL of THF at 25 °C under N_2_.

b Hydrogen equivalents released.

c Molar ratio of products calculated by ^1^H NMR.

d TOF_1/2_ has been calculated for the release of 1 equiv of hydrogen.

The reaction of 1-naphtyl(phenyl)dihydrosilane (1-Naph(Ph)SiH_2_) with H_2_O in the presence of catalytic amounts of the rhodium complexes **1** or **1[BAr**^F^_4_**]** led to the formation of a mixture of hydrosilanol **3a′** and dihydrosiloxane **3b′** (entries 2 and 6 in Table 2), being the hydrosilanol **3a′** the main product (selectivity > 90%) in both cases. Accordingly, in both reactions, only 1 equiv of H_2_ was released. As with Ph_2_SiH_2_, the reaction catalyzed by complex **1** was slower than the reaction catalyzed by **1[BAr**^F^_4_**]**.

The results obtained in the hydrolysis of methyl(phenyl)dihydrosilane (entries 3 and 7 of Table 2) show a change in the reactivity, obtaining the respective dihydrosiloxane **3b″** as a main product indistinctly when **1** or **1[BAr**^F^_4_**]** were used as precatalysts (selectivity > 90%). As with Ph_2_SiH_2_ and 1-Naph(Ph)SiH_2_, these reactions only released 1 equiv of H_2_ and the hydrolysis performed using the cationic rhodium complex is faster.

Finally, diethyldihydrosilane (Et_2_SiH_2_) was tested (entries 4 and 8 in Table 2). The hydrolysis of Et_2_SiH_2_ using **1** as a precatalyst releases 1 equiv of H_2_, but the products formed could not be identified. Surprisingly, using this substrate and **1[BAr**^F^_4_**]** as a precatalyst, almost 2 equiv of H_2_ were released, albeit at clearly different rates. The first equivalent was liberated in less than 1 min, and 25 min sufficed to reach completion. The product observed at the end of the reaction was diethylsilanediol (**3c‴**), presumably formed by hydrolysis of diethylhydrosilanol (**3a‴**).

The results obtained with the different silanes are compatible with the reaction scheme shown above (Scheme 6). Once the hydrosilanol is formed, its fate will depend on the relative rates of second hydrolysis (*k*_2_) and condensation (*k*_3_) reactions. If both are slow, the product could be isolated (as is the case with Ph_2_SiH_2_ and to a certain extent with 1-Naph(Ph)SiH_2_). If the second hydrolysis is fast, the product will evolve to the corresponding silanediol (**C** in Table 2). The relative rates for the hydrolysis can be inferred from the calculated turnover frequency (TOFs) based on the liberation of 1 equiv of H_2_. According to these values, this reaction is approximately 100 times faster using the cationic rhodium complex **1[BAr**^F^_4_**]** than when neutral **1** was used, with all the substrates. As discussed above, this enhanced reactivity toward the hydrolysis could be explained by the stronger electrophilicity and/or the better accessibility of the substrate to the catalytic pocket when the cationic catalyst is used. The relative reaction rates observed for the different substrates (Et_2_SiH_2_ > MePhSiH_2_ > Ph_2_SiH_2_ > 1-Naph(Ph)SiH_2_) support the important influence of electronic factors in the activation barrier for this process. This electronic effect could be extrapolated to the hydrolysis of the formed hydrosilanol. Consistently, only in the case of Et_2_SiH_2_, the diethylsilanediol (**3c‴**) was obtained. It is worth reminding that highly electrophilic Ir(III) catalyst **2** and **2[BAr**^F^_4_**]** were required to obtain the corresponding silanediol (**3c**) from Ph_2_SiH_2_.

Alternatively, if the second hydrolysis is slow compared to the condensation reaction, the hydrosilanol could be isolated or evolve toward the dihydrosiloxane if involved in a fast metal-catalyzed condensation. According to the results obtained, steric effects determine the rate of the condensation process. When using the largest silane, 1-Naph(Ph)SiH_2_, the major product obtained is unreacted hydrosilanol, independently of the catalyst used. In contrast, the product obtained when using Me(Ph)SiH_2_ is the dihydrosiloxane.

In view of the excellent catalytic properties of our system, we decided to study the catalytic behavior of complex **1[BAr**^F^_4_**]** in the alcoholysis of Ph_2_SiH_2_ and the hydrolysis of other silanes (see Section S9 in SI). Satisfyingly, the alcoholysis of Ph_2_SiH_2_ with MeOH, EtOH, and iPrOH rendered the corresponding alkoxyhydrosilanes. When primary silanes were subjected to hydrolysis, the corresponding siloxanes were obtained as the main product, whereas tertiary silanes rendered an unidentified mixture of products liberating 2 equiv of H_2_.

### Scale-Up Experiments

It should be noted that, to the best of our knowledge, few efficient methods for the selective synthesis of hydrosilanols have been reported to date.^24^

**Scheme 8:**
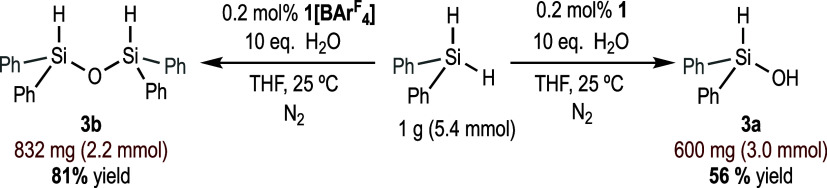
Gram-Scale Synthesis of Diphenylhydrosilanol and Diphenyldihydrosiloxane

To prove that using precataysts **1** and **1[BAr**^F^_4_**]**, it is possible to obtain selectively the diphenylhydrosilanol (**3a**) and diphenyldihydrosiloxane (**3b**) in a gram-scale, the reaction of 5.4 mmol (1 g) of Ph_2_SiH_2_ with 10 equiv of H_2_O under standard catalytic conditions (0.2 mol % of **1** or **1[BAr**^F^_4_**]** as catalyst, THF as solvent) was performed. When precatalyst **1** was used, this reaction led to the formation of diphenylhydrosilanol (**3a**) in a 56% of isolated yield (600 mg). Using **1[BAr**^F^_4_**]** as a precatalyst, diphenyldihydrosiloxane **3b** was obtained in 81% of isolated yield (832 mg) (Scheme 8).

The robustness of the catalytic system derived from **1[BAr**^F^_4_**]** was also confirmed through successive additions of Ph_2_SiH_2_. The sequential reaction profiles obtained showed that the catalyst maintained its activity for, at least, 10 successive cycles (Figure S12 in SI). In addition, in the case of the neutral compounds **1** and **2**, their structures remain unchanged after the catalytic reaction (Figures S1 and S3 in SI), which also demonstrate the robustness of the catalytic system.

## Conclusions

In summary, two neutral {MCl[SiMe_2_(*o*-C_6_H_4_PPh_2_)]_2_} (M = Rh, **1**; Ir, **2**) and two cationic {M[SiMe_2_(*o*-C_6_H_4_PPh_2_)]_2_(NCMe)_n_}[BAr^F^_4_] (M = Rh and *n* = 1, **1[BAr**^F^_4_**]**; M = Ir and *n* = 2, **2[BAr**^F^_4_**]**) complexes were prepared and structurally characterized in solution by NMR and in the solid state by X-ray crystallography. The four new complexes proved to be catalytically active in the hydrolysis of dihydrosilanes, rendering different products depending on the catalyst/substrate combination. These air-stable complexes have shown excellent catalytic properties with low catalyst loadings at room temperature and without the requirement of additives. A rational analysis of the different products obtained allowed us to propose a network of coexisting metal-catalyzed reactions of different nature (hydrolysis of hydrosilanes, condensation of silanols, and nucleophilic attack of silanols on hydrosilanes). Apparently, the latter are not operative with our systems. The results showed that hydrolytic processes are mainly controlled by the electronic nature of both catalysts and substrates, whereas metal-catalyzed condensations are mostly affected by sterics. This subtle balance permitted us to obtain selectively hydrosilanols, silanediols, or dihydrosilanes by an educated selection of the catalyst and substrate. The practical application of this methodology has been demonstrated by gram-scale synthesis experiments using Ph_2_SiH_2_.

## Experimental Section

### General Considerations

The preparation of the metal complexes was carried out at room temperature under nitrogen by standard Schlenk techniques. Glassware was oven dried at 120 °C overnight and flamed under a vacuum prior to use. CH_2_Cl_2_ and THF were distilled over CaH_2_ and Na, respectively, degassed by successive freeze–pump–thaw cycles and stored over molecular sieves (3 Å). [RhCl(coe)_2_]^28^ and [IrCl(coe)_2_]^29^ complexes and the proligand *o*-Ph_2_P(C_6_H_4_)SiMe_2_H^30^ were prepared as previously reported. Microanalyses were carried out with a Leco CHNS-932 microanalyzer. NMR spectra were recorded with Bruker Avance DPX 300, Bruker Avance 400, or Bruker Avance 500 spectrometers at room temperature unless otherwise stated; ^1^H and ^13^C{^1^H} (residual solvents) and ^31^P{^1^H} (H_3_PO_4_ external standard) spectra were measured from CDCl_3_, CD_2_Cl_2_, THF-d_8_ solutions. IR spectra were recorded with a Nicolet FTIR 510 spectrophotometer using KBr pellets.

#### Synthesis of {MCl[SiMe_2_(*o*-C_6_H_4_PPh_2_)]_2_} (M = Rh, **1**; M = Ir, **2**)

[M(coe)_2_Cl]_2_ (M = Rh, Ir) (0.14 mmol) was solved in CH_2_Cl_2_ (4 mL), 4 equiv of *o*-Ph_2_P(C_6_H_4_)SiMe_2_H (180 mg, 0.560 mmol) were added, and it was stirred for 20 min. After this time, solvent was removed under the vacuum, and the resulting solid was washed with 5 mL of *n*-pentane and 5 mL of methanol and dried under vacuum to obtain a pale-yellow (**1**) and yellow (**2**) solids. Yield of **1** 164 mg (76%). Yield of **2** 201 mg (83%).

##### **1**

^1^H NMR (400 MHz, CD_2_Cl_2_): δ 8.10–7.10 (Si(*o*-C_6_H_4_PPh_2_), 28 H_arom_), −0.07 (s, Si–CH_3_, 6H), −0.43 (s, Si–CH_3_, 6H). ^31^P{^1^H} NMR (202 MHz CD_2_Cl_2_): δ 55.6 (d, *J*_Rh-P_ = 120 Hz, 2P). ^13^C{^1^H} NMR (101 MHz, CD_2_Cl_2_): δ 160.0–128.0 (36 C_arom._, Si(*o*-C_6_H_4_PPh_2_)), 7.3 (s, 2C, Si–CH_3_), 2.4 (s, 2C, Si–CH_3_). Microanalysis (RhClSi_2_P_2_C_40_H_40_·CH_2_Cl_2_): Requires: C 57.12, H 4.91. Obtained: C 56.95, H 5.02.

##### **2**

^1^H NMR (400 MHz, CD_2_Cl_2_): δ 8.02–7.25 (Si(*o*-C_6_H_4_PPh_2_), 28 H_arom_), −0.11 (s, Si–CH_3_, 6H), −0.51 (s, Si–CH_3_, 6H). ^31^P{^1^H} NMR (202 MHz, CD_2_Cl_2_): δ 54.4 (s, 2P). ^13^C{^1^H} NMR (101 MHz, CD_2_Cl_2_): δ 159.6–126.5 (36 C_arom_, Si(*o*-C_6_H_4_PPh_2_)), 5.5 (s, 2C, Si–CH_3_), −0.3 (s, 2C, Si–CH_3_). Microanalysis (IrClSi_2_P_2_C_40_H_40_): Requires: C 55.44, H 4.65. Obtained: C 55.55, H 4.58.

#### Synthesis of {Rh[SiMe_2_(*o*-C_6_H_4_PPh_2_)]_2_(NCMe)}[BAr^F^_4_] (**1[BAr^F^_4_]**) and {Ir[SiMe_2_(*o*-C_6_H_4_PPh_2_)]_2_(NCMe)_2_}[BAr^F^_4_] (**2[BAr^F^_4_]**)

To a Schlenk charged with the [M(SiMe_2_(*o*-C_6_H_4_PPh_2_))_2_Cl] (M = Rh, Ir) complex (**1**, 100 mg, 0.13 mmol; **2**, 112 mg, 0.13 mmol) and NaBAr^F^_4_ (127 mg, 0.14 mmol), 4 mL of CH_2_Cl_2_ was added. After 20 min stirring, the yellow suspension formed was filtered via cannula to remove NaCl. Addition of 1 mL of MeCN gave a pale-yellow solution, and it was left stirring 10 min. After this time, solvent was removed under vacuum to give a pale-yellow solid in both cases. Yield of **1[BAr**^F^_4_**]** 184 mg (86%). Yield of **2[BAr**^F^_4_**]** 194 mg (84%).

##### **1[BAr^F^_4_]**

^1^H NMR (400 MHz, CDCl_3_): δ 7.73 (m, 8H, BAr^F^_4_), 7.55 (m, 4H, BAr^F^_4_), 7.70–7.32 (Si(*o*-C_6_H_4_PPh_2_), 28 H_arom_), 1.64 (s, CH_3_–CN, 3H), 0.04 (s, Si–CH_3_, 6H), −0.32 (s, Si–CH_3_, 6H). ^31^P{^1^H} NMR (202 MHz, CDCl_3_): δ 55.5 (d, *J*_Rh-P_ = 120 Hz, 2P). ^13^C{^1^H} NMR (101 MHz, CDCl_3_): δ 161.8 (q, *J*_B-C_ = 50 Hz, BAr^F^_4_), 134.9 (s, BAr^F^_4_), 129.0 (q, *J*_F-C_ = 12 Hz, BAr^F^_4_), 124.7 (q, *J*_F-C_ = 273 Hz, CF_3_), 122.4 (s, 2C, NC–CH_3_), 117.6 (m, BAr_4_^F^), 159.0–128.0 (36 C_arom_, Si(*o*-C_6_H_4_PPh_2_)), 7.5 (s, 2C, Si–CH_3_), 2.6 (s, 2C, Si–CH_3_), 1.8 (s, 1C, NC–CH_3_). Microanalysis (RhSi_2_P_2_C_74_H_55_BNF_24_): Required: C 54.00, H 3.37, N 0.85. Obtained: C 53.88, H 3.55, N 1.02.

##### **2[BAr^F^_4_]**

^1^H NMR (400 MHz, CDCl_3_): δ 7.71 (s, 8H, BAr^F^_4_), 7.53 (s, 4H, BAr^F^_4_), 7.75–7.70 (Si(*o*-C_6_H_4_PPh_2_), 28 H_arom_), 1.48 (s, CH_3_–CN, 3H), −0.10 (s, Si–CH_3_, 6H), −0.26 (s, Si–CH_3_, 6H). ^31^P{^1^H} NMR (202 MHz, CDCl_3_): δ 38.1 (s, 2P). ^13^C{^1^H} NMR (101 MHz, CDCl_3_): δ 161.8 (q, *J*_B-C_ = 50 Hz, BAr^F^_4_), 134.9 (s, BAr^F^_4_), 129.0 (q, *J*_F-C_ = 12 Hz, BAr_4_^F^), 124.7 (q, *J*_F-C_ = 273 Hz, CF_3_), 119.3 (s, 2C, NC–CH_3_), 117.6 (m, BAr_4_^F^), 160.0–127.0 (36 C_arom_, Si(*o*-C_6_H_4_PPh_2_)), 4.4 (s, 2C, Si–CH_3_), 1.8 (s, 2C, NC–CH_3_), 0.9 (s, 2C, Si–CH_3_). Microanalysis (IrSi_2_P_2_C_76_H_58_BN_2_F_24_): Requires: C 51.39, H 3.29 N 1.58. Obtained: C 51.02, H 3.39, N 1.29.

### X-ray Crystallography

Crystals for **1**, **1[BAr**^F^_4_**]**, **2**, and **2[BAr**^F^_4_**]** were obtained by diffusion of pentane over CH_2_Cl_2_ and were mounted on a glass fiber and used for data collection on a Bruker Apex II with a photon detector equipped with graphite monochromated Mo Kα radiation (λ = 0.71073 Å). Lorentz-polarization and empirical absorption corrections were applied. The structures were solved by direct methods and refined with full-matrix least-squares calculations on *F*^2^ using the program SHELXT.^31^ Anisotropic temperature factors were assigned to all atoms except for hydrogen atoms, which are riding their parent atoms with an isotropic temperature factor arbitrarily chosen as 1.2 times that of the respective parent. Final *R*(*F*), w*R*(F2), and goodness-of-fit agreement factors, details on the data collection, and analysis can be found in Table S1.

### General Procedure for Catalytic Hydrolysis of Silanes

A closed reaction vessel equipped with a pressure transducer (Manonthemoon kinetic kit X102)^32^ was immersed in a thermostated ethylene glycol/water bath and charged with the catalyst (0.00044 mmol) in 1 mL of distilled THF and H_2_O (2.2 mmol). Once the pressure of the system was stabilized, the silane (0.22 mmol) was added, which was considered initial reaction time. The solution was left stirring until the pressure stabilized again, which was indicative that the reaction ended. The quantity of gas evolved was calculated from the measured pressure inside the reaction vessel following the ideal gases law equation (reactor volume 13.2 mL). Then, solvent was removed under vacuum, and the reaction mixture was analyzed by ^1^H NMR to determine the molar ratio of products. The major reaction product was isolated through purification by column chromatography on silica gel using Hexane/EtOAc (10:1) as eluents.

#### Ph_2_Si(H)OH, Diphenylsilanol (**3a**)

^1^H NMR (400 MHz, CDCl_3_): δ 7.68–7.63 (m, 4H_arom_), 7.48–7.39 (m, 6H_arom_), 5.53 (s, 1H, Si–H), 2.59 (s, 1H, Si–OH). ^13^C{^1^H} NMR (126 MHz, CDCl_3_): δ 135.3 (2C), 134.3 (4C), 130.7 (2C), 128.4 (4C) ppm. HMQC (^1^H–^29^Si) NMR (400 MHz, CDCl_3_): δ (^29^Si) −12.9. IR (KBr): 3209 cm^–1^ (broad, Si–O–H), 2125 (Si–H) cm^–1^.

#### Ph_2_(H)SiOSi(H)Ph_2_, Tetraphenyldisiloxane (**3b**)

^1^H NMR (400 MHz, CDCl_3_): δ 7.60–7.55 (m, 8H_arom_), 7.45–7.40 (m, 4H_arom_), 7.39–7.33 (m, 8H_arom_), 5.62 (s, 2H, Si–H). ^13^C{^1^H} NMR (126 MHz, CDCl_3_): δ 135.3 (4C), 134.7 (8C), 130.6 (4C), 128.3 (8C) ppm. HMQC (^1^H–^29^Si) NMR (400 MHz, CDCl_3_): δ (^29^Si) −19.5 ppm. Ir (KBr): 2123 cm^–1^ (Si–H), 1087 (Si–O–Si) cm^–1^.

#### Ph_2_Si(OH)_2_, Diphenylsilanediol (**3c**)

^1^H NMR (400 MHz, THF-d_8_): δ 7.68–7.64 (m, 4H_arom_), 7.32–7.23 (m, 6H_arom_), 6.00 (s, 2H, Si–OH). ^13^C{^1^H} NMR (126 MHz, THF-d_8_): δ 138.8 (2C), 135.2 (4C), 129.9 (2C), 128.0 (4C). HMQC (^1^H–^29^Si) NMR (400 MHz, THF-d_8_): δ (^29^Si) −33.3 ppm. IR (KBr): 3197 (broad, Si–O–H) cm^–1^.

#### 1-NaphPhSi(H)OH, 1-Naphtyl(phenyl)silanol (**3a′**)

^1^H NMR (400 MHz, CDCl_3_): δ 8.17 (dm, *J* = 7.9 Hz, 1H_arom_), 7.97 (dm, *J* = 8.3 Hz, 1H_arom_), 7.89 (tm, *J* = 7.5 Hz, 2H_arom_), 7.68 (dm, *J* = 7.9 Hz, 2H_arom_), 7.54–7.42 (m, 4H_arom_), 7.39 (dm, *J* = 7.5 Hz, 2H_arom_), 5.90 (s, 1H, Si–H), 2.80 (s, 1H, Si–OH). ^13^C{^1^H} NMR (126 MHz, CDCl_3_): δ 137.1 (1C), 135.5 (1C), 135.4 (1C), 134.6 (2C), 133.6 (1C), 133.1 (1C), 131.5 (1C), 130.7 (1C), 129.2 (1C), 128.4 (2C), 128.1 (1C), 126.7 (1C), 126.1 (1C), 125.5 (1C). HMQC (^1^H–^29^Si) NMR (400 MHz, CDCl_3_): δ (29Si) −13.2. IR (KBr): 3295 cm^–1^ (broad, Si–O–H), 2136 (Si–H) cm^–1^.

#### 1-NaphPh(H)SiOSi(H)Ph(1-Naph), Di-1-naphtyl(diphenyl)disiloxane (**3b′**)

^1^H NMR (400 MHz, CDCl_3_): δ 8.06 (dd, *J* = 8.4 Hz, *J* = 4.0, 2H_arom_), 7.95 (dd, *J* = 8.4 Hz, *J* = 4.0 Hz, 2H_arom_), 7.87 (dd, *J* = 8.4 Hz, *J* = 3.2 Hz, 2H_arom_), 7.84 (tm, *J* = 7.6 Hz, 2H_arom_), 7.60 (tm, *J* = 7.6 Hz, 4H_arom_), 7.49–7.40 (m, 6H_arom_), 7.36–7.26 (m, 6H_arom_), 6.00 (s, 2H, Si–H). ^13^C{^1^H} NMR (126 MHz, CDCl_3_): δ 137.0 (2C), 135.6 (2C), 135.5 (2C), 134.5 (4C), 133.5 (2C), 133.0 (2C), 131.4 (2C), 130.5 (2C), 129.0 (2C), 128.3 (6C), 126.4 (2C), 126.0 (2C), 125.4 (2C). HMQC (^1^H–^29^Si) NMR (400 MHz, CDCl_3_): δ (^29^Si) −18.3 ppm. IR (KBr): 2129 cm^–1^ (Si–H), 1056 (Si–O–Si) cm^–1^.

#### MePh(H)SiOSi(H)PhMe, Dimethyl(diphenyl)disiloxane (**3b″**)

^1^H NMR (400 MHz, CDCl_3_): δ 7.61–7.57 (m, 4H_arom_), 7.44–7.37 (m, 6H_arom_), 5.18 (m, 2H, Si–H), 0.47 (d, *J* = 2.82 Hz, 6H, Si–CH_3_). ^13^C{^1^H} NMR (126 MHz, CDCl_3_): δ 137.4 (2C), 133.7 (4C), 130.2 (2C), 128.2 (4C), −0.3 (s, 2C, Si–CH_3_). HMQC (^1^H–^29^Si) NMR (400 MHz, CDCl_3_): δ (^29^Si) −11.6. IR (KBr): 2132 cm^–1^ (Si–H), 1062 cm^–1^ (Si–O–Si).

#### Et_2_Si(OH)_2_, Diethylsilanediol (**3c‴**)

^1^H NMR (400 MHz, THF-d_8_): δ 4.96 (s, 2H, Si–OH), 0.93 (t, *J* = 7.9 Hz, 6H, CH_3_) 0.45 (q, *J* = 7.9 Hz, 4H, CH_2_). ^13^C{^1^H} NMR (126 MHz, THF-d_8_): δ 7.5 (2C), 7.1 (2C). HMQC (^1^H–^29^Si) NMR (400 MHz, THF-d_8_): δ (^29^Si) −7.0. IR (KBr): 3162 cm^–1^ (broad, Si–O–H).

## Supporting Information Available

Additional experimental details, NMR and FTRI spectra, and X-ray crystallographic tables (PDF)
