# Supplementary material for: Tailor-Made Synthesis of Hydrosilanols, Hydrosiloxanes, and Silanediols Catalyzed by di-Silyl Rhodium(III) and Iridium(III) Complexes
Source: Inorg Chem. 2023 Feb 9;62(7):3095–105. doi: 10.1021/acs.inorgchem.2c03953 (PMC10863934; doi:10.1021/acs.inorgchem.2c03953)
Supplement: Supplementary file 1 — ic2c03953_si_001.pdf [file ic2c03953_si_001.pdf]

Supporting Information

Tailor-Made Synthesis of Hydrosilanols, Hydrosiloxanes and Silanediols Catalyzed by di-Silyl Rhodium(III) and Iridium(III) Complexes

Unai Prieto,† Antonio Rodriguez-Dieguez,§ Zoraida Freixa*,†,¶ and Miguel A. Huertos*,†,¶

† Facultad de Química, Universidad del País Vasco (UPV/EHU), 20018, San Sebastián, Spain

§ Departamento de Química Inorgánica, Universidad de Granada, 18071, Granada, Spain

¶ IKERBASQUE, Basque Foundation for Science, 48011, Bilbao, Spain

Email: zoraida_freixa@ehu.es; miguelangel.huertos@ehu.es

Table of Contents

1. Catalytic hydrolysis of diphenylsilane S-2
2. NMR kinetics of hydrolysis of diphenylsilane catalyzed by **2[BAr$^F{}_4$]** S-5
3. Control experiments to elucidate the formation of diphenylsiloxane S-6
4. Hydrolysis of diphenylsilane using **1** and **1[BAr$^F{}_4$]** at long reaction times S-7
5. Reciclability of **1[BAr$^F{}_4$]** S-10
6. Gram-scale synthesis of diphenylsilanol and diphenylsiloxane S-10
7. Hydrolysis of other dihydrosilanes catalyzed by **1** and **1[BAr$^F{}_4$]** S-11
8. Network of potential coexisting reactions involved in the formation of silanols and siloxanes S-17
9. Hydrolysis and alcoholysis of other silanes S-18
10. X-ray crystallographic tables S-19
11. NMR and FTIR spectra S-20

1. Catalytic hydrolysis of diphenylsilane

Reaction Conditions: An initial set of experiments were conducted to optimize catalyst and water concentrations with constant amounts of diphenylsilane (0.22 mmol) and solvent (THF, 1mL).

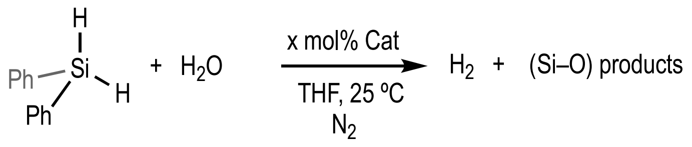

Table S.1. Optimization of the catalyst and water concentration in the hydrolysis of diphenylsilane

entry	Cat.	mol % Cat	Equiv. H_2O	H_2 equiv.	$TOF_{1/2}$ (h^{-1})
1	**1**	0.2	10	1	1889
2	**1**	0.8	10	1	4146
3	**1**	1	10	1	7068
4	**1**	0.2	1	0.92	108
5	**1**	0.2	5	1	404
6	**1[BAr$^F_4$]**	0.2	10	1	149533
7	**1[BAr$^F_4$]**	0.2	1	1	91200
8	**2**	0.2	10	1.6	57
9	**2[BAr$^F_4$]**	0.2	10	1.75	758

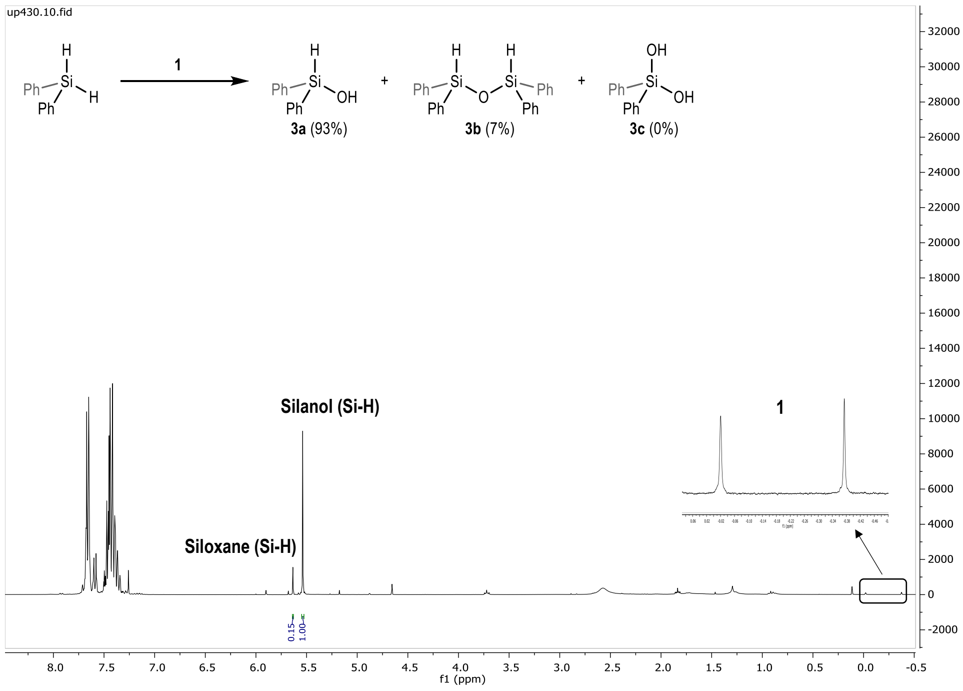

Figure S.1. ^{1}H NMR of hydrolysis of diphenylsilane catalyzed by **1**.

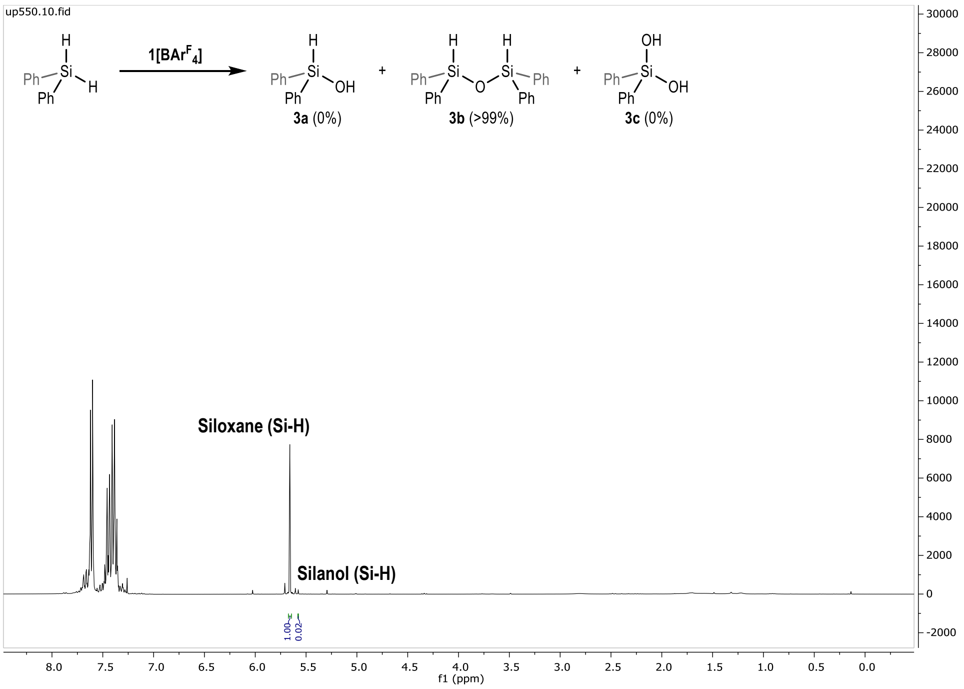

Figure S.2. ^{1}H NMR of hydrolysis of diphenylsilane catalyzed by **1[BAr$^F_4$]**.

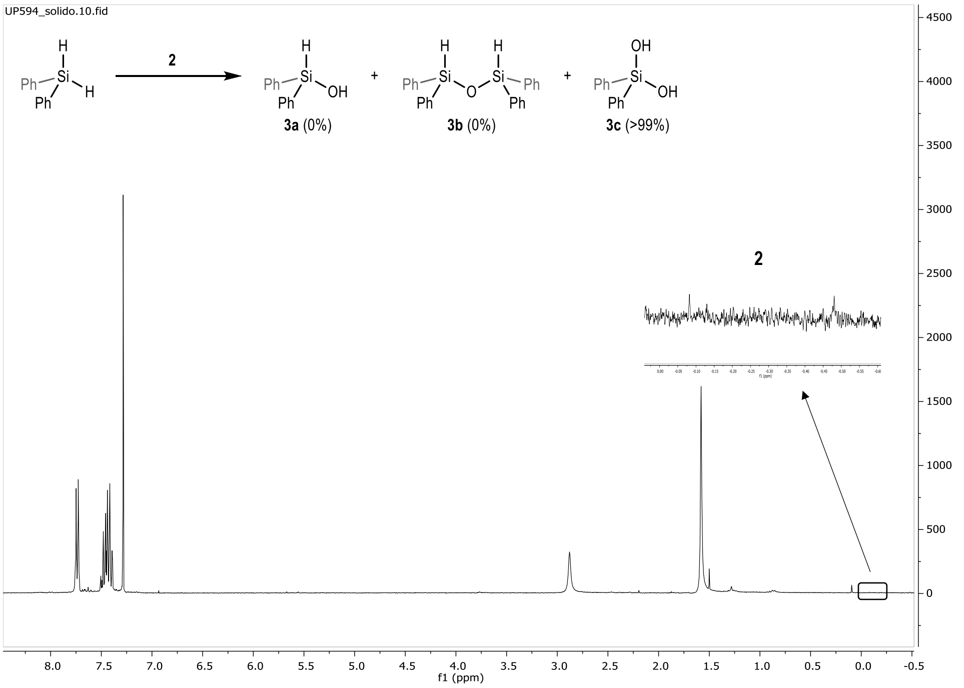

Figure S.3. ^{1}H NMR of hydrolysis of diphenylsilane catalyzed by **2**.

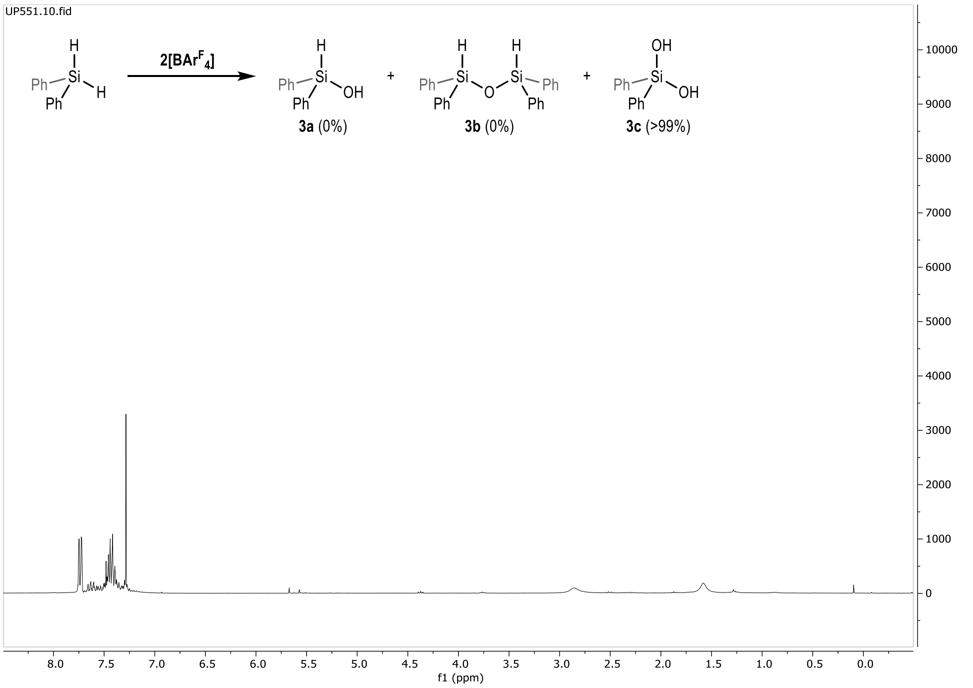

Figure S.4. ^{1}H NMR of hydrolysis of diphenylsilane catalyzed by **2[BArF$_4$]**.

2. NMR kinetics of hydrolysis of diphenylsilane catalyzed by 2[BAr^F$_4$].

A high-pressure Young NMR tube was charged with diphenylsilane (0.11 mmol), **2[BAr^F$_4$]**. (0.2 mol%), H_2O (1.1 mmol) and 0.5 mL of THF-d^8. ^{1}H NMR spectra were recorded at regular time intervals.

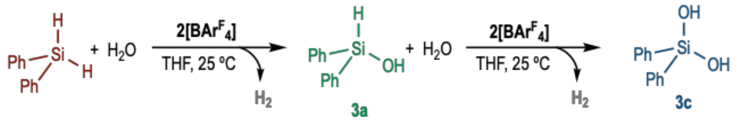

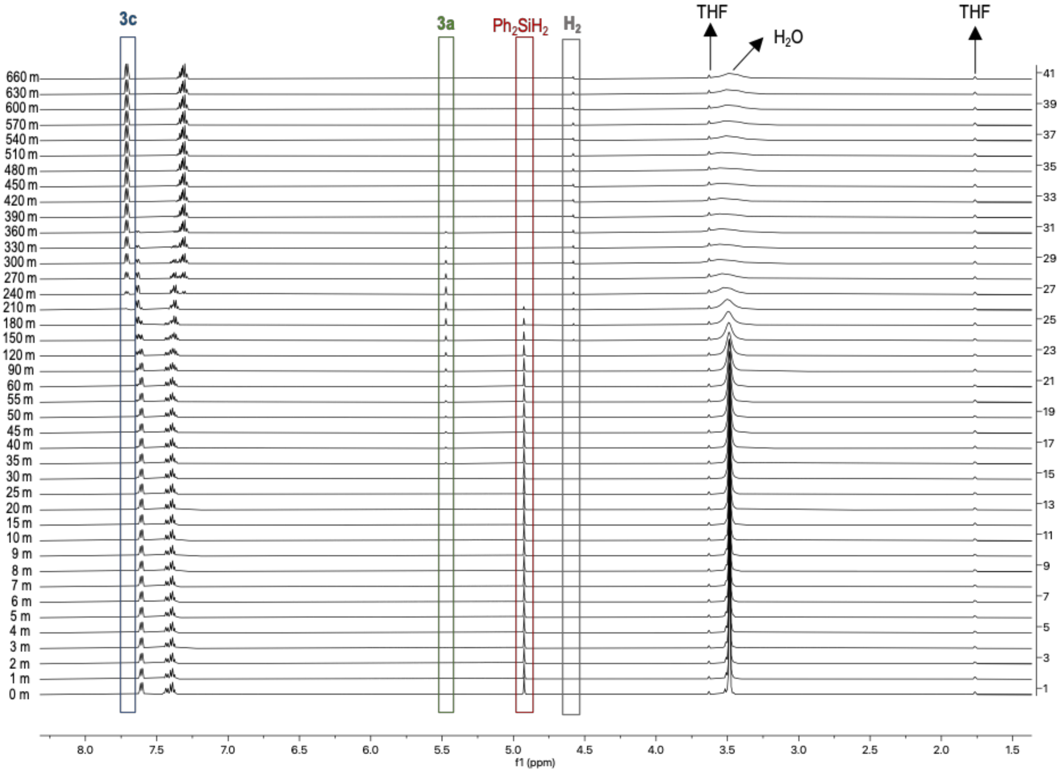

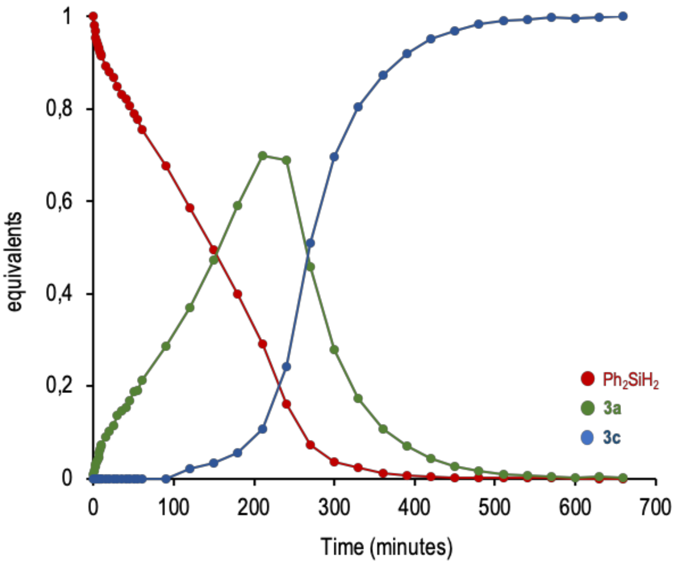

Figure S.5. ^{1}H NMR array of the reaction of dihenylsilane with water in the presence of 0.2 mol% of **2[BAr^F$_4$]** and time-correlated speciation diagram.

3. Control experiments to elucidate the formation of diphenylsiloxane.

a)

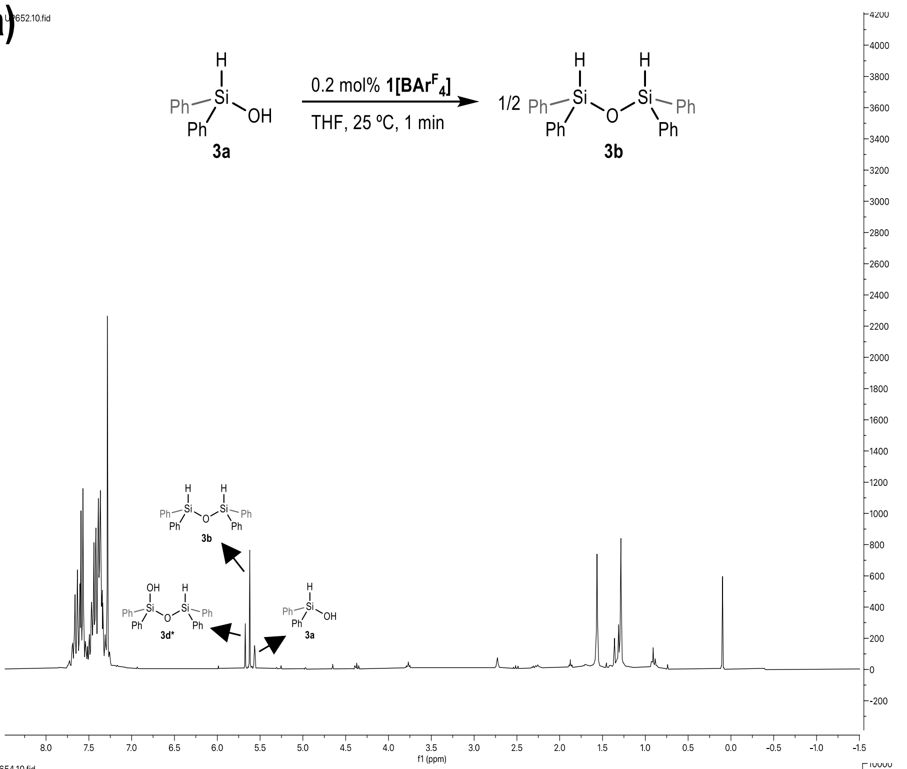

b)

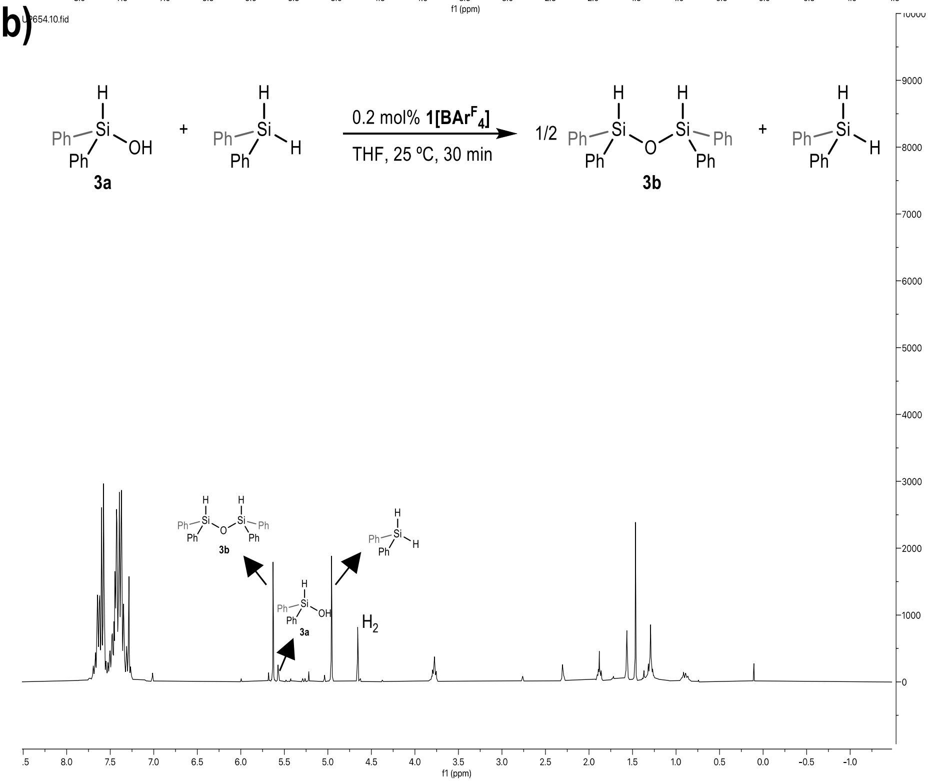

Figure S.6. a) ^{1}H NMR spectrum of the reaction of hydrosilanol **3a** in THF in presence of 0.2 mol% of **1[BAr^F$_4$]**. **3d***, proposed compound. b) ^{1}H NMR spectrum of the reaction of hydrosilanol **3a** with diphenylsilane in THF in presence of 0.2 mol% of **1[BAr^F$_4$]**.

4. Hydrolysis of diphenylsilane using 1 and 1[BAr$^F_4$] at long reaction times

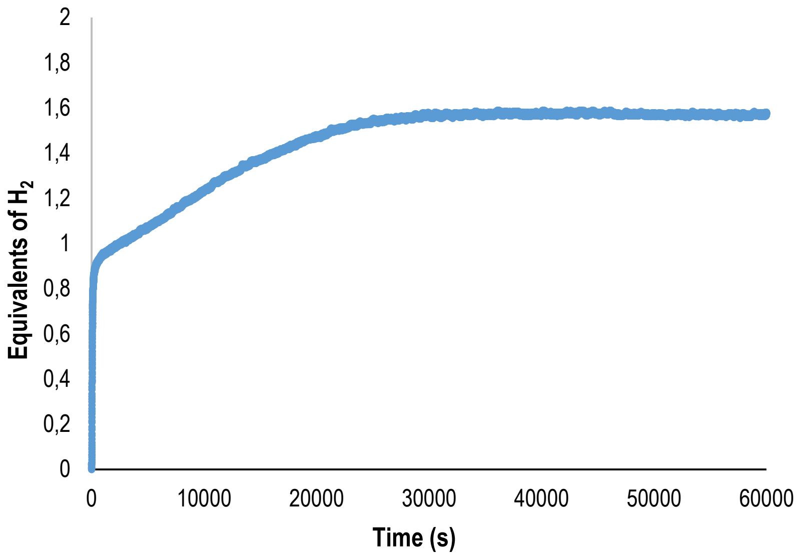

Figure S.7. Hydrolysis of diphenylsilane with **1** at extended reaction times.

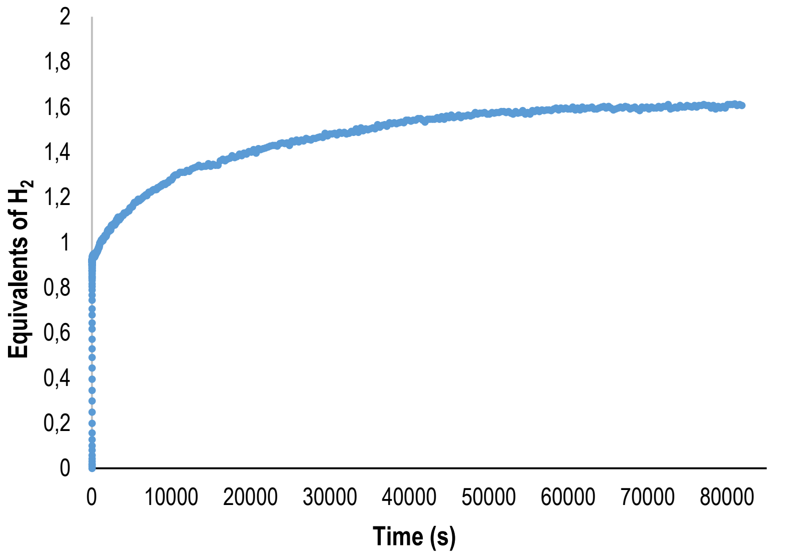

Figure S.8. Hydrolysis of diphenylsilane with **1[BAr$^F_4$]** at extended reaction times.

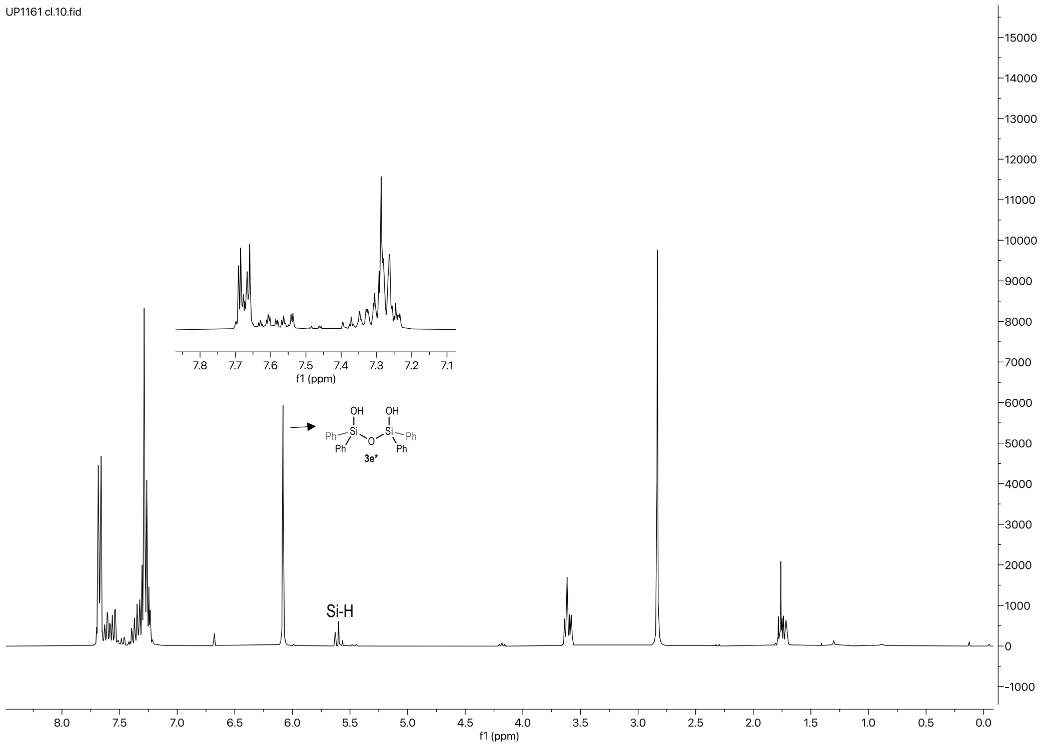

Figure S.9. ^{1}H NMR (THF-D8) of hydrolysis of diphenylsilane catalyzed by **1** after 24 hours of reaction. Reaction performed in the Man on the Moon X102 kit. Reaction conditions: Silane (0.22 mmol), H_2O (2.2 mmol), 0.2 mol % of catalyst in 1 mL of THF at 25 °C. **3e***, proposed compound.

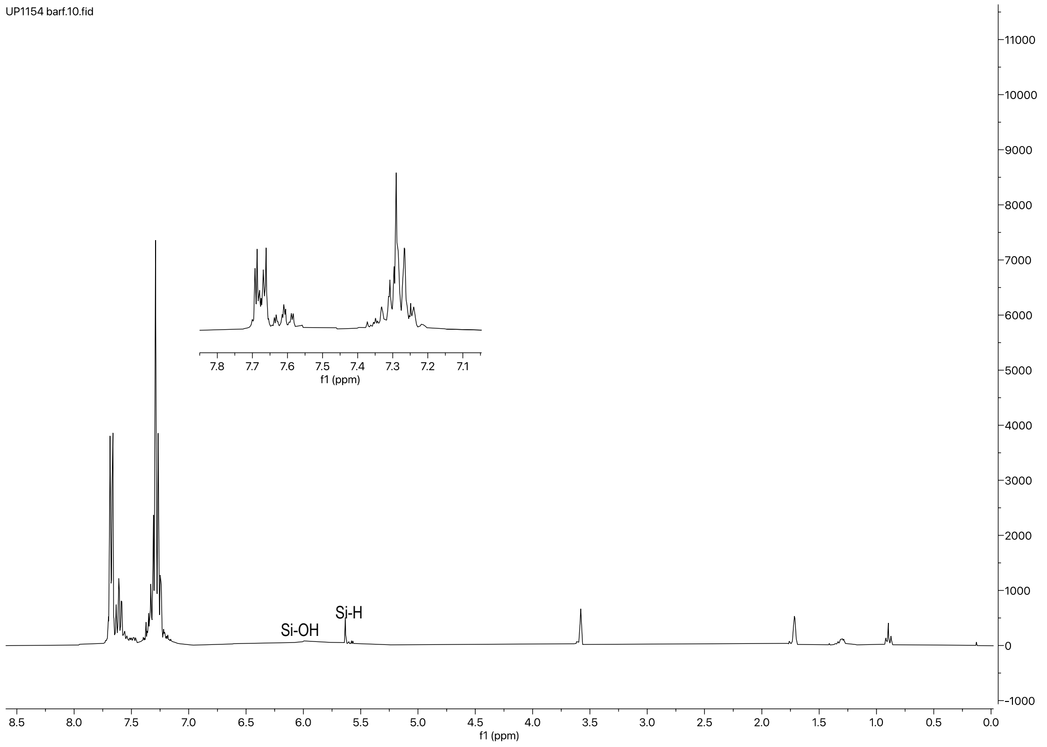

Figure S.10. ^{1}H NMR (THF-D8) of hydrolysis of diphenylsilane catalyzed by **1[BAr^F$_4$]** after 24 hours of reaction. Reaction performed in the Man on the Moon X102 kit. Reaction conditions: Silane (0.22 mmol), H_2O (2.2 mmol), 0.2 mol % of catalyst in 1 mL of THF at 25 °C.

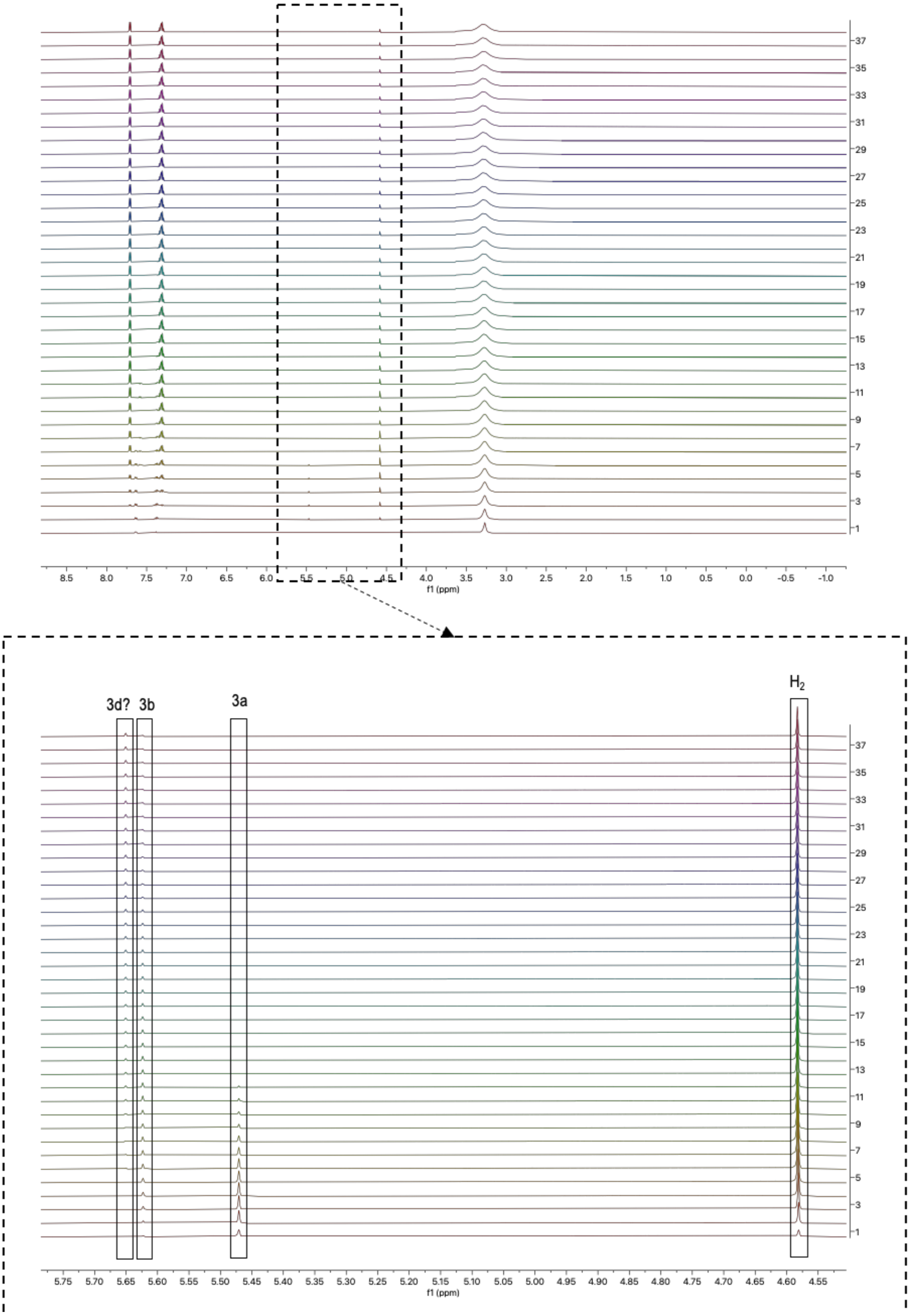

Figure S.11. ^{1}H NMR (THF-D8) monitoring of hydrolysis of diphenylsilane catalyzed by **1**. ^{1}H NMR collected every 30 minutes. Reaction performed in a High-Pressure NMR tube. Reaction conditions: Silane (0.11 mmol), H_2O (1.1 mmol), 0.1 mol % of catalyst in 0.5 mL of THF-D8 at 25 °C.

5. Recyclability of 1[BAr$^F_4$]

The recyclability of **1[BAr$^F_4$]** was studied using Man on the Moon system, measuring H_2 gas pressure. In an inert atmosphere of nitrogen, 0.2 mol% of catalyst and 100 equivalents of distilled water were solved in 1 mL of distilled THF. 10 reaction cycles were done by sequentially adding 40 µL of diphenylsilane and restoring the initial pressure.

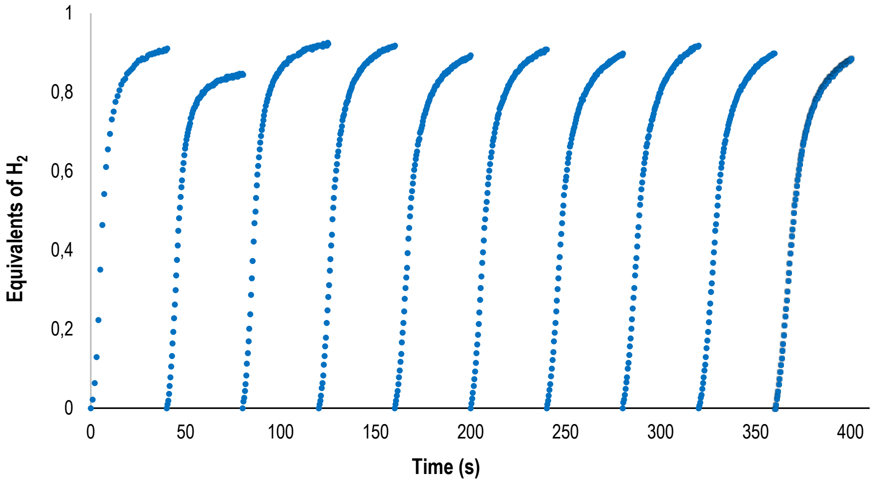

Figure S.12. Recyclability of **1[BAr$^F_4$]** using Ph_2SiH_2.

6. Gram-scale synthesis of diphenylhydrosilanol and tetraphenyldihydrosiloxane

Under nitrogen atmosphere, in a Schenk, 0.2 mol% of **1** or **1[BAr$^F_4$]** (0.01 mmol) and 10 equivalents of distilled water (0.97 mL, 54 mmol) were solved in 10 mL of distilled THF. Then a gram of diphenylsilane (5.4 mmol) was added. The reaction was stirred at room temperature for 40 minutes. Solvent was removed under vacuum. The resulting colorless oil was purified by silica-gel chromatography (10 Hex. / 1 AcOEt). Isolated yields: Reaction with **1**: Diphenylhydrosilanol (600 mg, 56 %), Reaction with **1[BAr$^F_4$]**: 1,1,3,3-Tetraphenyldihydrodisiloxane (832 mg, 81 %)

7. Hydrolysis of other dihydrosilanes catalyzed by 1 and 1[BAr$^F{}_4$].

Hydrolysis of naphthylphenylsilane

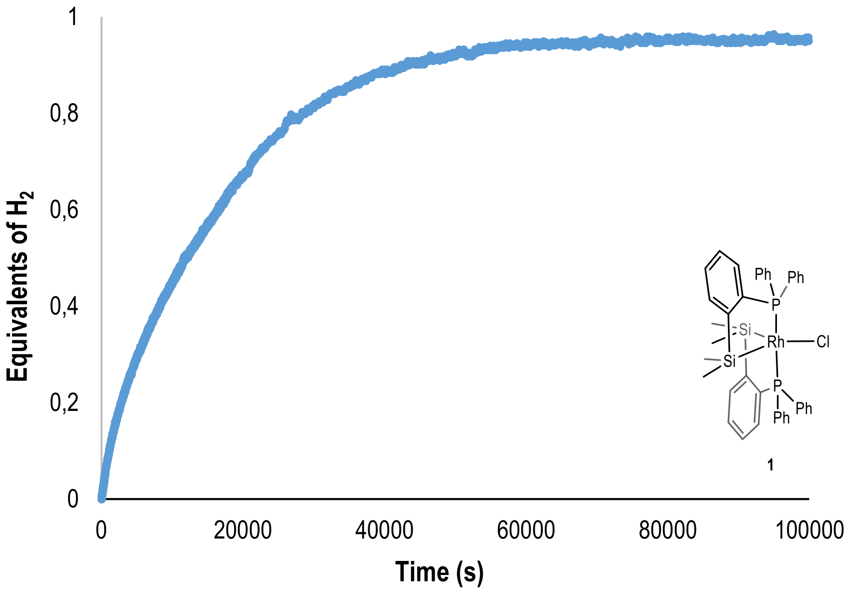

Figure S.13. Hydrolysis of naphthylphenylsilane catalyzed by **1**.

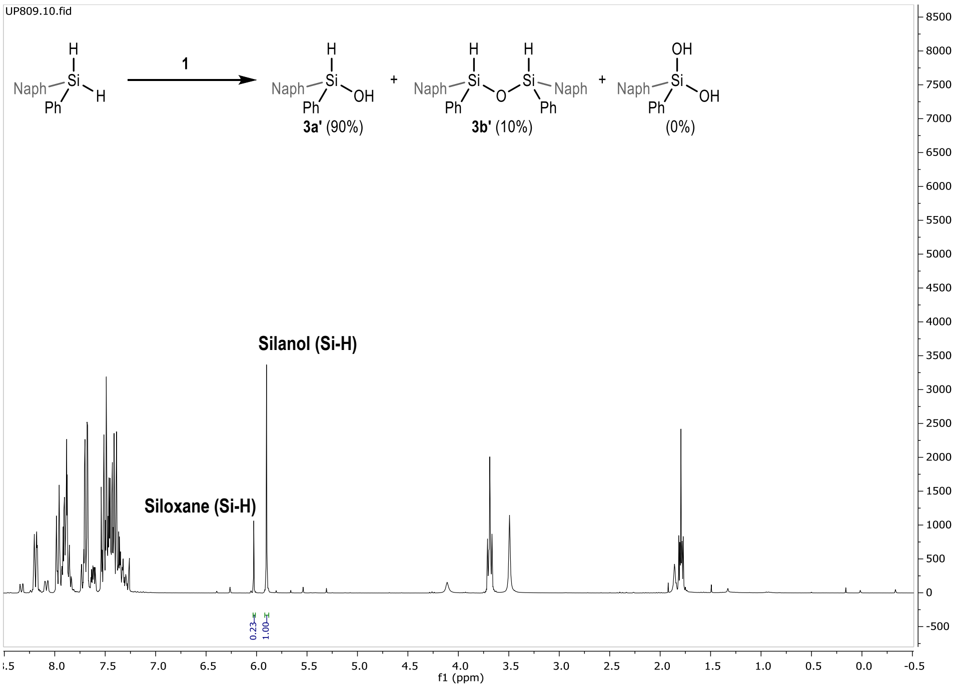

Figure S.14. ^{1}H NMR of hydrolysis of naphthylphenylsilane catalyzed by **1**.

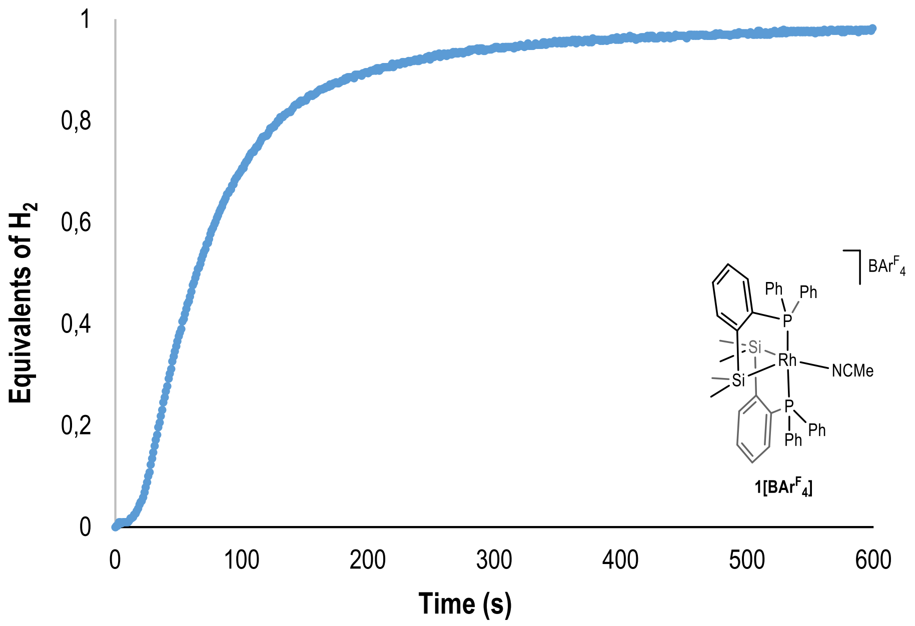

Figure S.15. Hydrolysis of naphthylphenylsilane catalyzed by **1[BArF4]**.

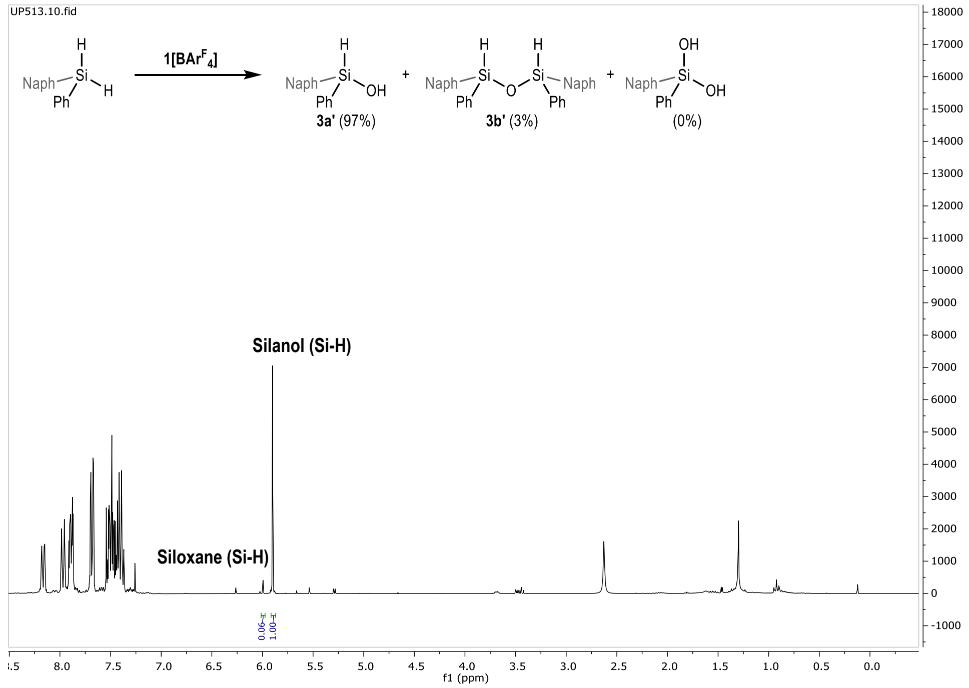

Figure S.16. ^{1}H NMR of hydrolysis of naphthylphenylsilane catalyzed by **1[BArF4]**.

<u>Hydrolysis of methylphenylsilane</u>

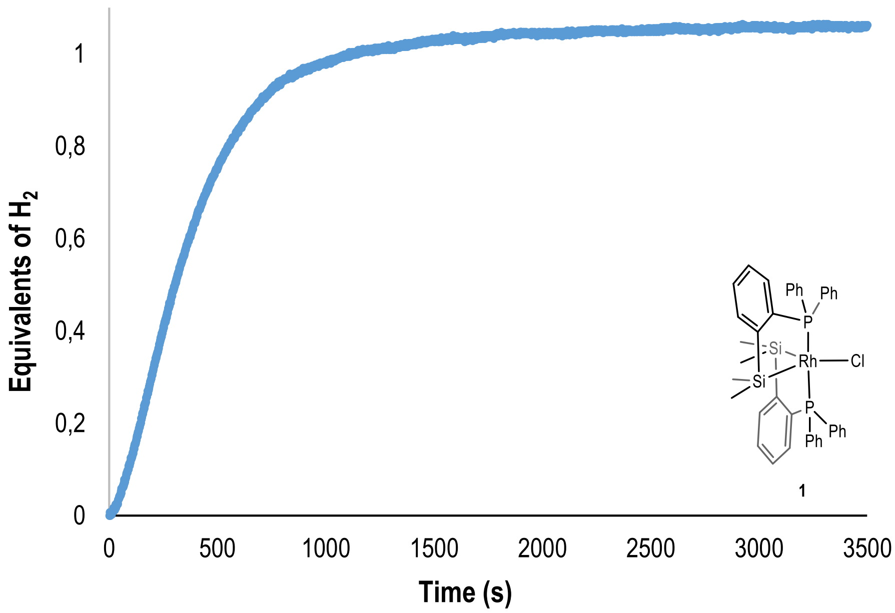

Figure S.17. Hydrolysis of methylphenylsilane catalyzed by **1**.

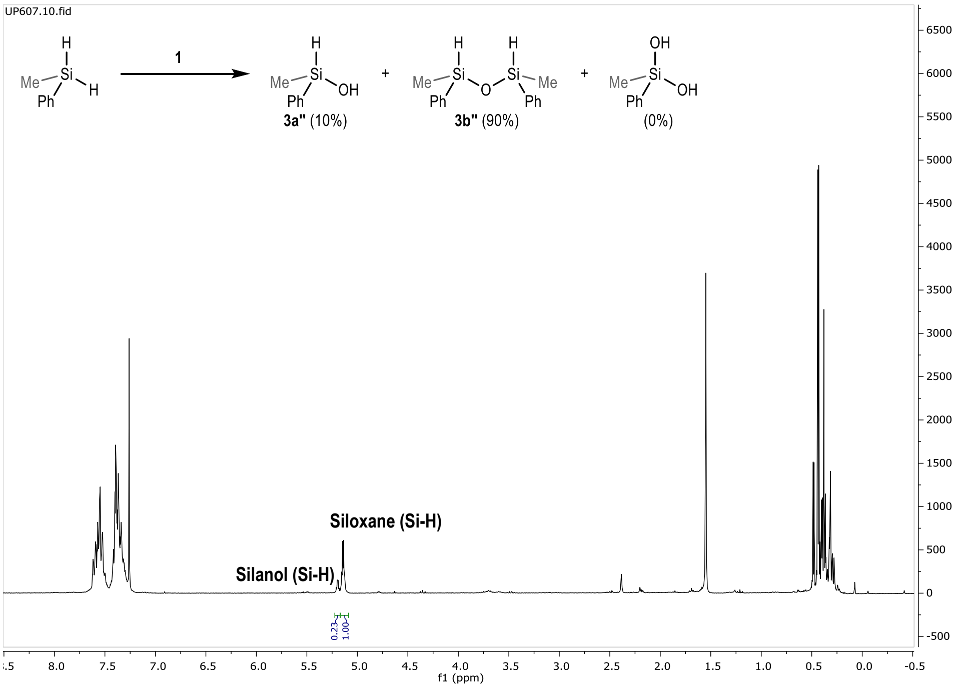

Figure S.18. ^{1}H NMR of hydrolysis of methylphenylsilane catalyzed by **1**.

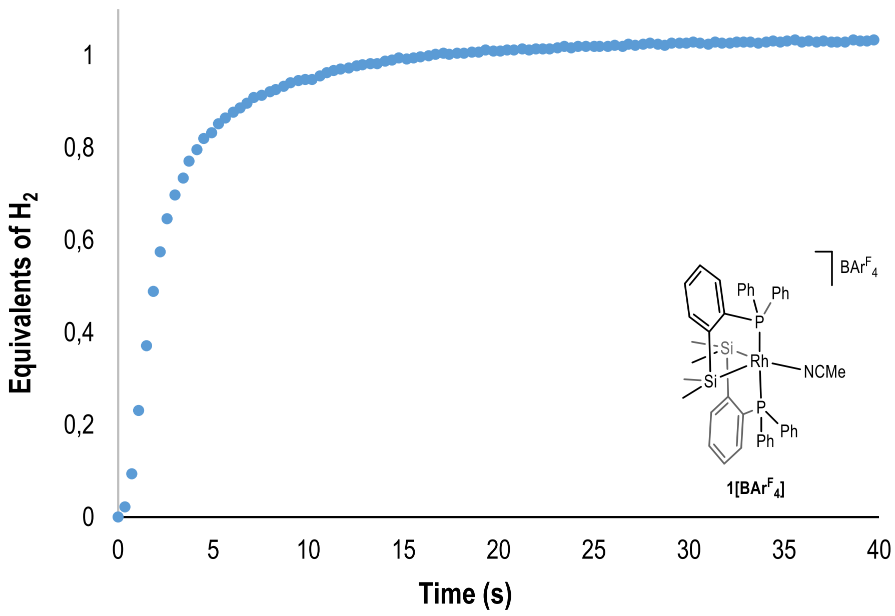

Figure S.19. Hydrolysis of methylphenylsilane catalyzed by **1[BAr$^F{}_4$]**.

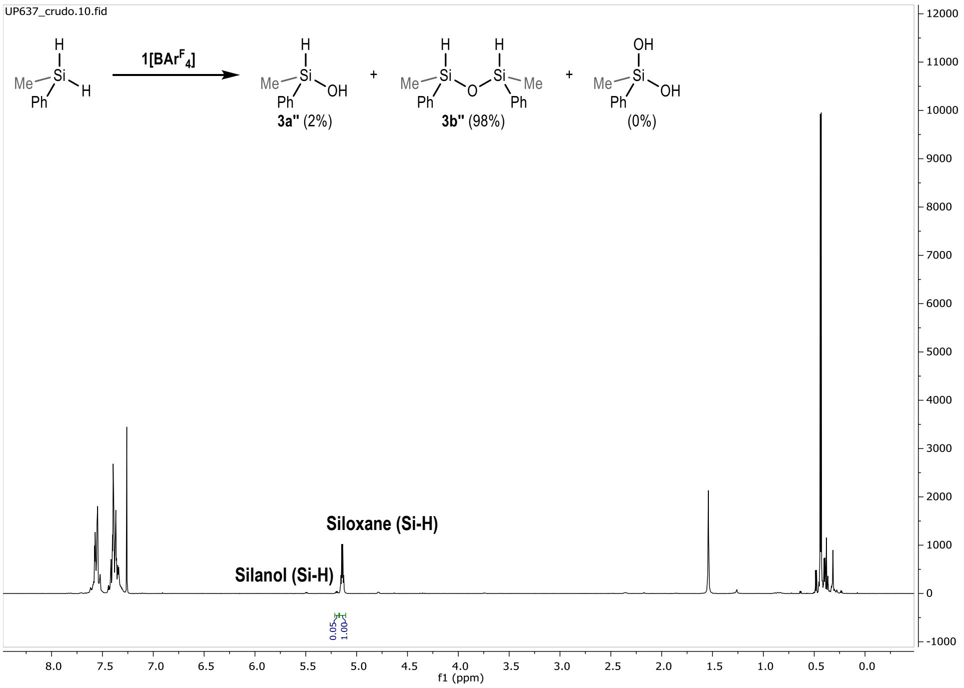

Figure S.20. ^{1}H NMR of hydrolysis of methylphenylsilane catalyzed by **1[BAr$^F{}_4$]**.

Hydrolysis of diethylsilane

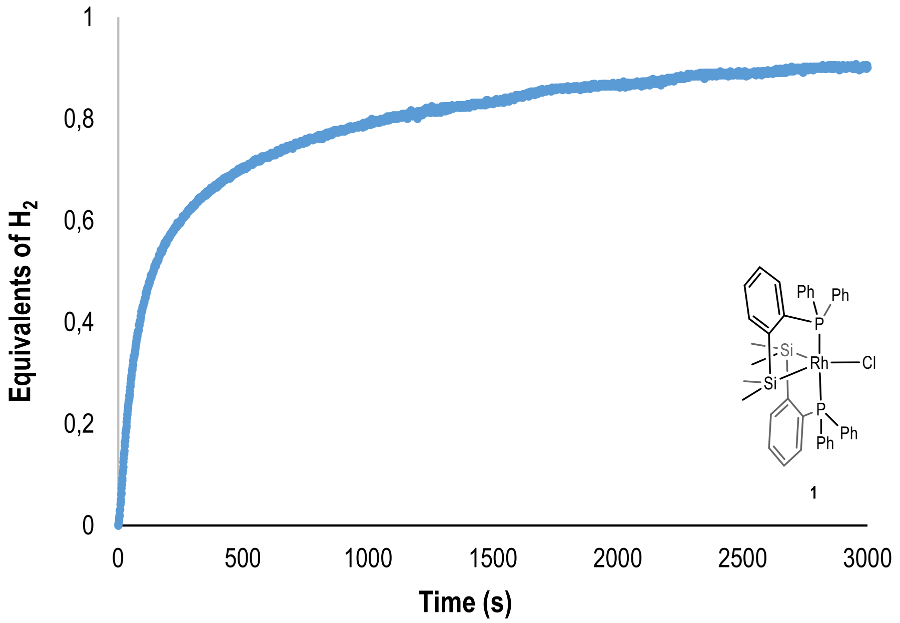

Figure S.21. Hydrolysis of diethylsilane catalyzed by **1**.

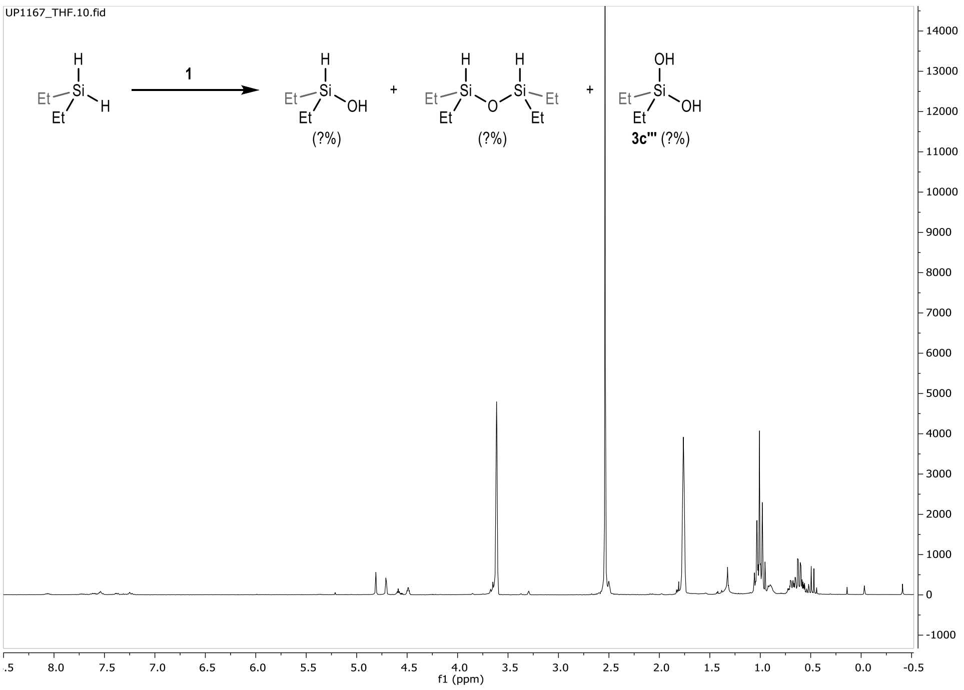

Figure S.22. ^{1}H NMR of hydrolysis of diethylsilane catalyzed by **1**.

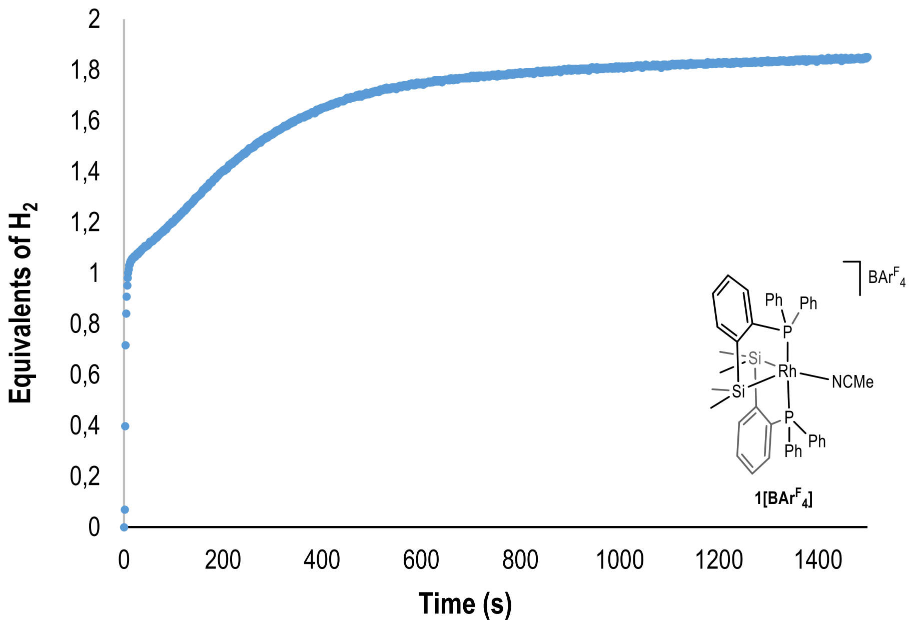

Figure S.23. Hydrolysis of diethylsilane catalyzed by **1[BAr$^F{}_4$]**.

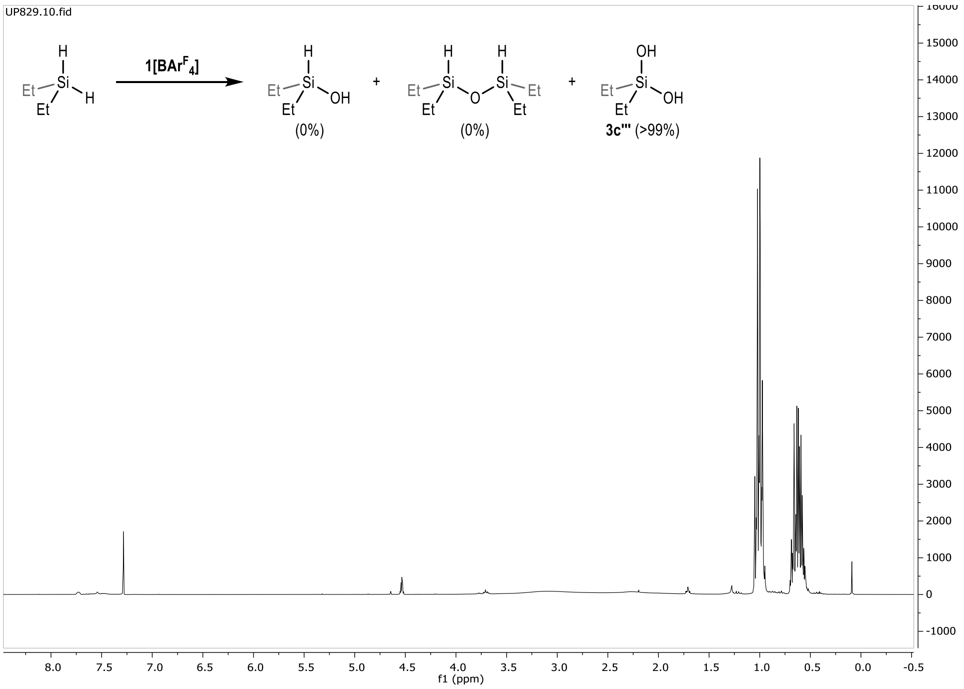

Figure S.24. ^{1}H NMR hydrolysis of diethylsilane catalyzed by **1[BAr$^F{}_4$]**.

8. Network of potential coexisting reactions involved in the formation of silanols and siloxanes.

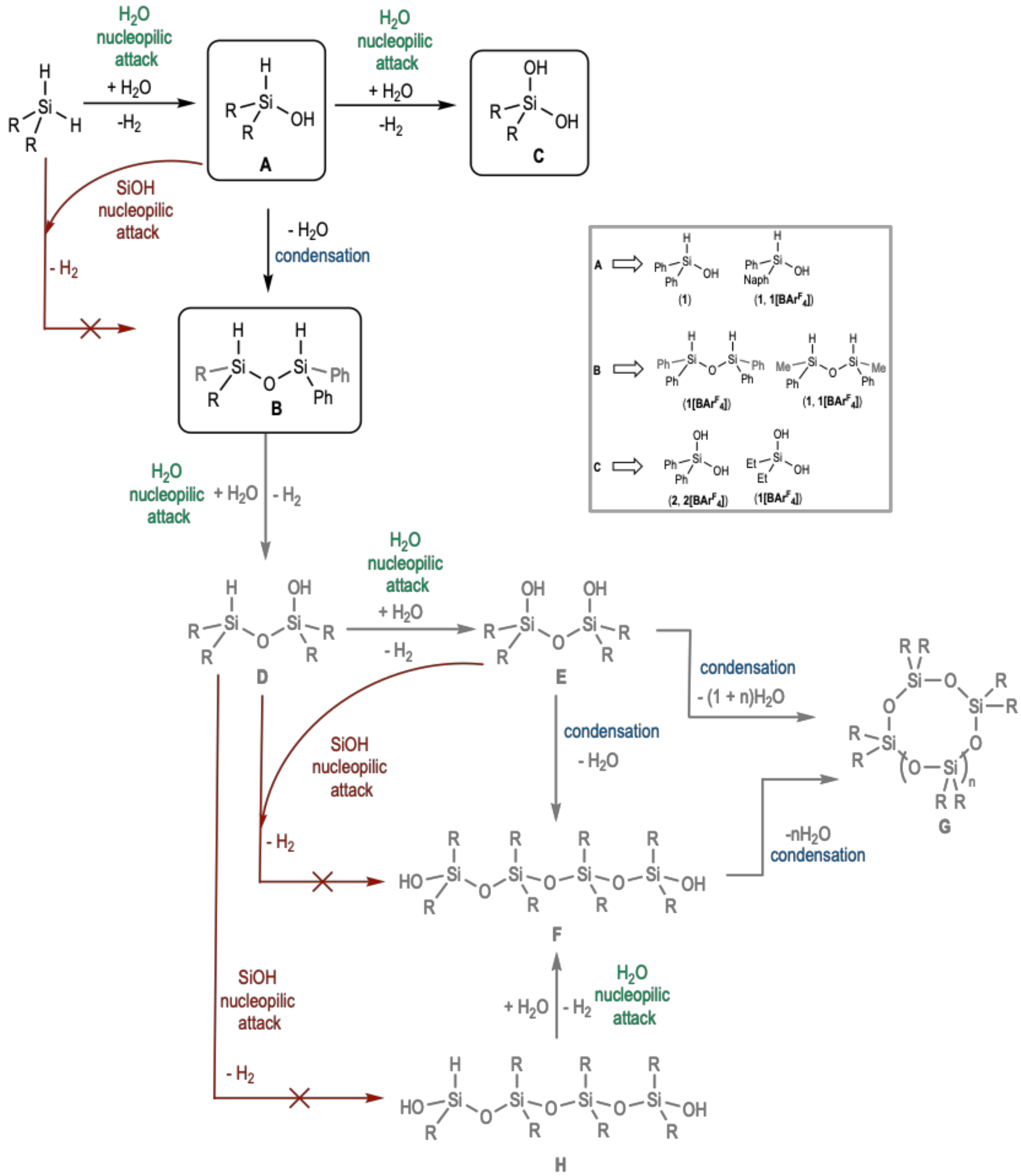

Figure S.25. Reactions involved in the formation of the different Si–O products found in this work. In black, identified and characterized products; in grey, potential polysiloxanes found in the reaction of dihydrosilanes with **1** and **1[BAr^F$_4$]** at long reaction times; in red, nucleophilic attack of silanol to hydrosilanes (not observed).

9. Hydrolysis and alcoholysis of other silanes

Alcoholysis of diphenylsilane: A closed reaction vessel equipped with a pressure transducer (Manonthemoon kinetic kit X102) was immersed in a thermostated ethylene glycol/water bath and charged with **1[BAr^F$_4$]** (0.00044 mmol) in 1 mL of distilled THF and R-OH (2.2 mmol). Once the pressure of the system was stabilized, the silane (0.22 mmol) was added, which was considered initial reaction time. The solution was left stirring until the pressure stabilized again, which was indicative that the reaction ended.

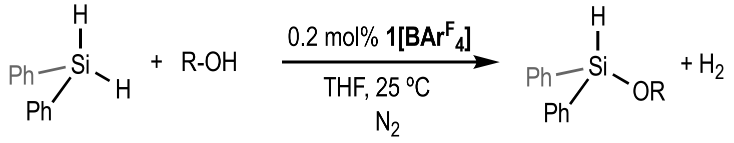

Table S.2. Alcoholysis of diphenylsilane

entry	Cat.	R-OH	H_2 equiv.	$TOF_{1/2}$ (h^{-1})	Product
1	**1[BAr^F$_4$]**	MeOH	0.90	64401	$Ph_2Si(H)OMe$
2	**1[BAr^F$_4$]**	EtOH	0.89	132591	$Ph_2Si(H)OEt$
3	**1[BAr^F$_4$]**	iPrOH	0.88	134570	$Ph_2Si(H)OiPr$

Hydrolysis of other silanes: A closed reaction vessel equipped with a pressure transducer (Manonthemoon kinetic kit X102) was immersed in a thermostated ethylene glycol/water bath and charged with **1[BAr^F$_4$]** (0.00044 mmol) in 1 mL of distilled THF and H_2O (2.2 mmol). Once the pressure of the system was stabilized, the silane (0.22 mmol) was added, which was considered initial reaction time. The solution was left stirring until the pressure stabilized again, which was indicative that the reaction ended.

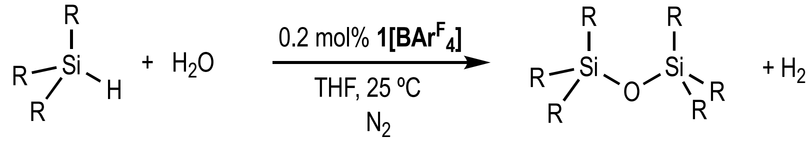

Table S.3. Hydrolysis of silanes

entry	Cat.	R_3Si-H	H_2 equiv.	$TOF_{1/2}$ (h^{-1})	Product
1	**1[BAr^F$_4$]**	Et_3Si-H	0.67	68	$Et_3SiOSiEt_3$
2	**1[BAr^F$_4$]**	Me_2PhSi-H	1	210	$Me_2PhSiOPhMe_2$
3	**1[BAr^F$_4$]**	$MePh_2Si$-H	0.91	41	$MePh_2SiOPh_2Me$
5	**1[BAr^F$_4$]**	$PhSiH_3$	2	589294	unidentified

10. X-ray crystallography tables

Table S.4. Crystallographic data and structure refinement details of all compounds.

	**1**	**2**	**1[BAr$^F_4$]**	**2[BAr$^F_4$]**
CCDC number	2218419	2218421	2218420	2218418
Formula	$C_{21}H_{22}Cl_{2.5}P_1Rh_{0.5}Si_1$	$C_{40}H_{40}ClIrP_2Si_2$	$C_{74}H_{55}BF_{24}NP_2RhSi_2$	$C_{76}H_{58}BF_{24}N_2P_2IrSi_2$
M	473.52	1827.60	1643.06	1776.37
Crystal System	monoclinic	triclinic	monoclinic	monoclinic
Space group	*C 2/c*	*P -1*	*C 2/c*	*C1 2/c 1*
T [K]	293	293	100	100
a [Å]	16.9660(8)	11.141(5)	35.789(3)	34.724(5)
b [Å]	12.4430(8)	12.533(5)	21.685(2)	22.681(5)
c [Å]	20.3710(11)	13.449(5)	23.980(2)	24.048(5)
α [deg]	90	93.560(5)	90	90
β [deg]	97.506(2)	92.528(5)	128.886(3)	128.588(5)
γ [deg]	90	102.365(5)	90	90
V [Å^3]	4263.6(4)	1827.6(13)	14486(5)	2497.28(15)
Z	8	2	8	8
Density [gcm^{-3}]	1.475	1.575	1.509	1.594
μ [mm^{-1}]	0.875	3.907	0.418	1.985
Observed reflections	5530	5160	18293	19209
R_1 [I > 2σ(I)]	0.037	0.085	0.062	0.043
wR_2 [all data]	0.092	0.212	0.159	0.116
GoF	1.135	1.186	1.148	1.102

[a] $\sum||F_o|-|F_c||/\sum|F_o|$ [b] $\{\sum [w(F_o^2-F_c^2)^2]/ \sum [w(F_o^2)^2]\}^{1/2}$

11. NMR and FTIR spectra of compounds

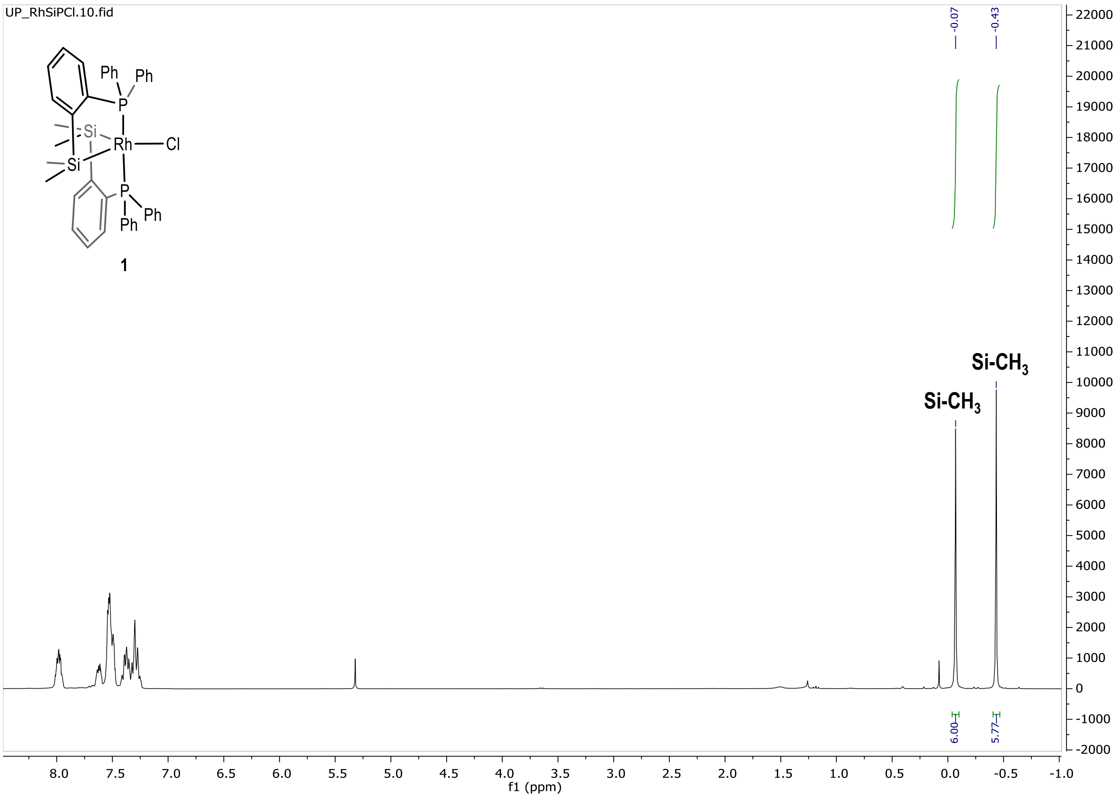

Figure S. 26. ^{1}H NMR spectrum in CD_2Cl_2 of **1**.

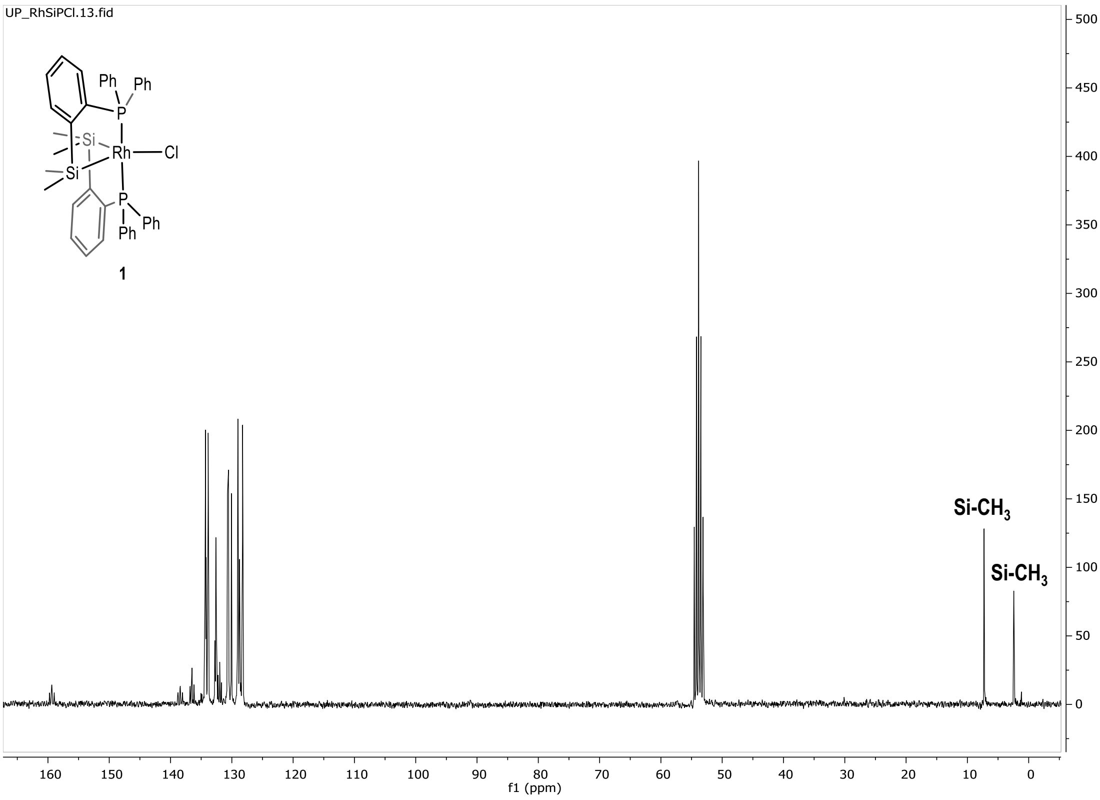

Figure S.27. $^{13}C\{^1H\}$ NMR spectrum in CD_2Cl_2 of **1**.

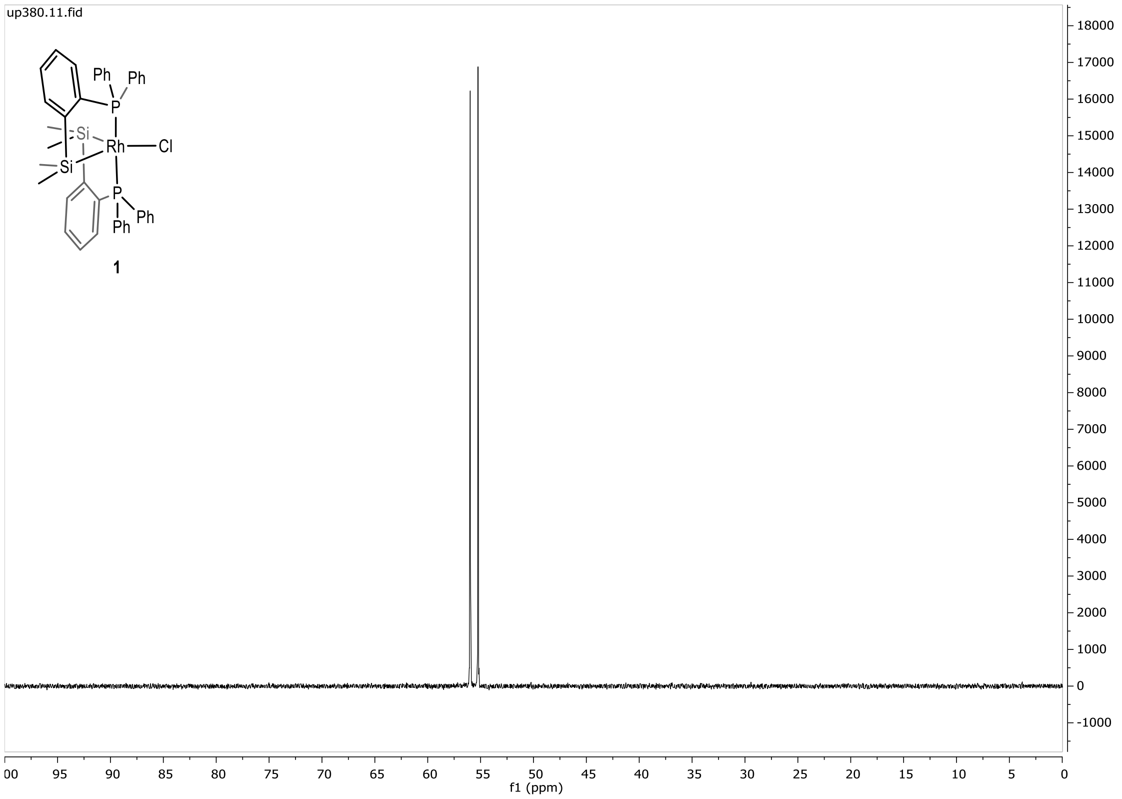

Figure S.28. $^{31}P\{^{1}H\}$ NMR spectrum in CD_2Cl_2 of **1**.

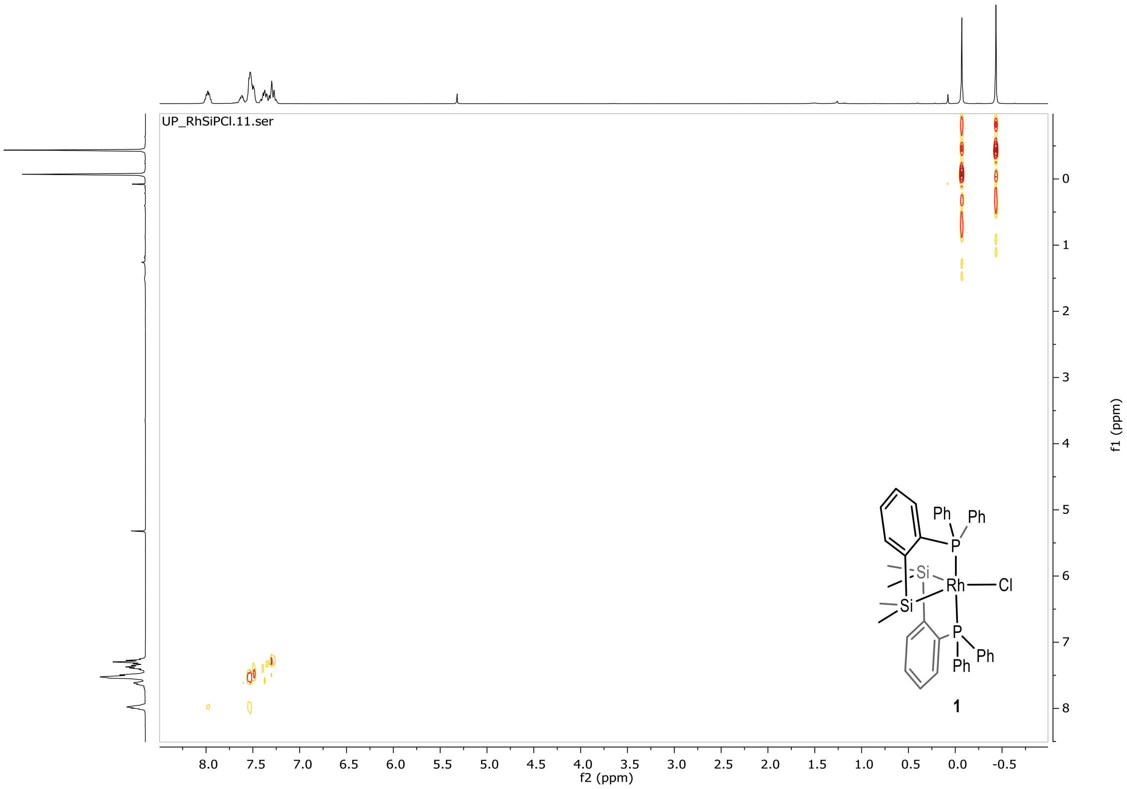

Figure S.29. COSY spectrum in CD_2Cl_2 of **1**.

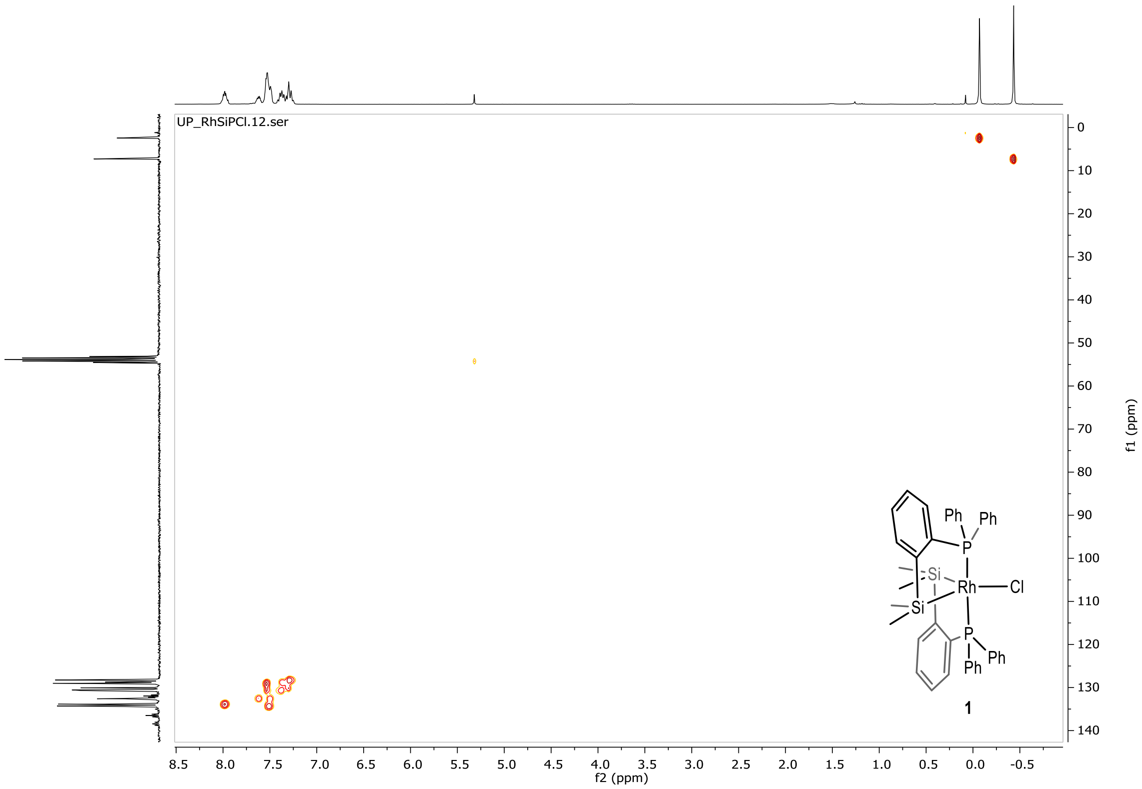

Figure S.30. HSQC spectrum in CD_2Cl_2 of **1**.

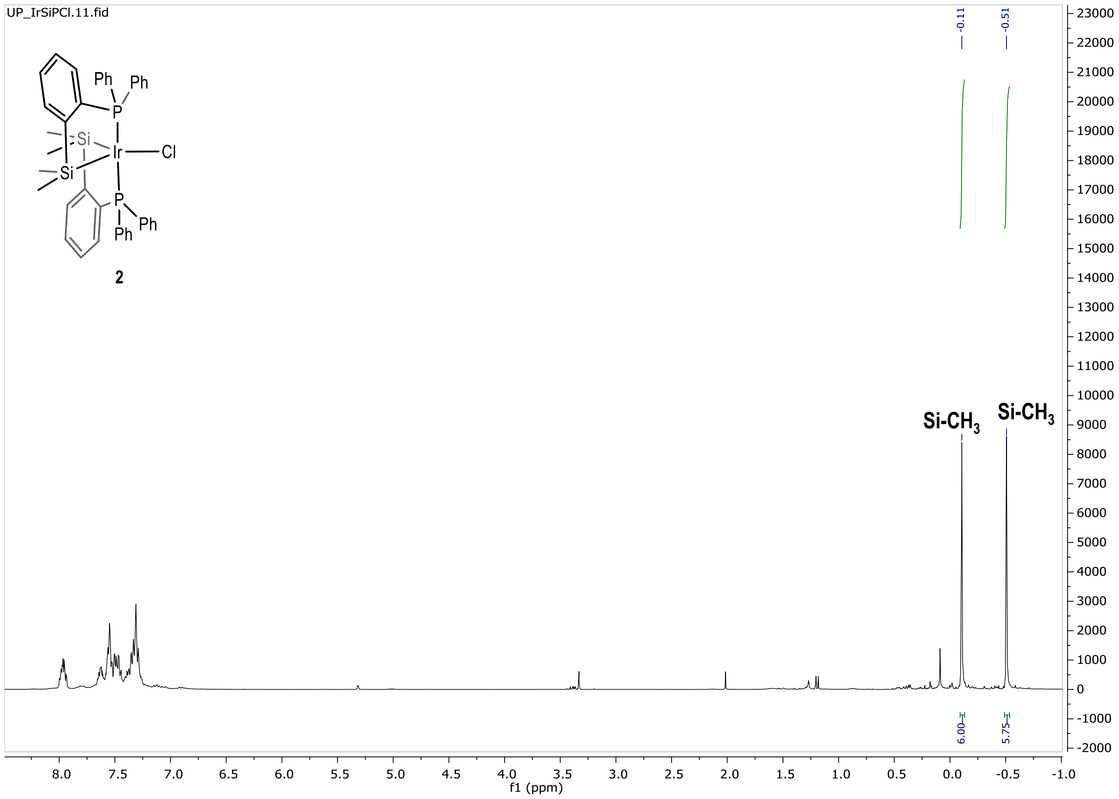

Figure S.31. ^{1}H NMR spectrum in CD_2Cl_2 of **2**.

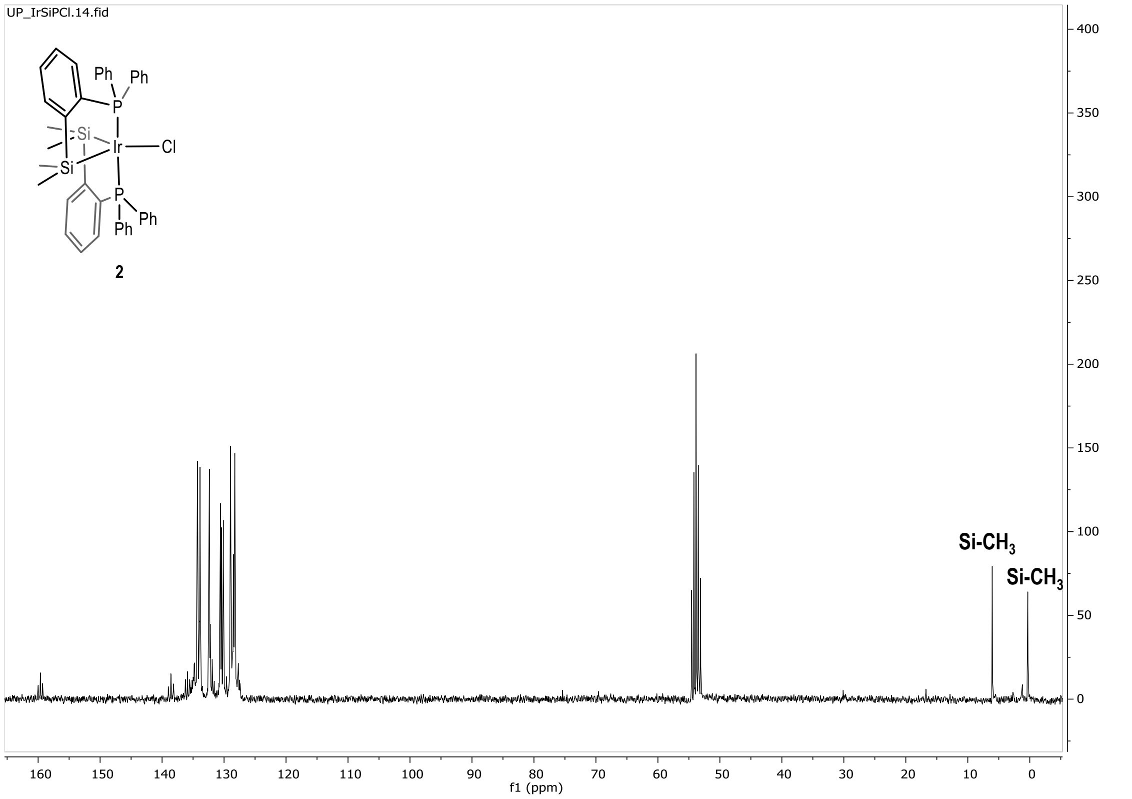

Figure S.32. $^{13}C\{^{1}H\}$ NMR spectrum in CD_2Cl_2 of **2**.

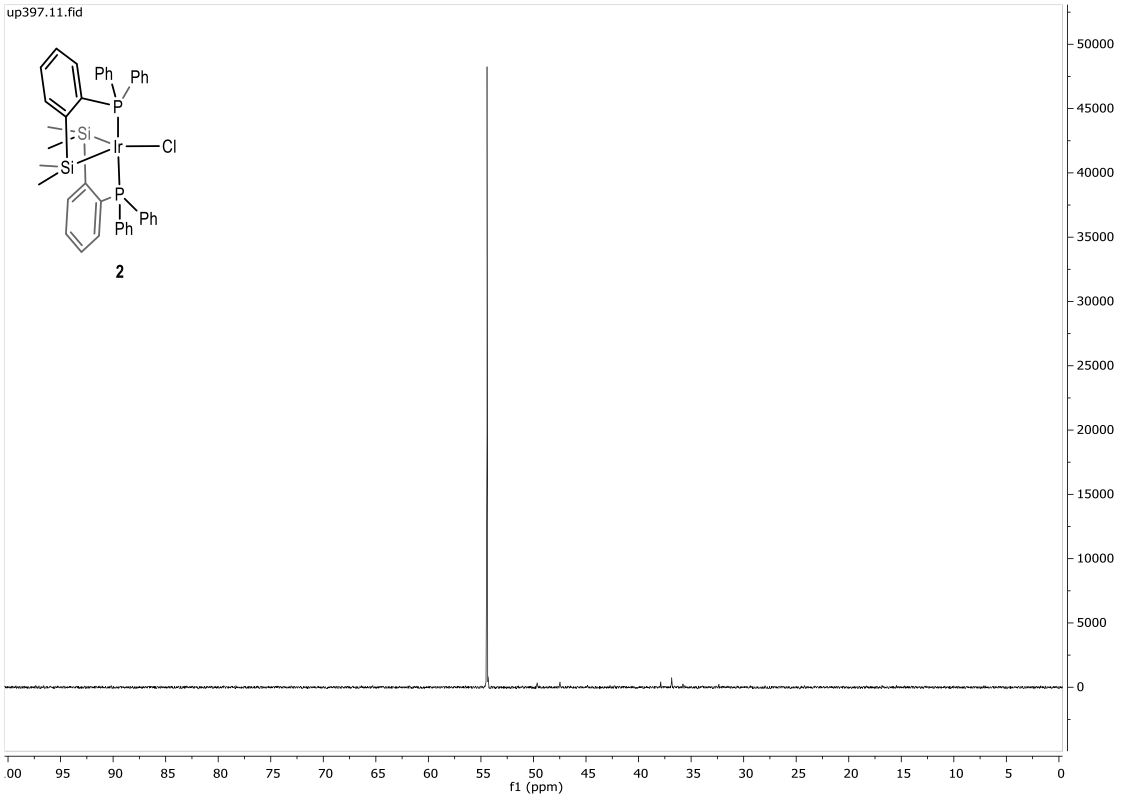

Figure S.33. $^{31}P\{^{1}H\}$ NMR spectrum in CD_2Cl_2 of **2**.

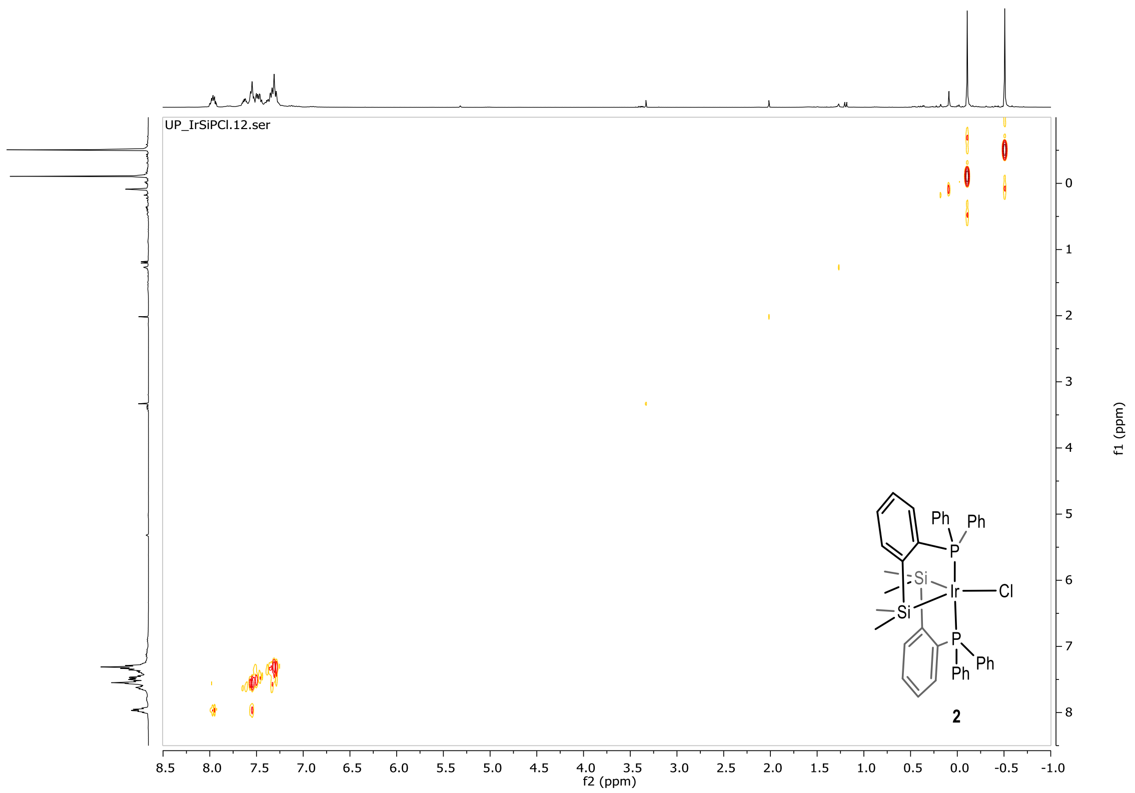

Figure S.34. COSY spectrum in CD_2Cl_2 of **2**.

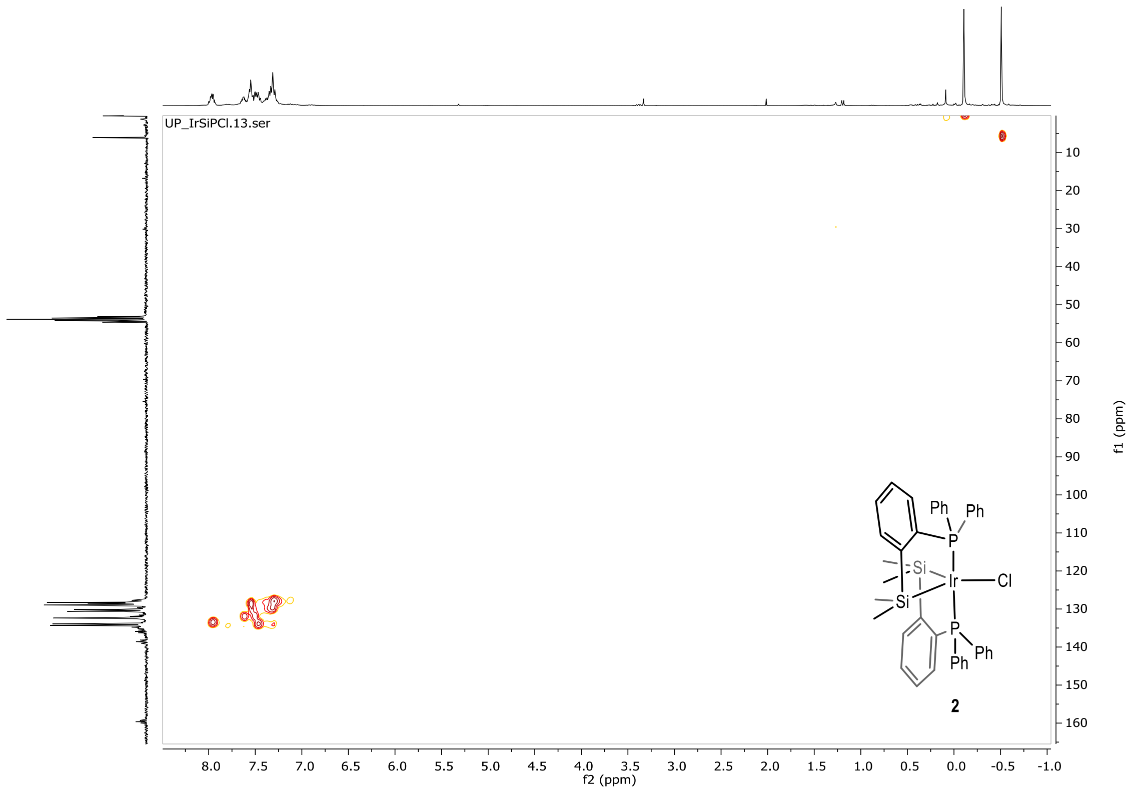

Figure S.35. HSQC spectrum in CD_2Cl_2 of **2**.

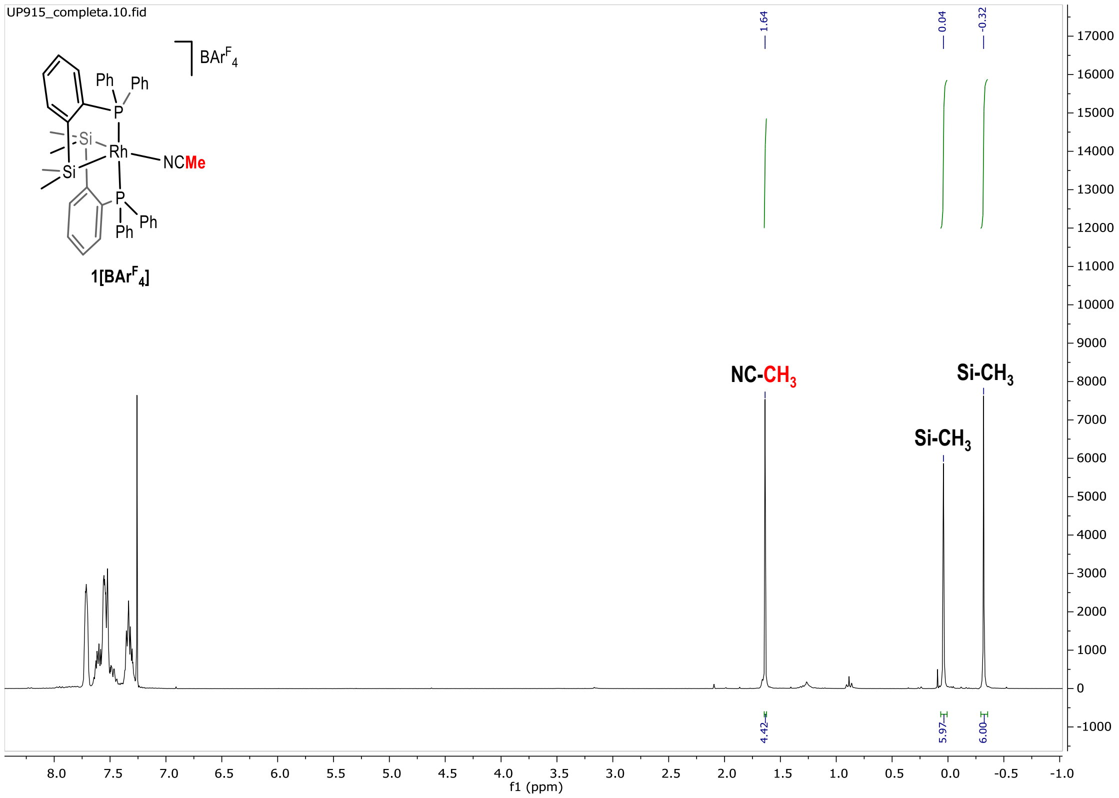

Figure S.36. ^{1}H NMR spectrum in $CDCl_3$ of **1[BAr$^F_4$]**.

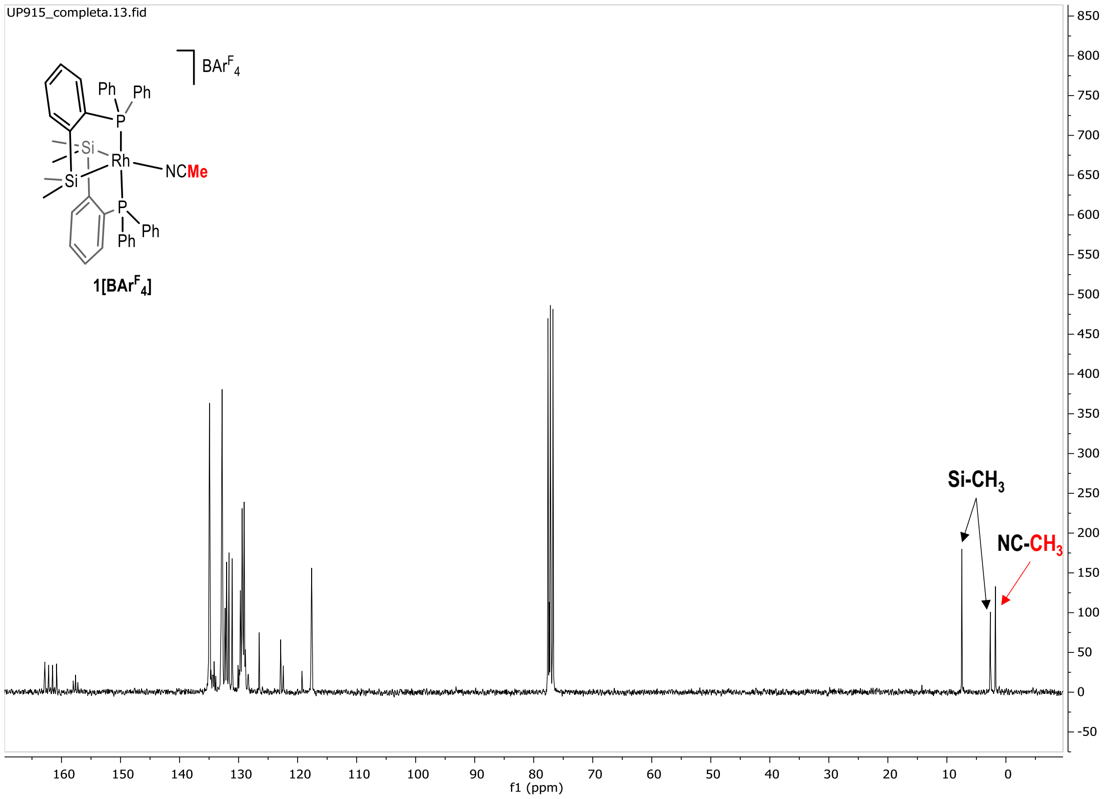

Figure S.37. $^{13}C\{^1H\}$ NMR spectrum in $CDCl_3$ of **1[BAr$^F_4$]**.

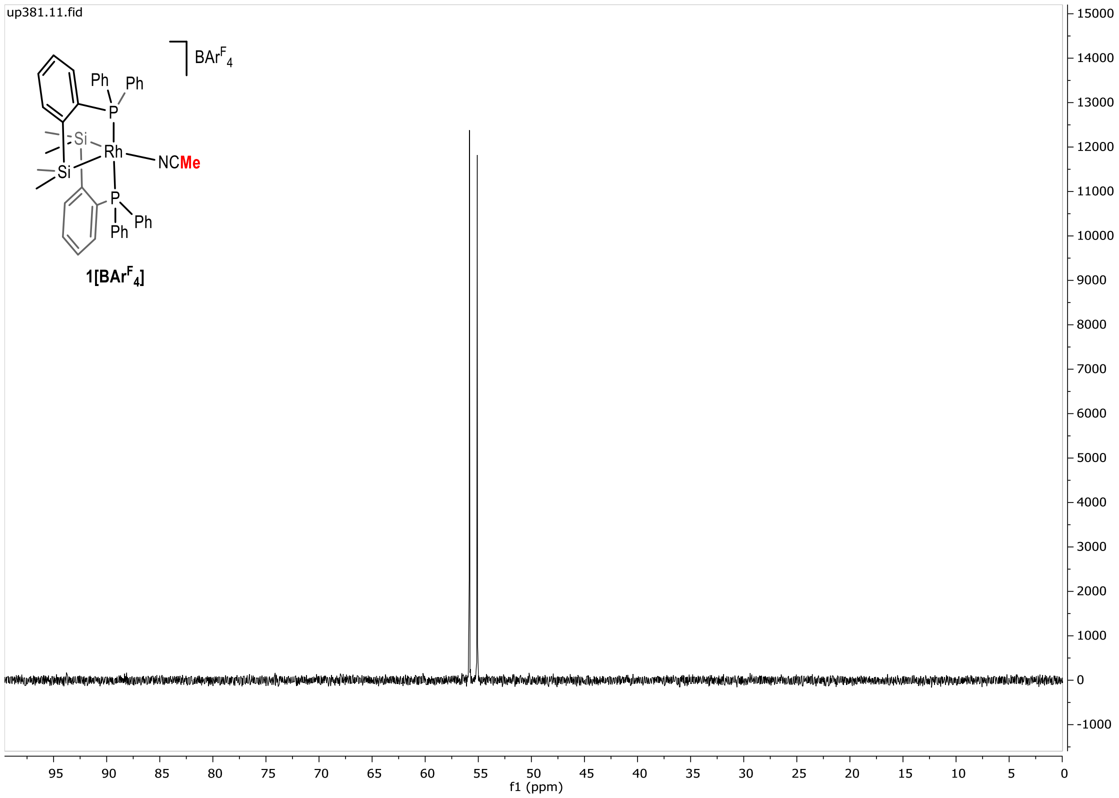

Figure S.38. $^{31}P\{^1H\}$ NMR spectrum in $CDCl_3$ of **1[BAr$^F_4$]**.

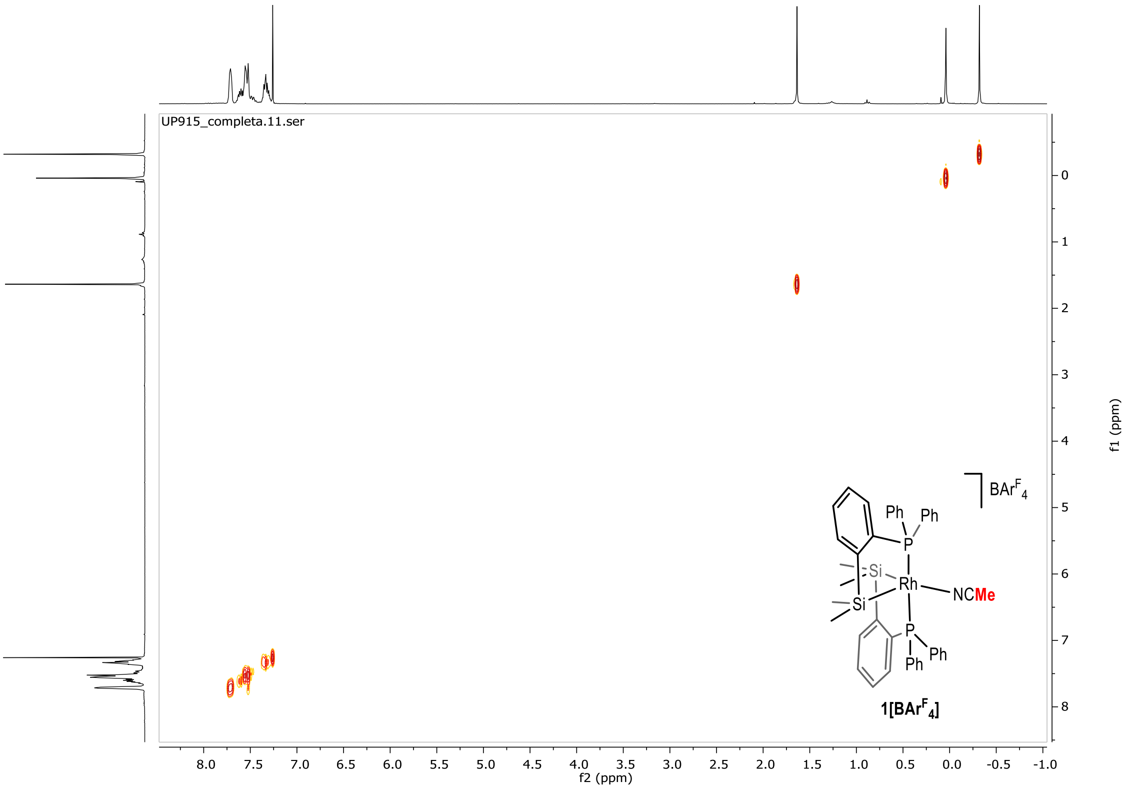

Figure S.39. COSY spectrum in $CDCl_3$ of **1[BAr$^F_4$]**.

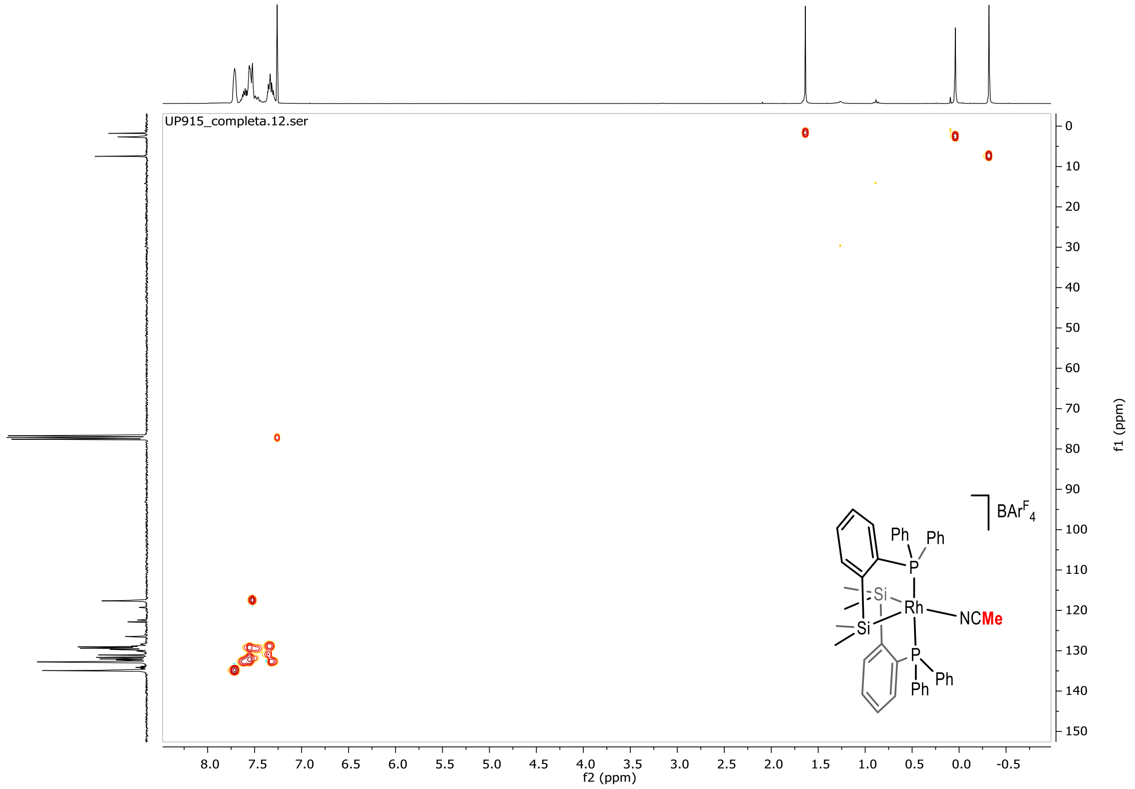

Figure S.40. HSQC spectrum in $CDCl_3$ of **1[BAr$^F_4$]**.

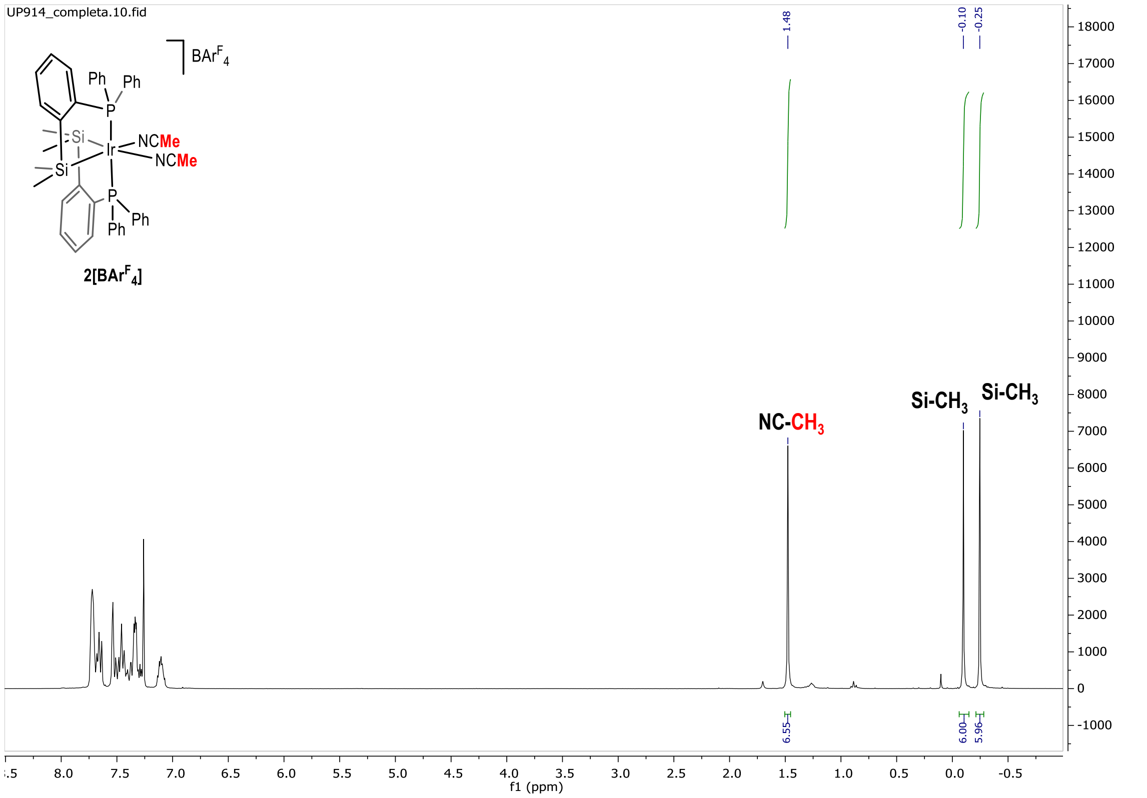

Figure S.41. ^1H NMR spectrum in $CDCl_3$ of **2[BAr$^F_4$]**.

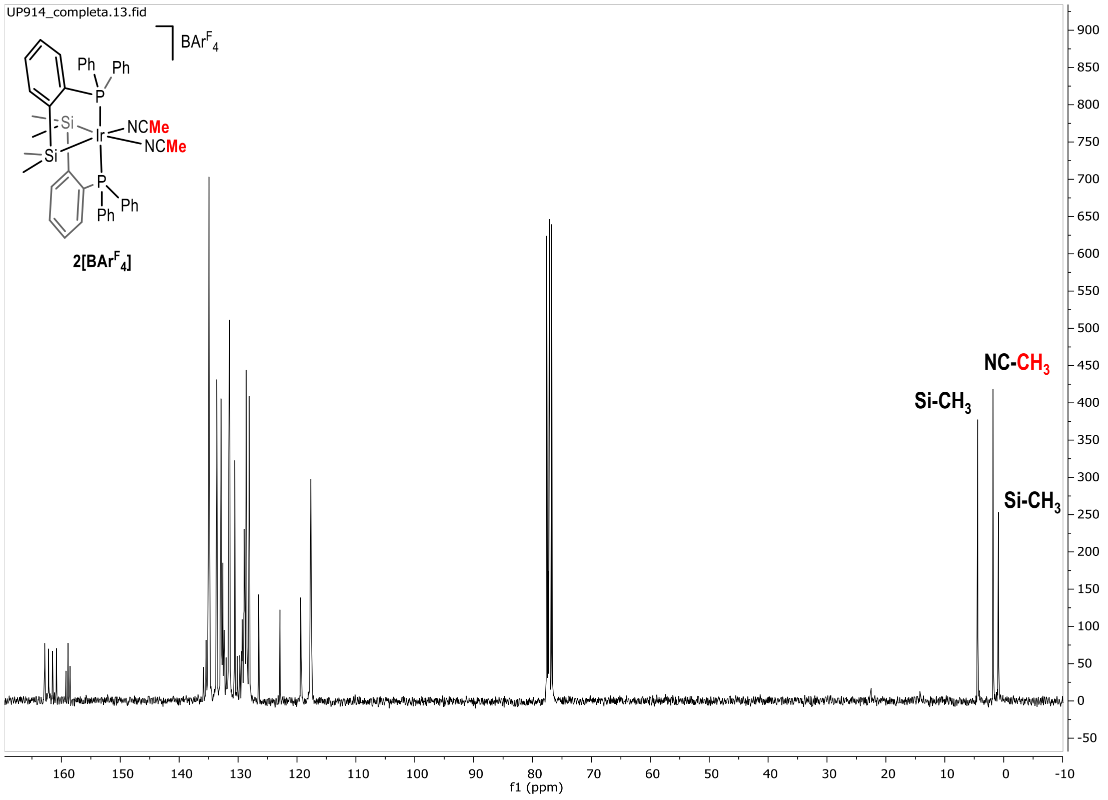

Figure S.42. $^{13}C\{^{1}H\}$ NMR spectrum in $CDCl_3$ of **2[BAr$^F_4$]**.

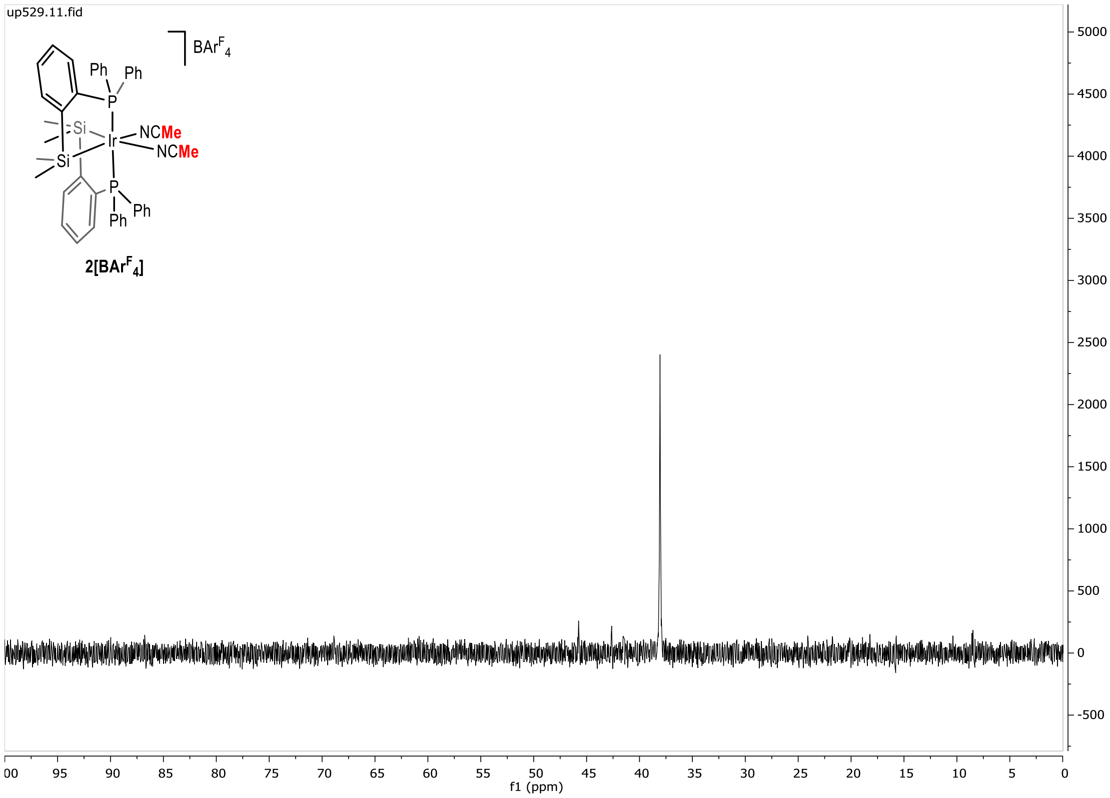

Figure S.43. $^{31}P\{^{1}H\}$ NMR spectrum in $CDCl_3$ of **2[BAr$^F_4$]**

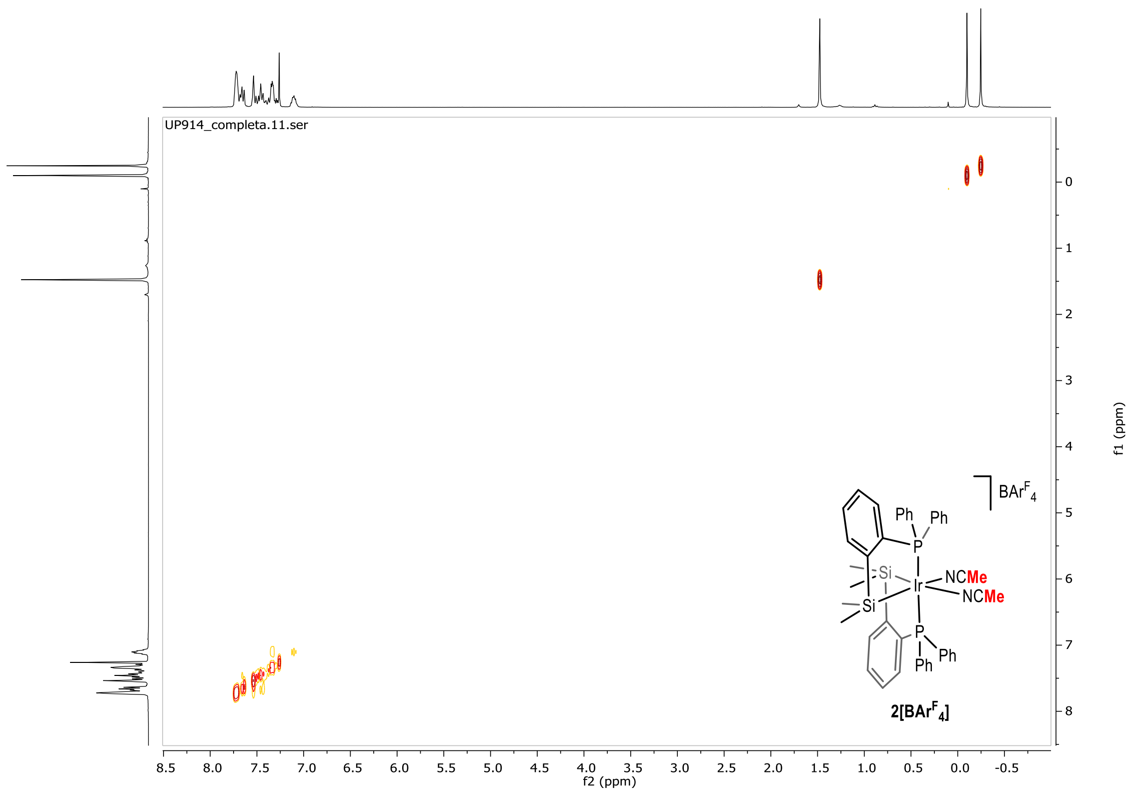

Figure S.44. COSY spectrum in $CDCl_3$ of **2[BArF4]**.

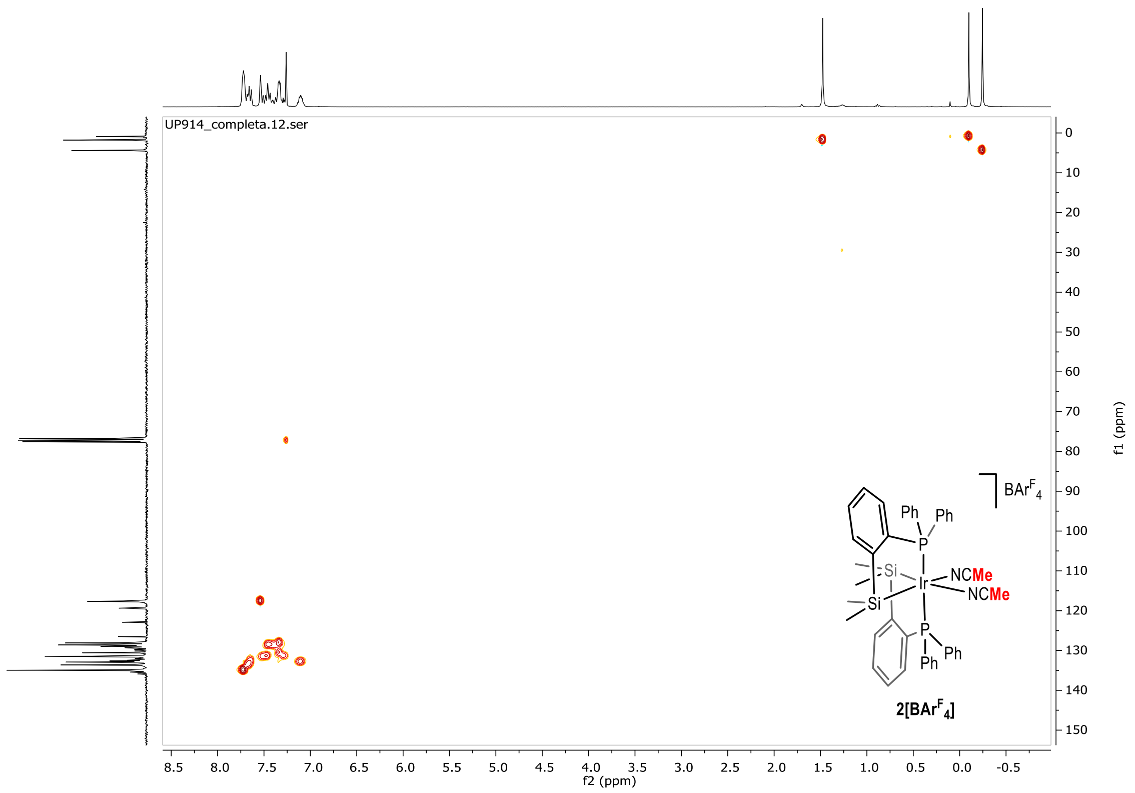

Figure S.45. HSQC spectrum in $CDCl_3$ of **2[BArF4]**.

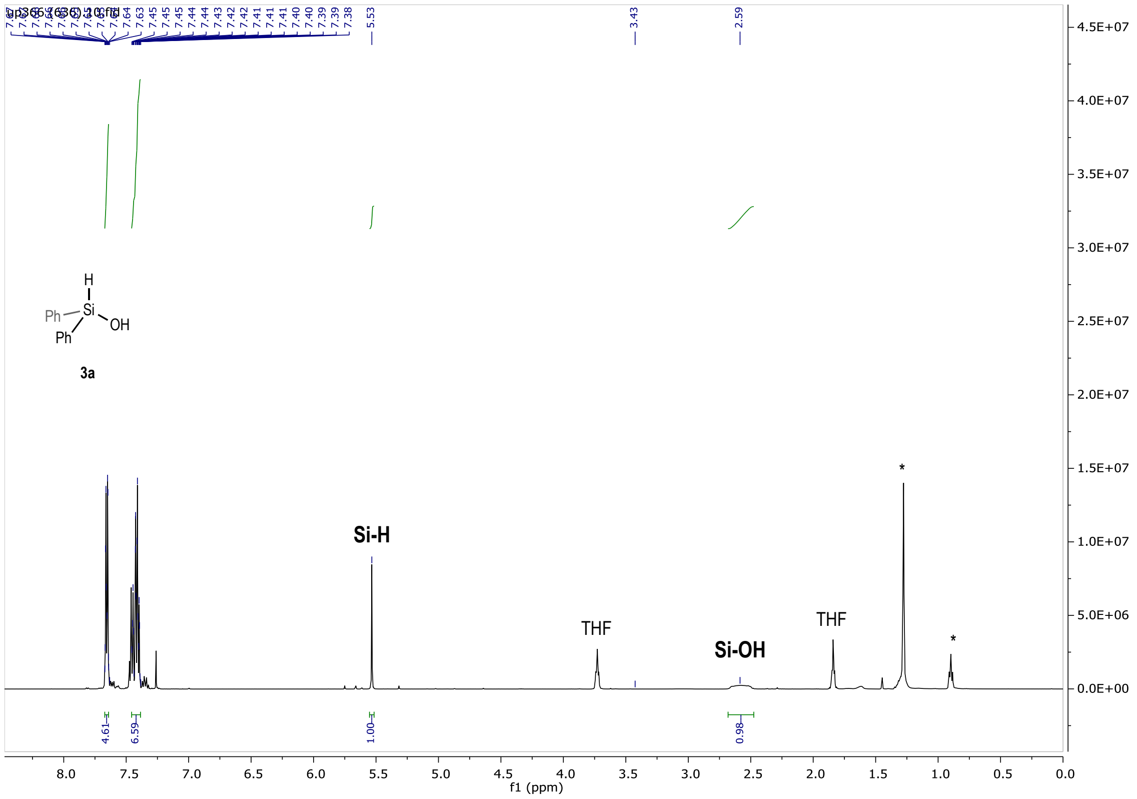

Figure S.46. ^{1}H NMR spectrum in $CDCl_3$ of **3a**. (*) 10/11 carbon-chain alkane, hexane impurities.

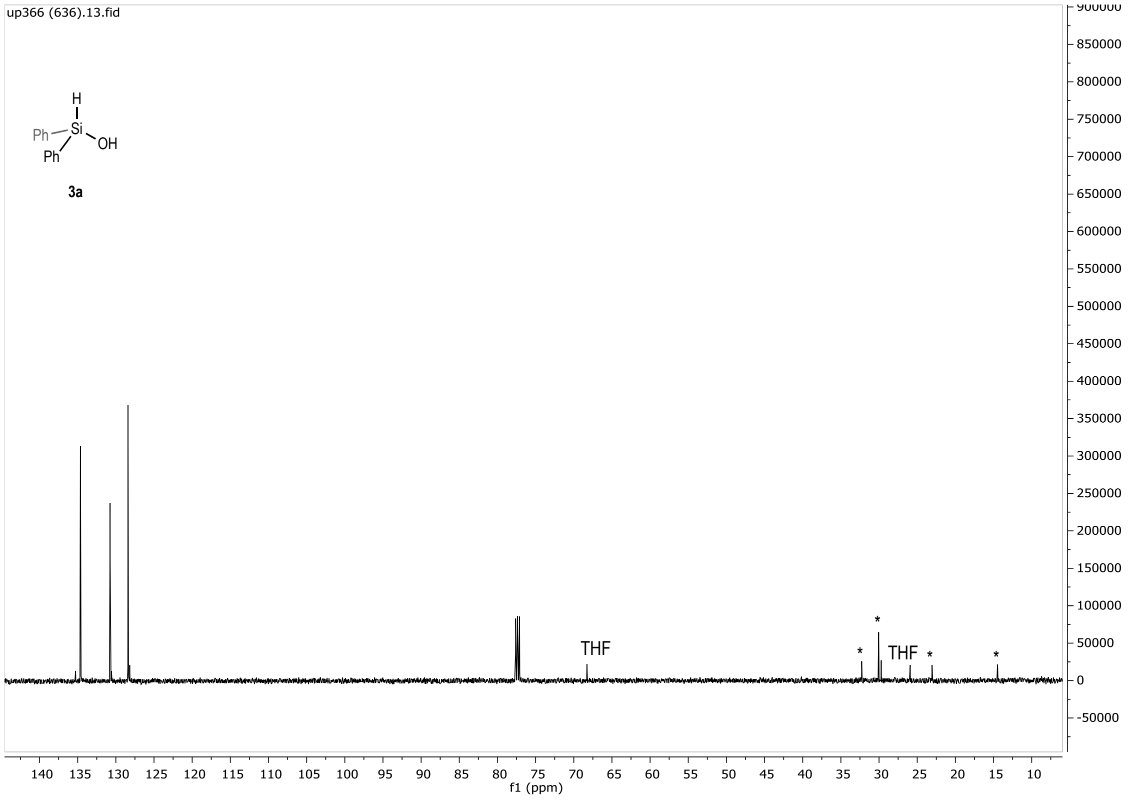

Figure S.47. ^{13}C{^{1}H} NMR spectrum in $CDCl_3$ of **3a**. (*) 10/11 carbon-chain alkane, hexane impurities.

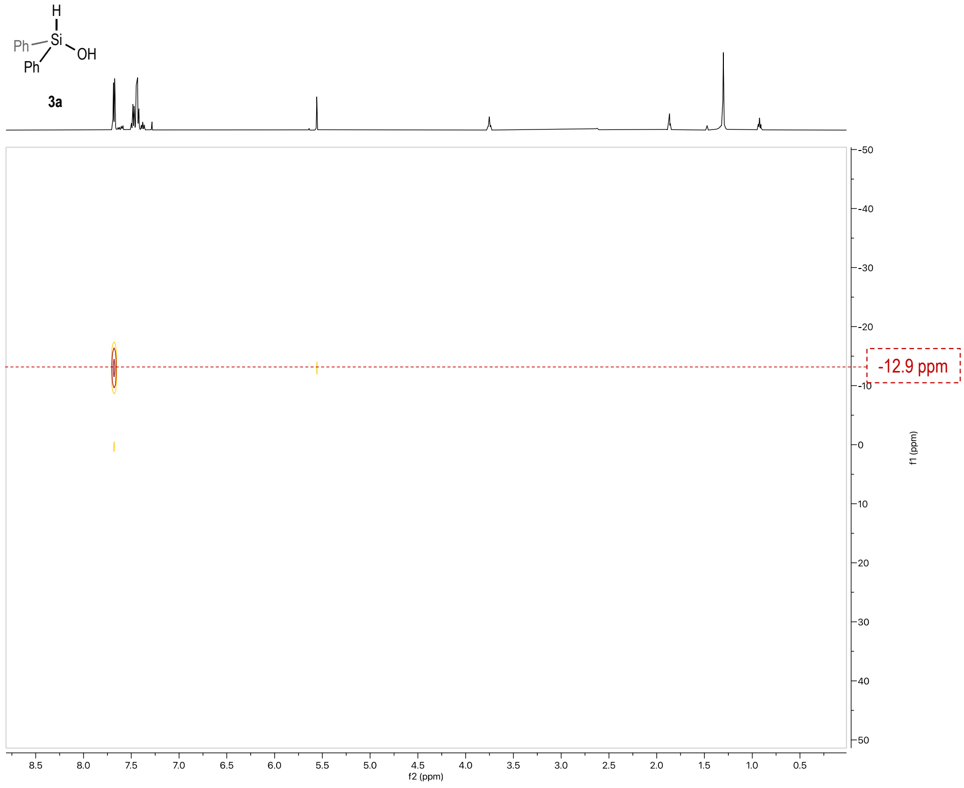

Figure S.48. HMQC (^1H - ^{29}Si) NMR spectrum in $CDCl_3$ of **3a**.

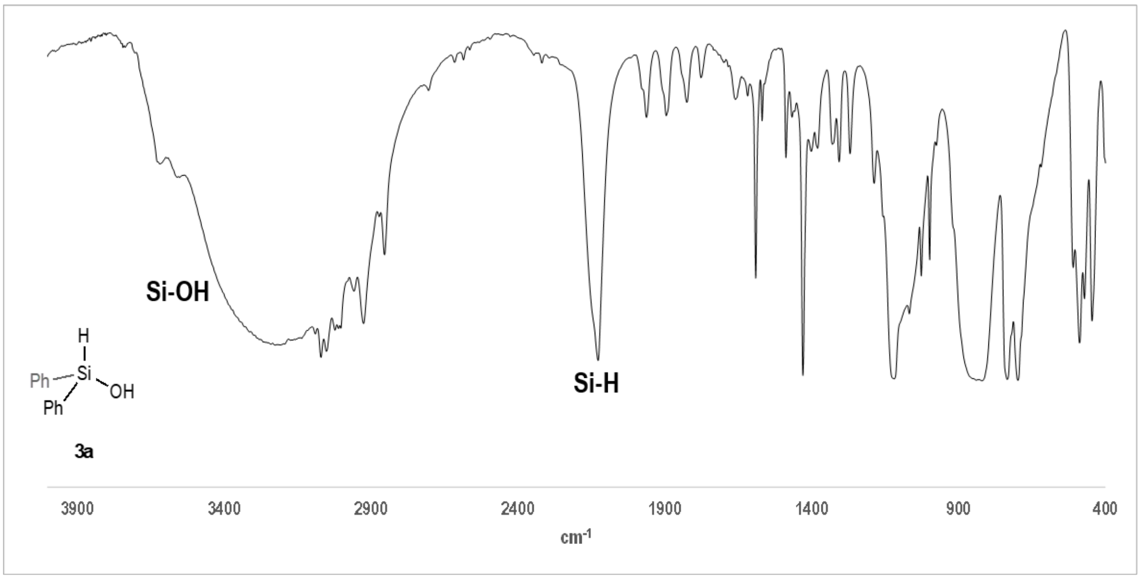

Figure S.49. FTIR spectrum of **3a.**

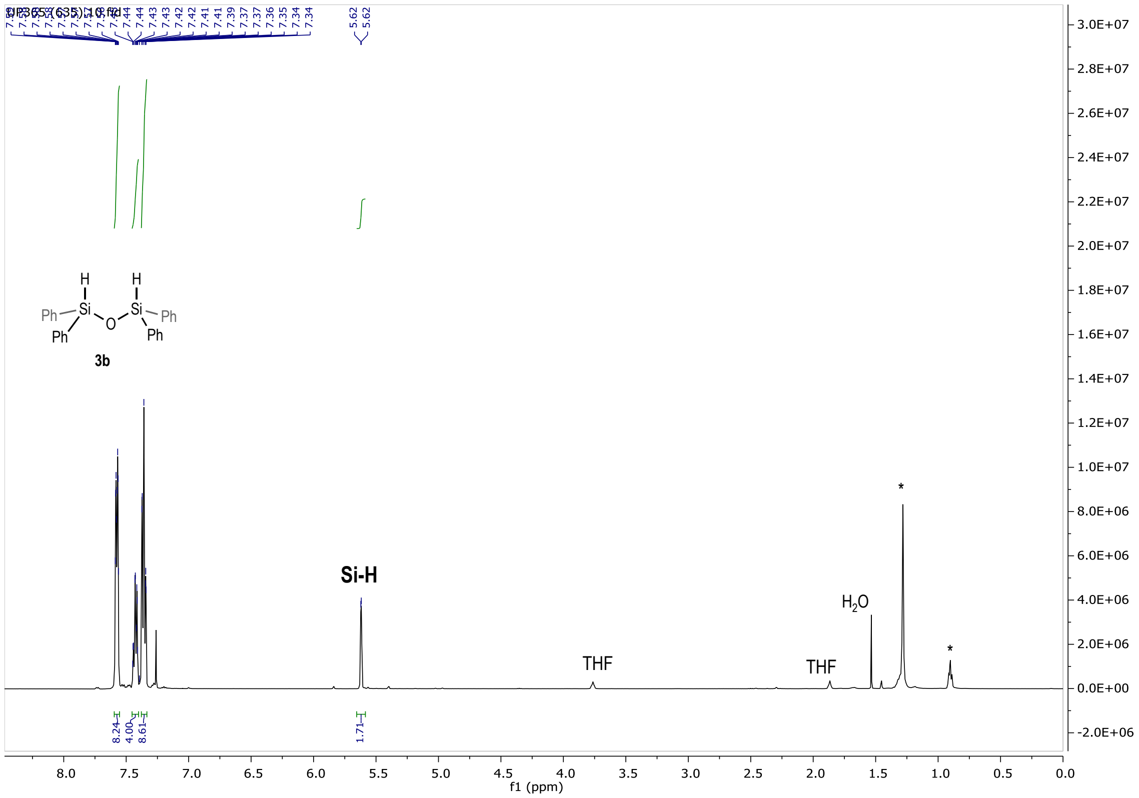

Figure S.50. ^{1}H NMR spectrum in $CDCl_3$ of **3b**. (*) 10/11 carbon-chain alkane, hexane impurities.

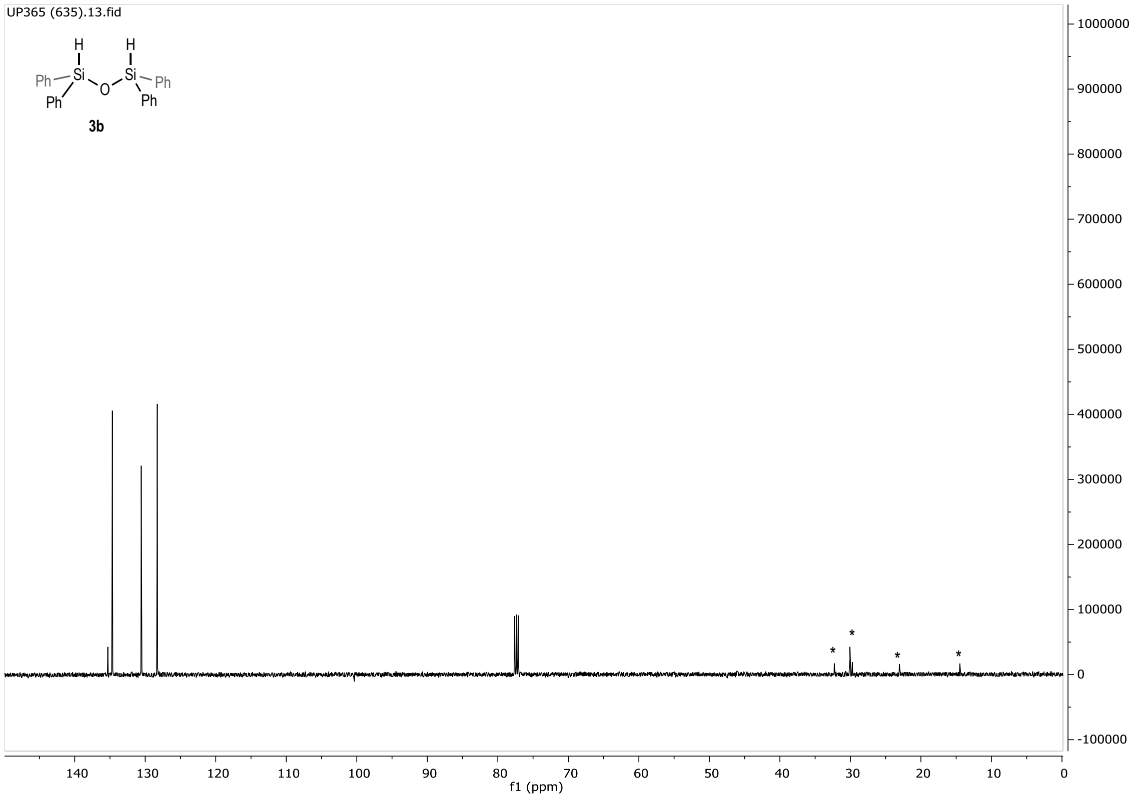

Figure S.51. $^{13}C\{^{1}H\}$ NMR spectrum in $CDCl_3$ of **3b**. (*) 10/11 carbon-chain alkane, hexane impurities.

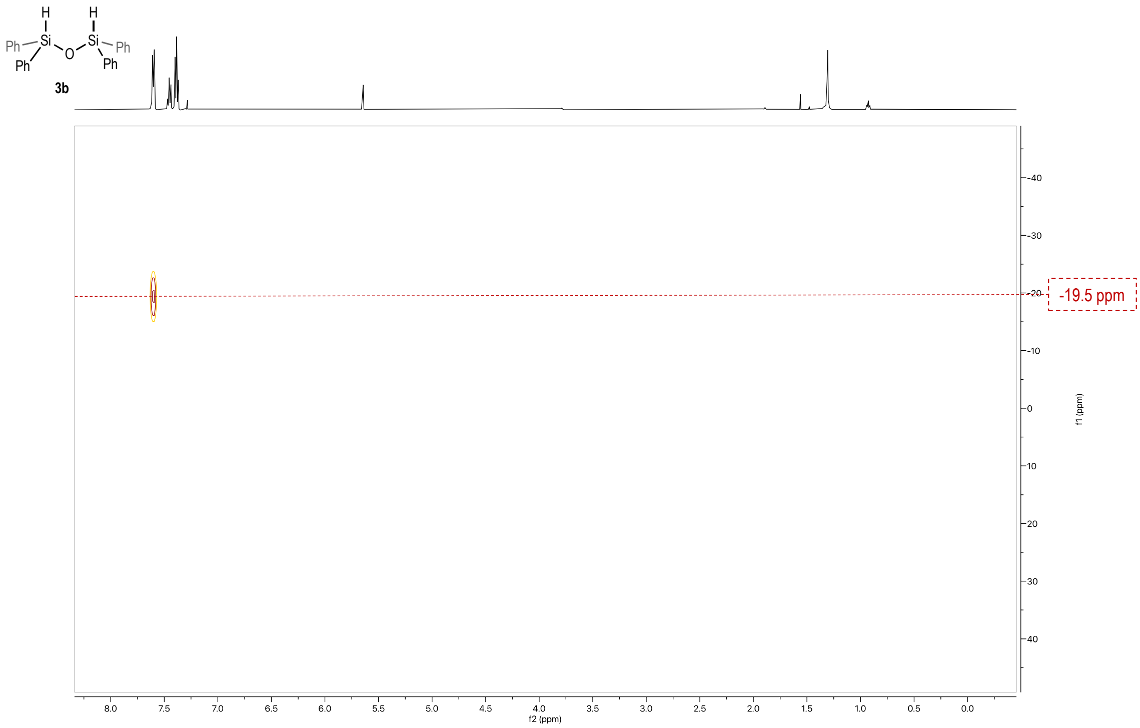

Figure S.52. HMQC (^1H - ^{29}Si) NMR spectrum in $CDCl_3$ of **3b**.

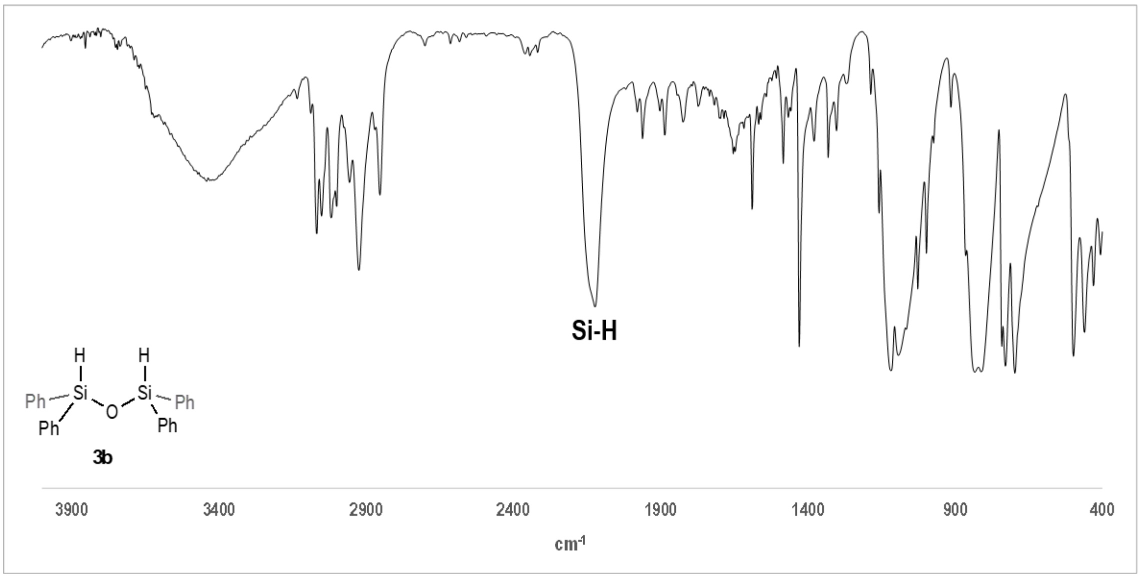

Figure S.53. FTIR spectrum of **3b.**

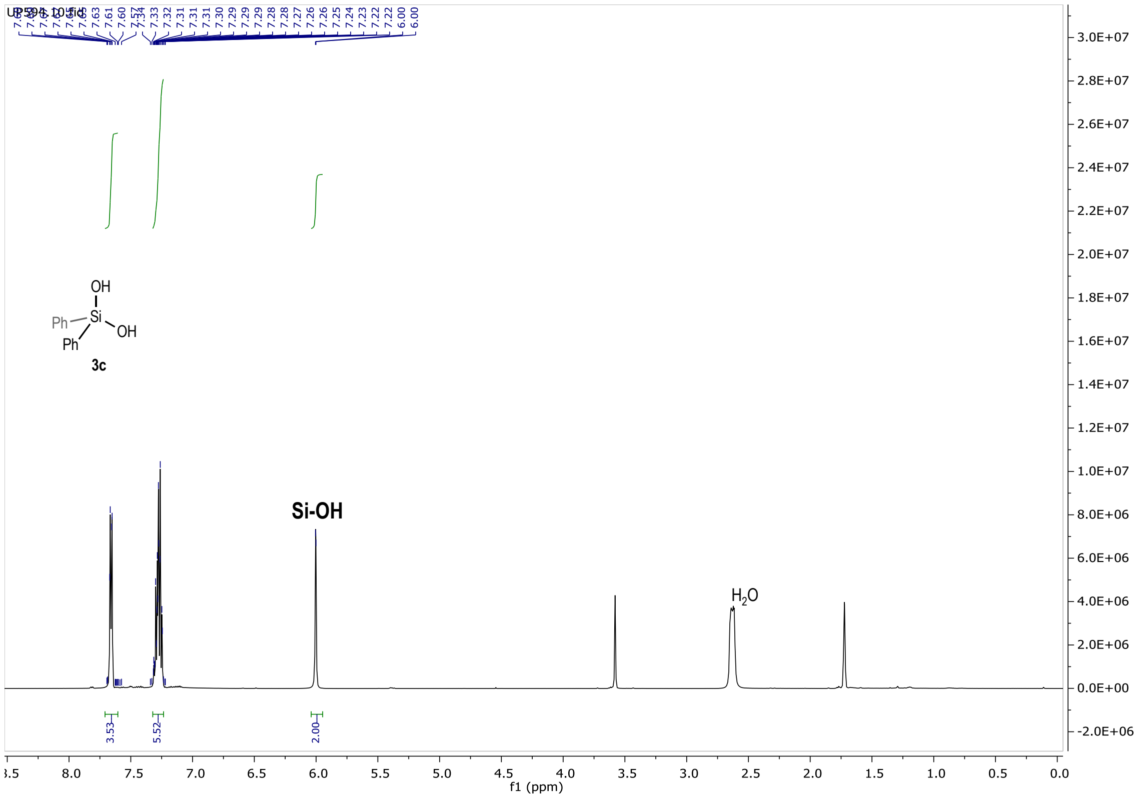

Figure S.54 ^{1}H NMR spectrum in TFH-d_8 of **3c**.

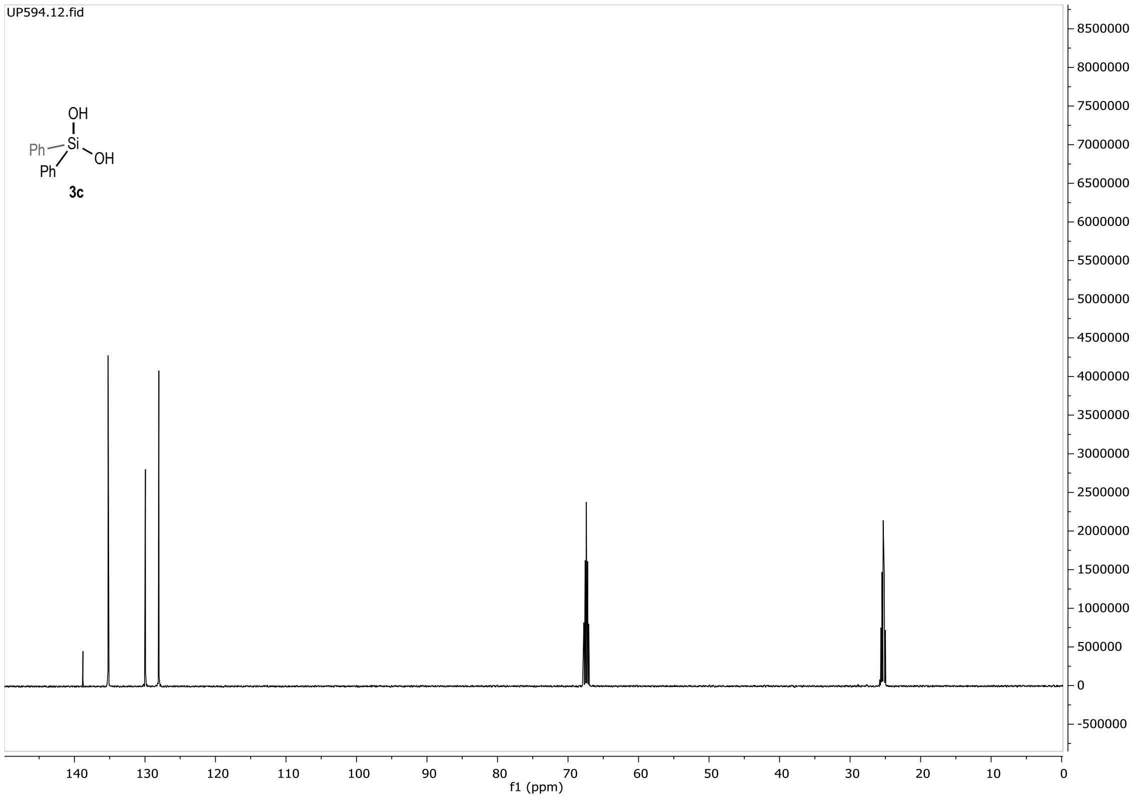

Figure S.55. $^{13}C\{^{1}H\}$ NMR spectrum in TFH-d_8 of **3c**.

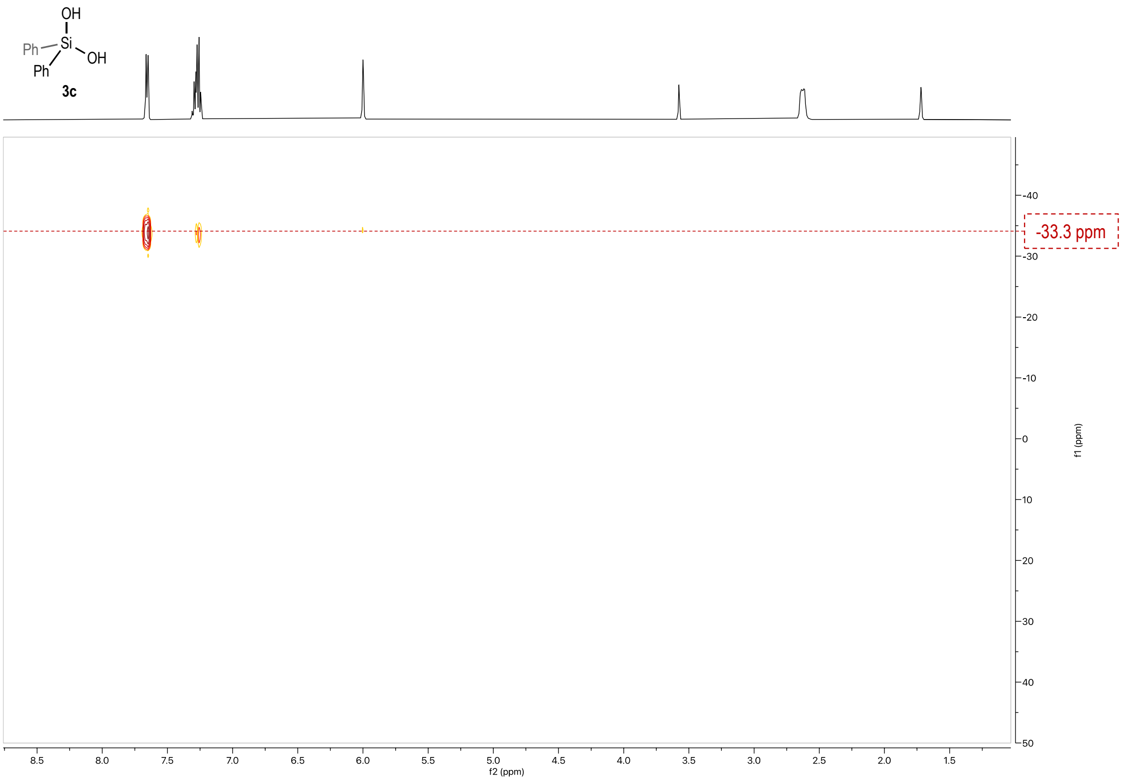

Figure S.56. HMQC (^1H - ^{29}Si) NMR spectrum in TFH-d_8 of **3c**.

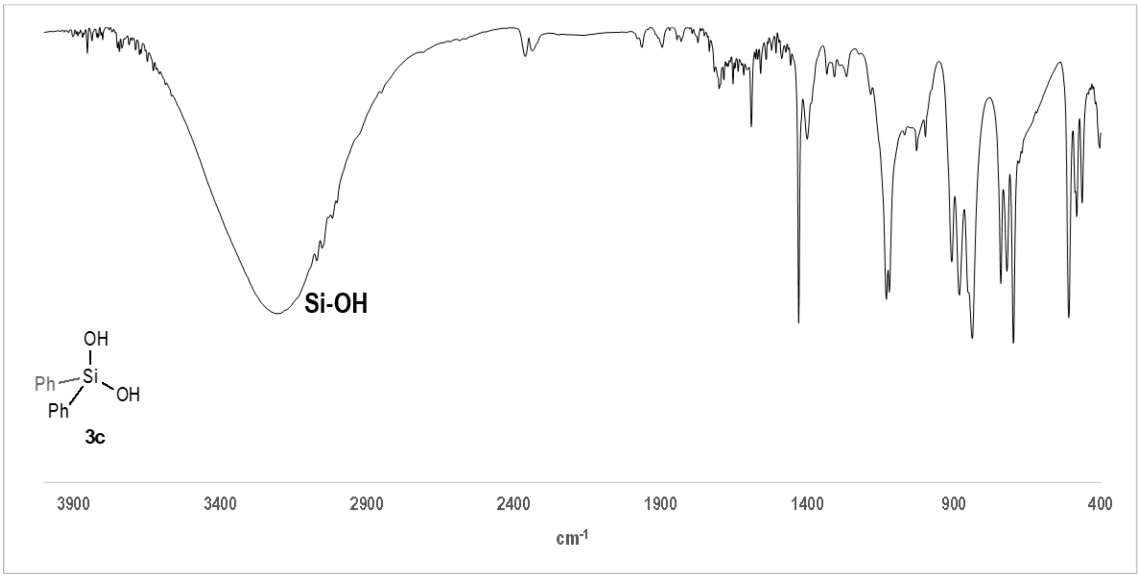

Figure S.57. FTIR spectrum of **3c.**

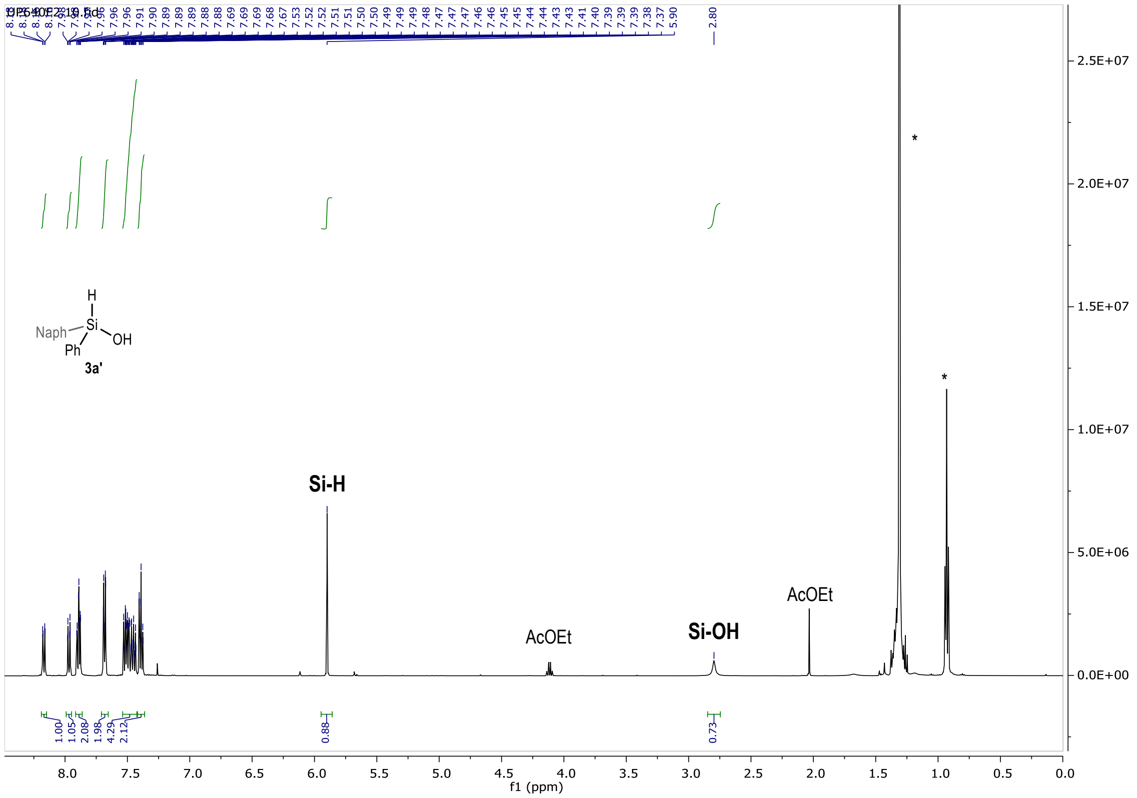

Figure S.58. ^{1}H NMR spectrum in $CDCl_3$ of **3a'**. (*) 10/11 carbon-chain alkane, hexane impurities.

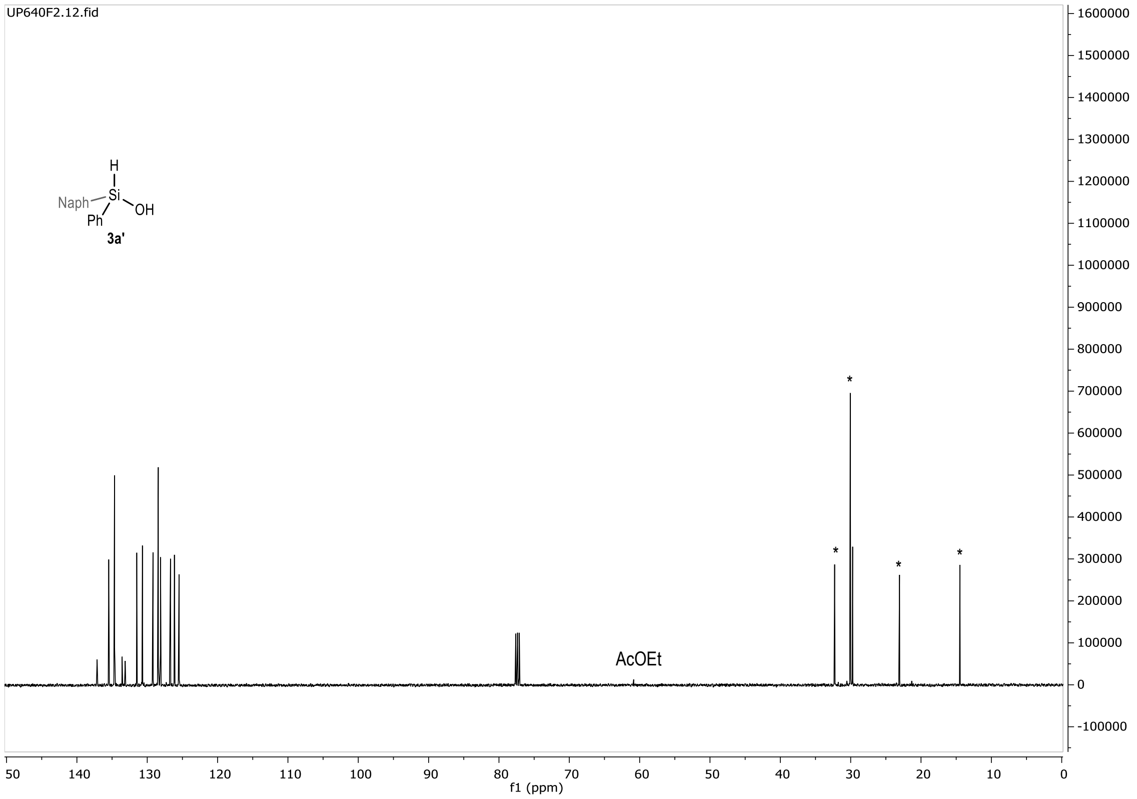

Figure S.59. $^{13}C\{^{1}H\}$ NMR spectrum in $CDCl_3$ of **3a'**. (*) 10/11 carbon-chain alkane, hexane impurities.

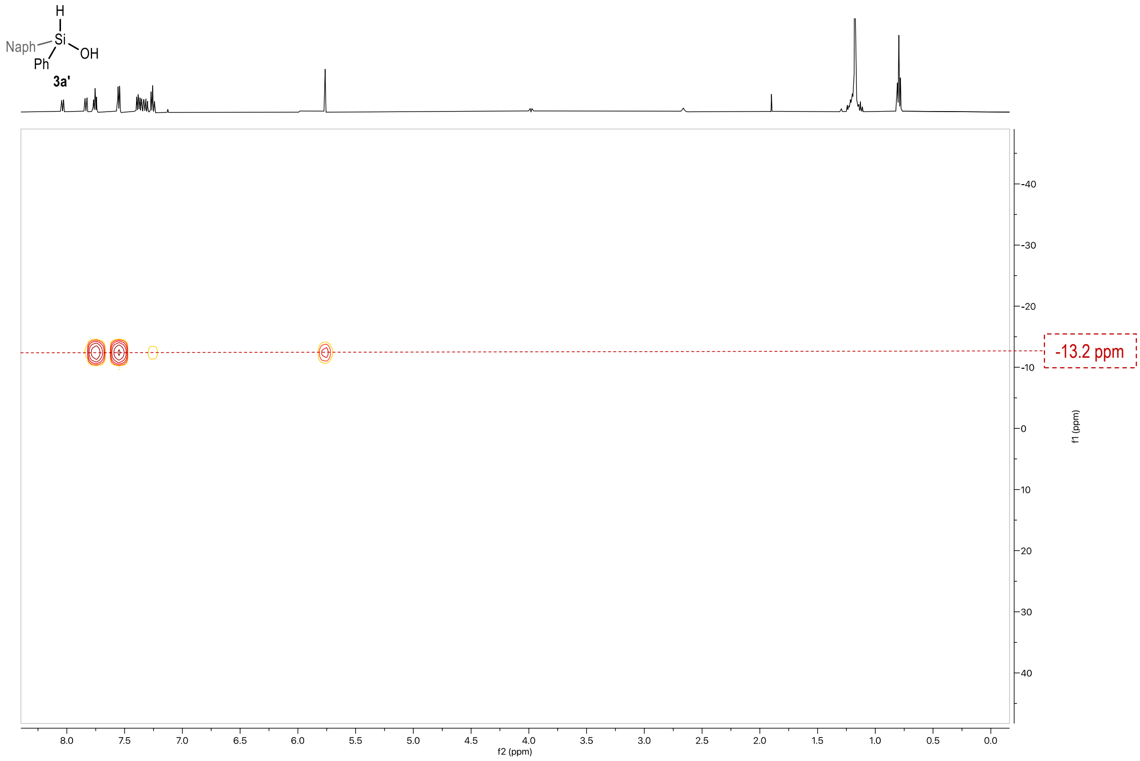

Figure S.60. HMQC (^1H - ^{29}Si) NMR spectrum in $CDCl_3$ of **3a'**.

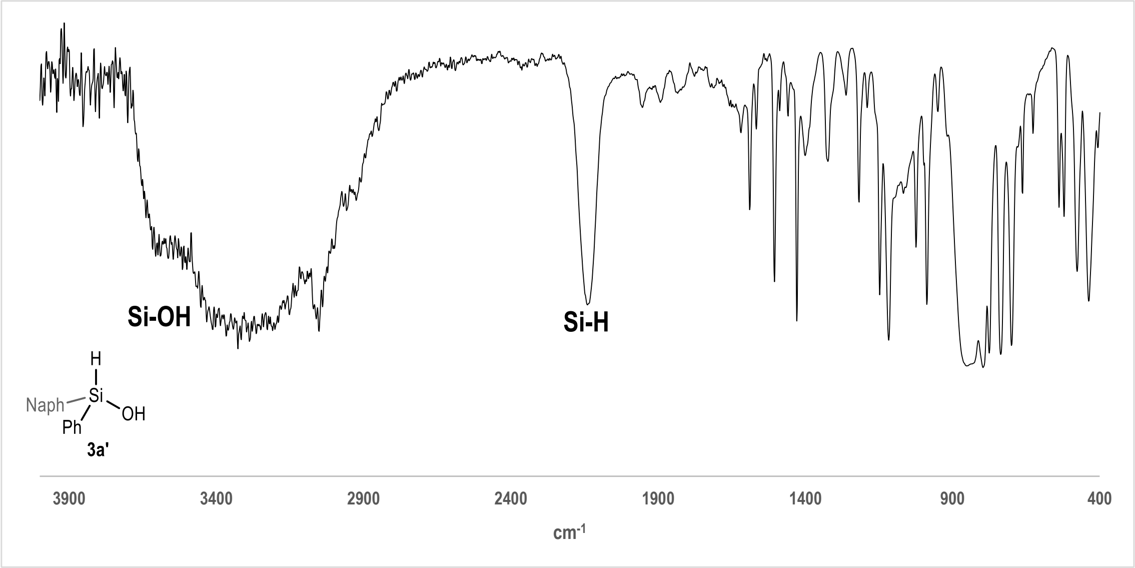

Figure S.61. FTIR spectrum of **3a'**.

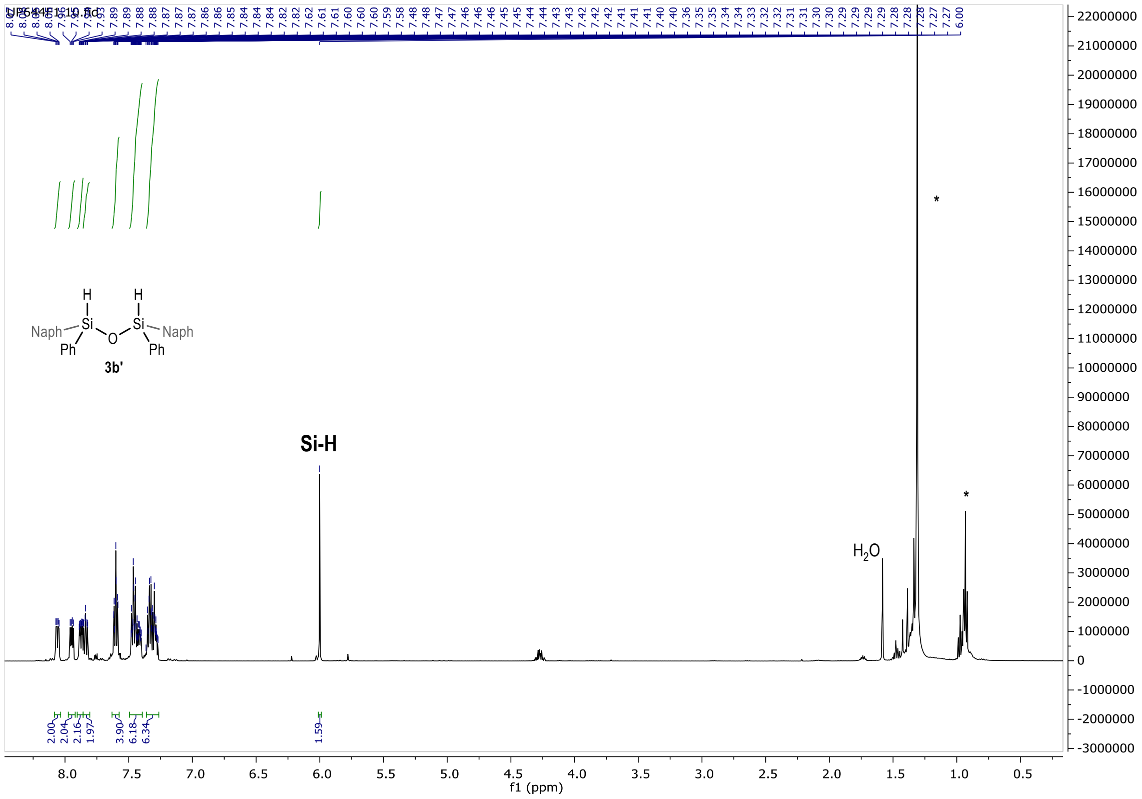

Figure S.62. ^{1}H NMR spectrum in $CDCl_3$ of **3b'**. (*) 10/11 carbon-chain alkane, hexane impurities.

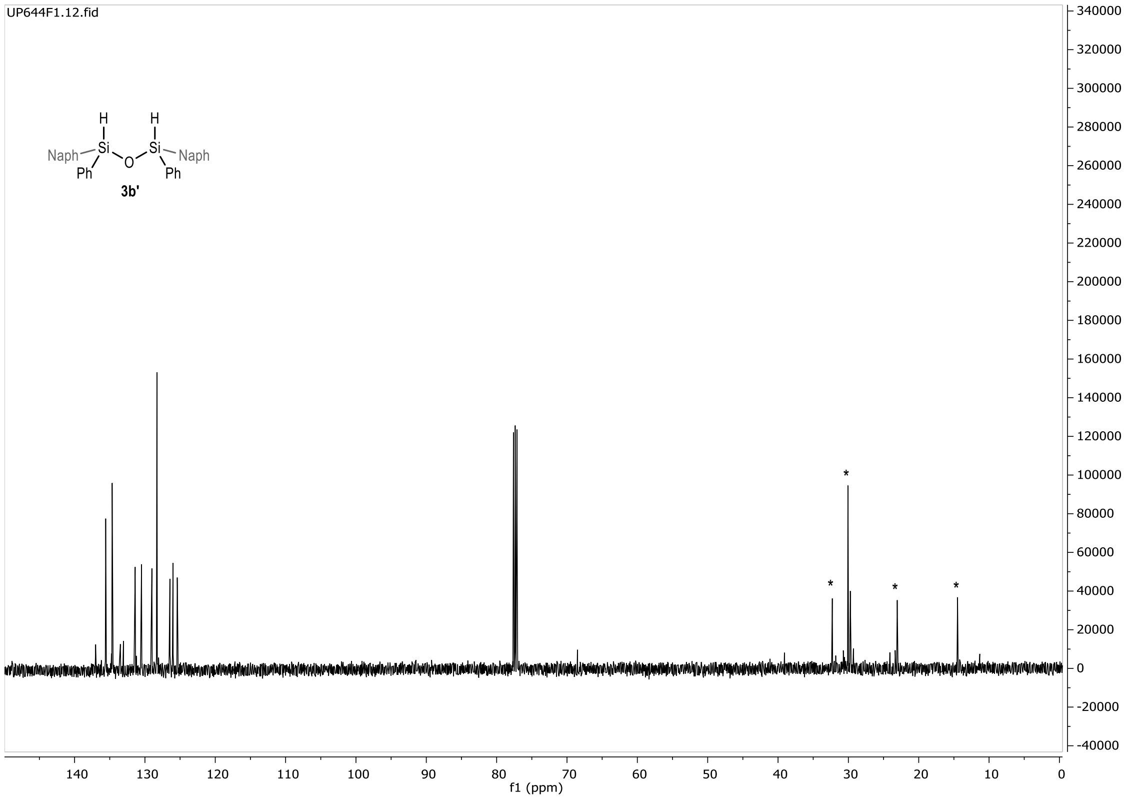

Figure S.63. $^{13}C\{^{1}H\}$ NMR spectrum in $CDCl_3$ of **3b'**. (*) 10/11 carbon-chain alkane, hexane impurities.

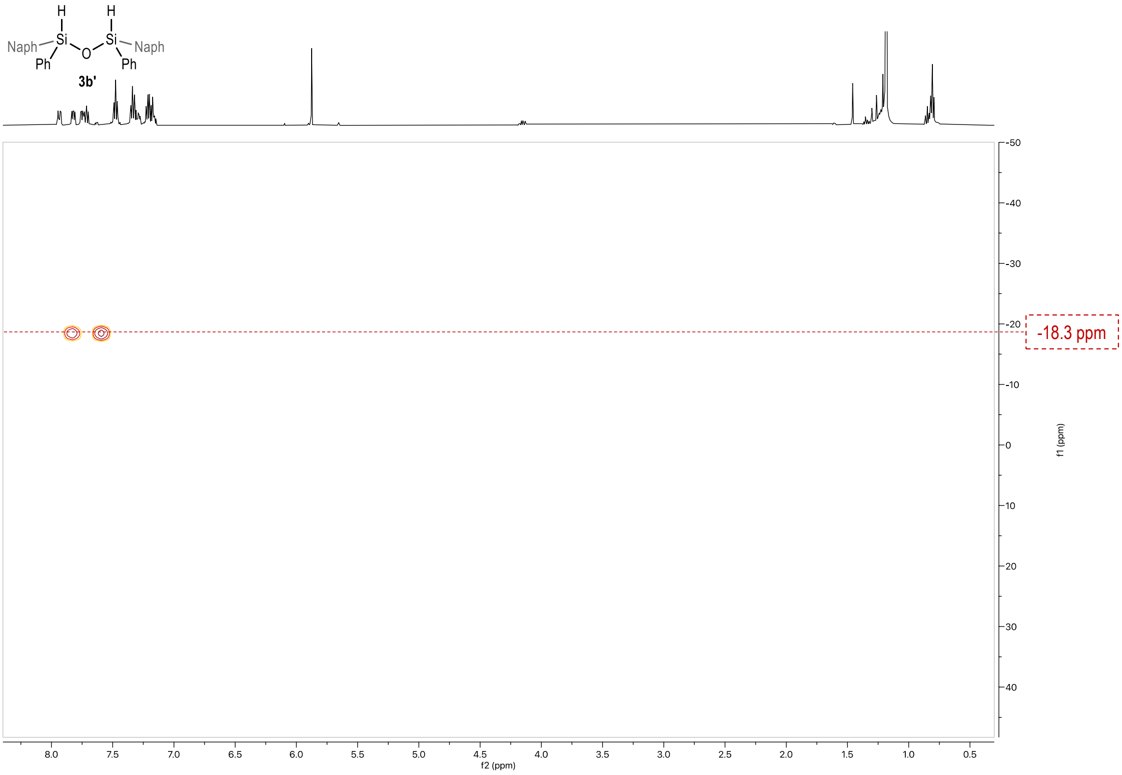

Figure S.64. HMQC (^{1}H - ^{29}Si) NMR spectrum in $CDCl_3$ of **3b'**.

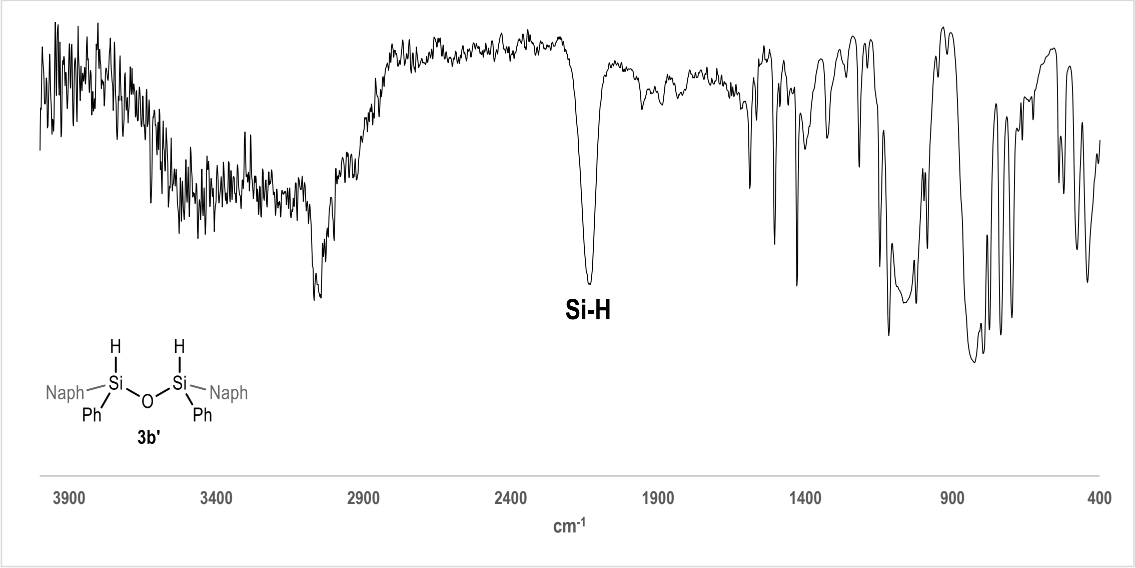

Figure S.65. FTIR spectrum of **3b'**.

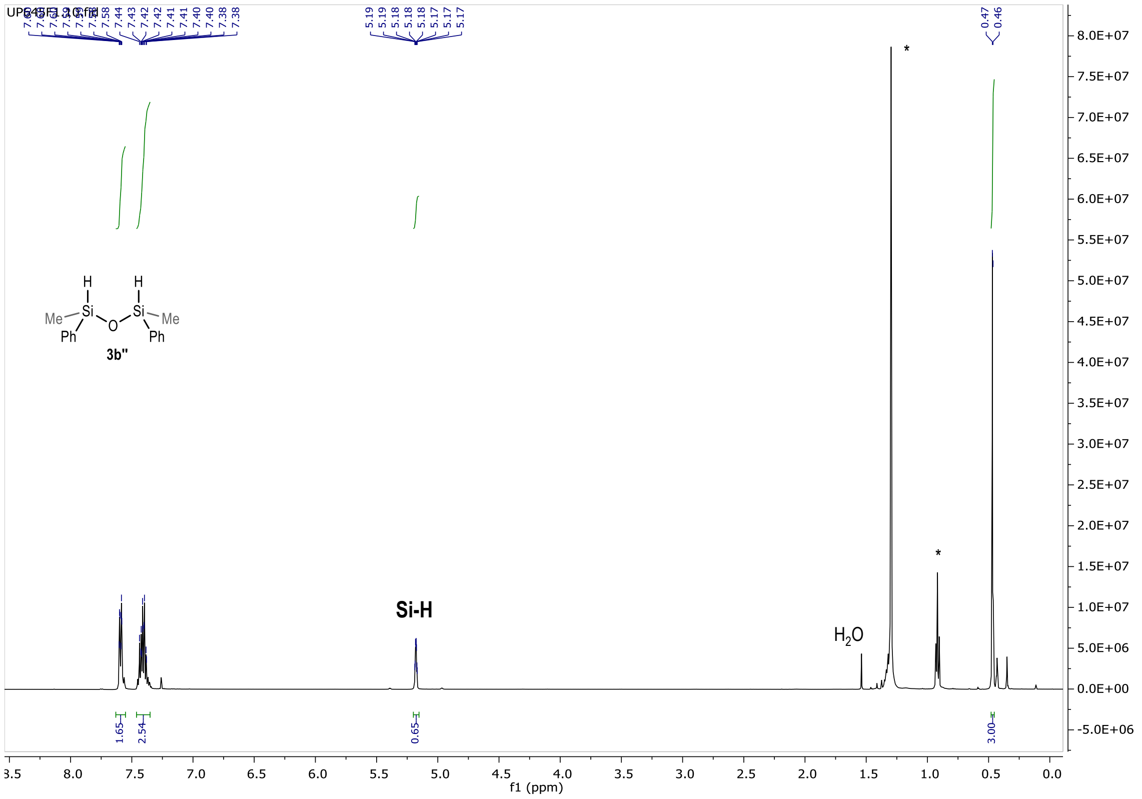

Figure S.66. ^{1}H NMR spectrum in $CDCl_3$ of **3b''**. (*) 10/11 carbon-chain alkane, hexane impurities.

.

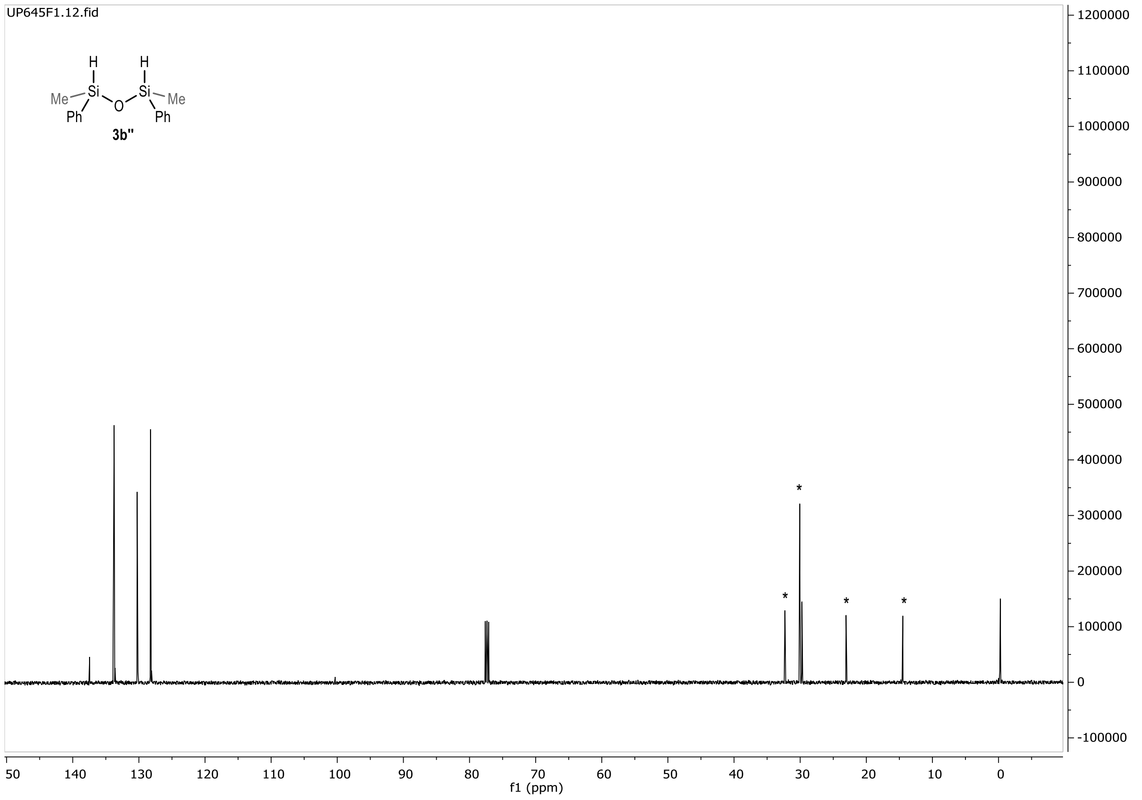

Figure S.67. $^{13}C\{^{1}H\}$ NMR spectrum in $CDCl_3$ of **3b''**. (*) 10/11 carbon-chain alkane, hexane impurities.

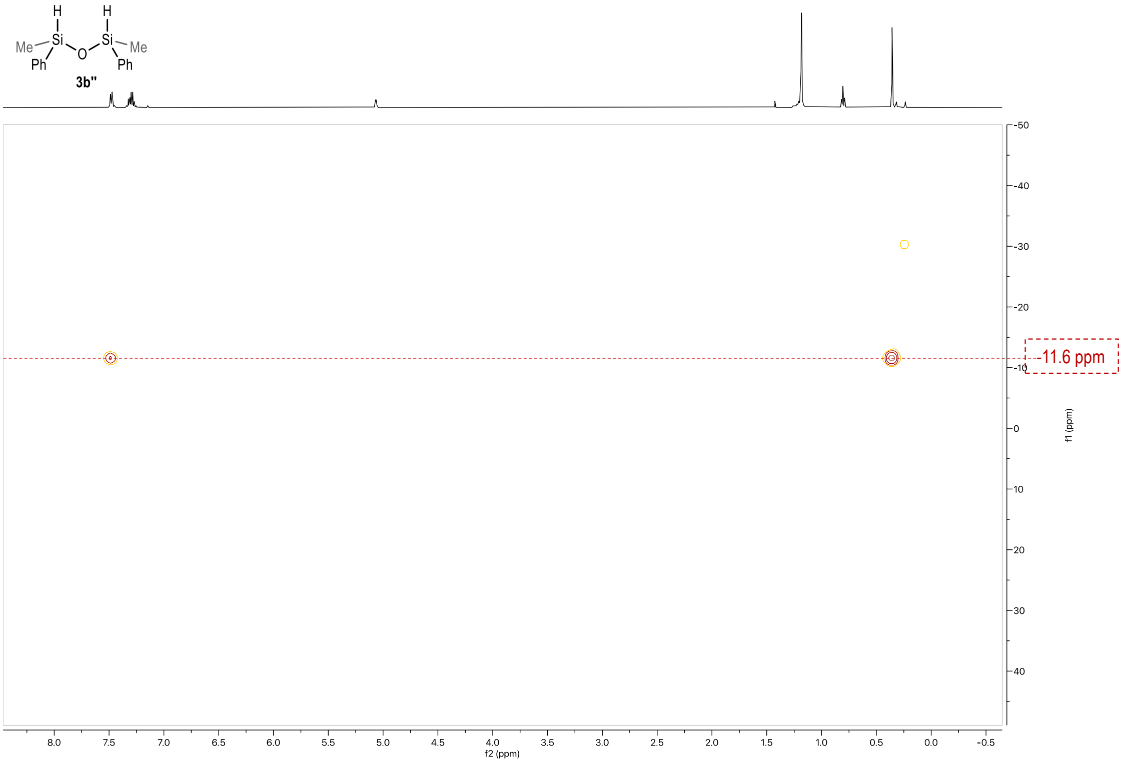

Figure S.68. HMQC (^{1}H - ^{29}Si) NMR spectrum in $CDCl_3$ of **3b''**.

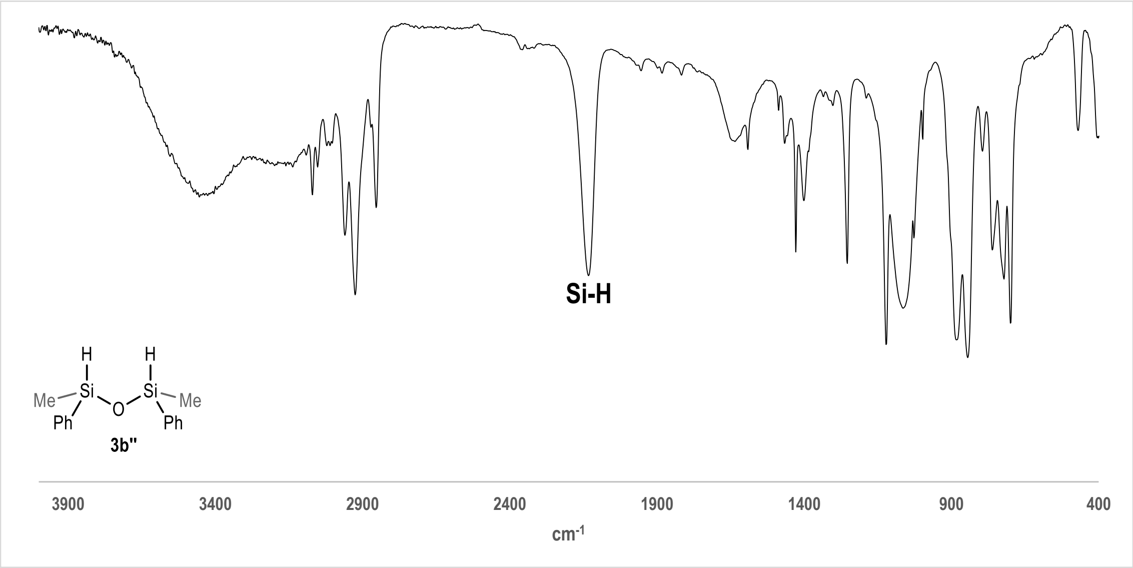

Figure S.69. FTIR spectrum of **3b''**.

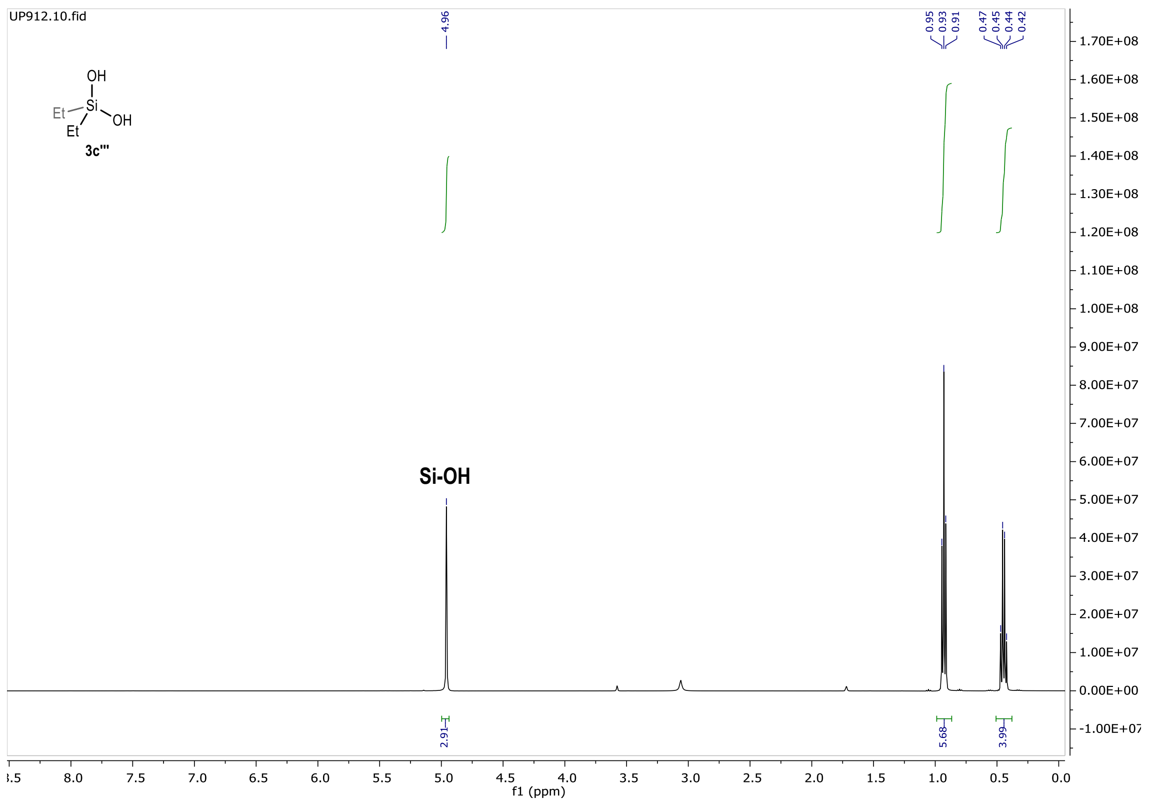

Figure S.70. ^{1}H NMR spectrum in TFH-d$_8$ of **3c'''**.

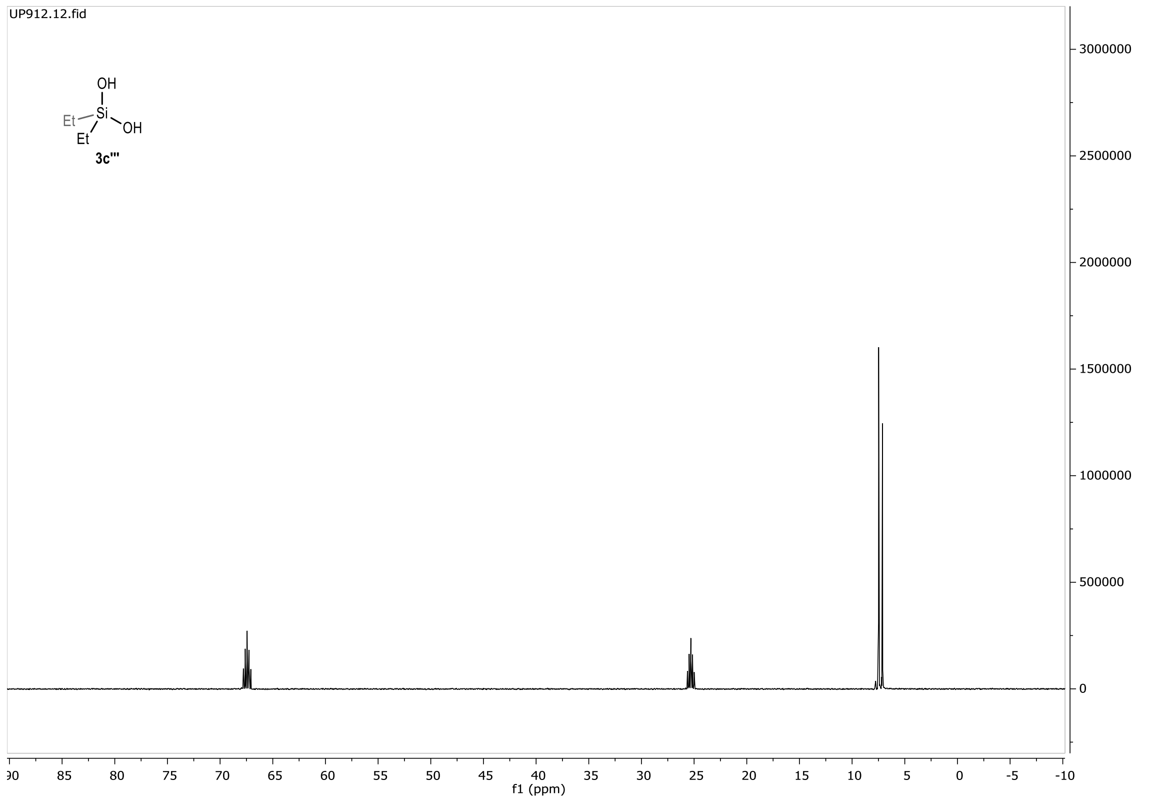

Figure S.71. ^{13}C{^{1}H} NMR spectrum in TFH-d$_8$ of **3c'''**.

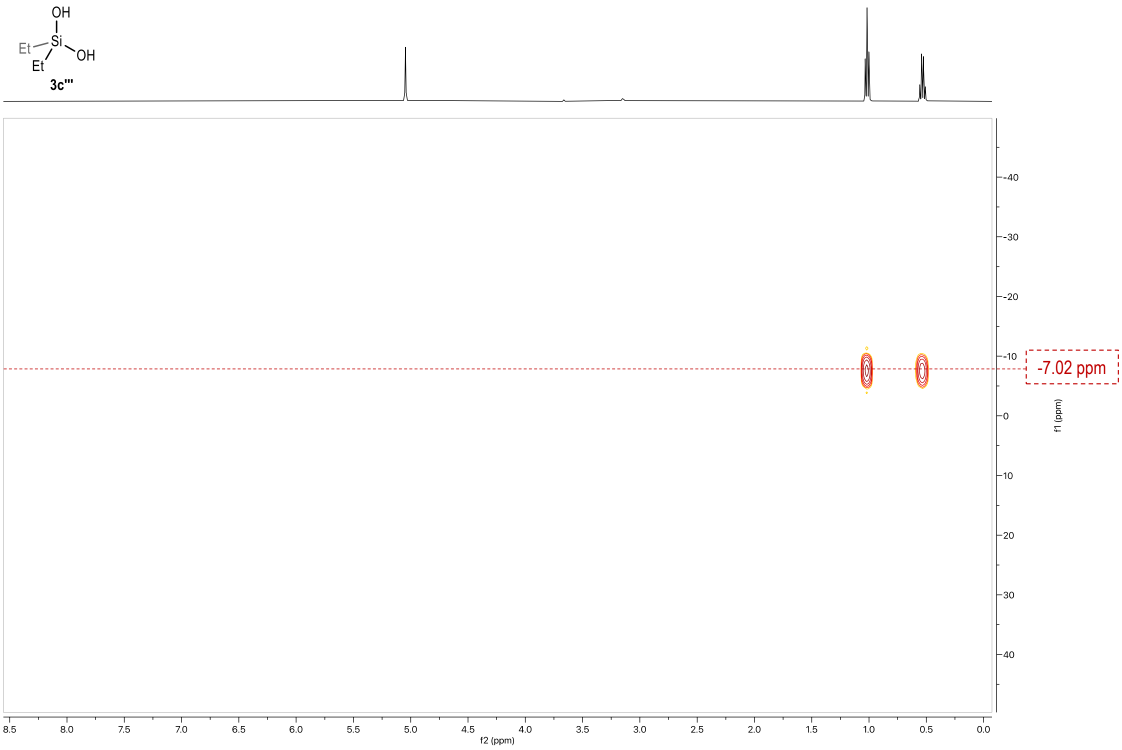

Figure S.72. HMQC (^{1}H - ^{29}Si) NMR spectrum in TFH-d_8 of **3c'''.**

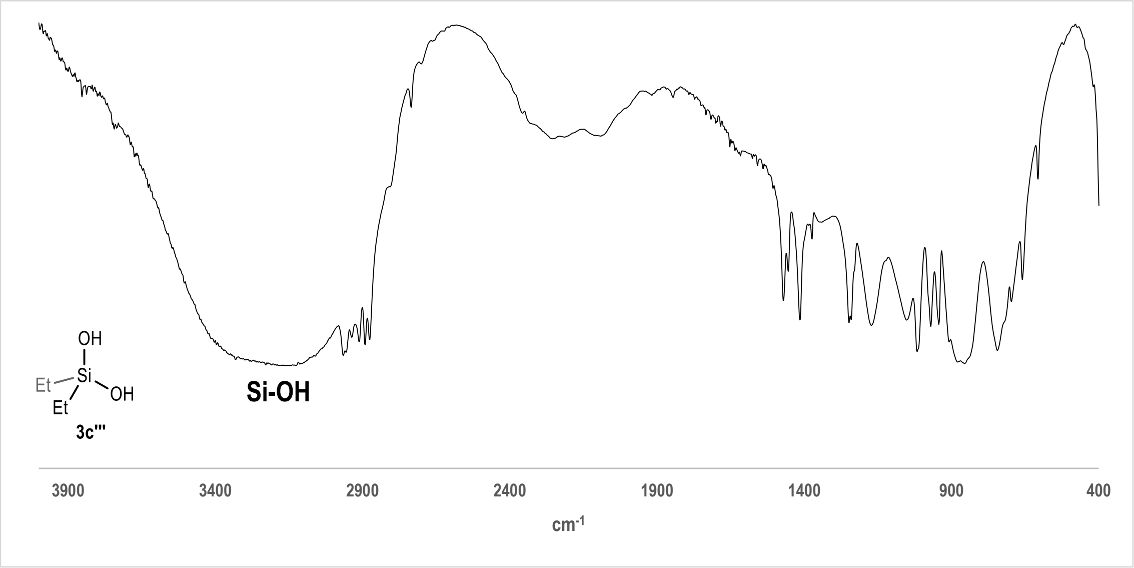

Figure S.73. FTIR spectrum of **3c'''.**
